# Chemical Compositions of *Eupatorium heterophyllum* Leaf Samples from Yunnan and Sichuan Provinces of China—Isolation of 13 New Sesquiterpene Lactones

**DOI:** 10.3390/molecules28135107

**Published:** 2023-06-29

**Authors:** Yiming Hu, Yoshinori Saito, Yasuko Okamoto, Yosuke Matsuo, Xun Gong, Takashi Tanaka

**Affiliations:** 1 Graduate School of Biomedical Sciences, Nagasaki University, 1-14 Bunkyo-Machi, Nagasaki 852-8521, Japan; 2 Faculty of Pharmaceutical Sciences, Tokushima Bunri University, Yamashiro-cho, Tokushima 770-8514, Japan; 3 Kunming Institute of Botany, Chinese Academy of Sciences, Kunming 650201, China

**Keywords:** *Eupatorium heterophyllum*, Asteraceae, sesquiterpene lactone, structure elucidation, intra-specific diversity

## Abstract

Eight samples of *Eupatorium heterophyllum* leaves were collected at different locations in Yunnan and Sichuan provinces in China, and their chemical constituents were investigated. Thirteen previously undescribed sesquiterpene lactones—seven germacranolides, three eudesmanolides, two guaianolides, and a 2-norelemanolide—were isolated, and their structures were elucidated based on extensive spectroscopic analyses. The major constituents in the six samples from northwestern Yunnan and Sichuan are hiyodorilactones A and B, whereas that in the two samples from the region near Kunming, Yunnan is eupatoriopicrin. These results and previously reported results suggest the presence of locality-dependent intra-specific diversity in the chemical constituents of *E. heterophyllum* leaves.

## 1. Introduction

The Hengduan Mountains and surrounding areas consist of many mountain ranges and deep river valleys with the elevation range from approximately 1500 to 6000 m, which separate these areas into various subdivisions. These areas are also climatically diverse and rich in plant resources, providing us with good plant materials for the study of diversity in secondary metabolites.

*Eupatorium heterophyllum* DC. (Asteraceae) is an endemic species in China. It is widely distributed in grasslands and forest areas at altitudes of 1700–3000 m in Southwest China (Sichuan, Yunnan, and Guizhou provinces and Xizang Autonomous Region) and has not been artificially cultivated [1]. We have been studying the intra-specific diversity in leaf and root chemicals of *E. heterophyllum* native to Yunnan and Sichuan provinces [2,3,4,5,6] as part of our continuing research on the chemical diversity of Asteraceae plants for obtaining unique secondary metabolites produced by limited populations within a species and for getting an insight into a chemical aspect of adaptation/differentiation. To date, diversity was observed in the minor constituents of the root chemicals of *E. heterophyllum* samples taken from different locations, affording various heterocyclic aromatic compounds, such as benzofuran/dihydrobenzofuran derivatives and propynyl thiophenes, in addition to some common major constituents [2,3,4]. In particular, seven oligomeric benzofurans were recently isolated for the first time from the sample collected in Lijiang City of Yunnan Province, which suggest an ongoing diversification of secondary metabolites in this species [4,5]. The chemical composition of the leaves, which is mainly composed of sesquiterpene lactones, is significantly different from that of the roots. Our previous study has suggested the presence of a higher degree of chemical diversity in the leaves than that in the roots: hiyodorilactones A (**1**) and B (**3**), which were marked major constituents, and several germacranolides with a hydroperoxy group at C-1β were found in the leaf samples from northwestern Yunnan and southwestern Sichuan (this chemotype is referred to as hiyodorilactone-type), whereas almost no hiyodorilactones and a small amount of eupatoriopicrin (**26**) were detected in the sample from a region near Kunming [6]. However, the overall chemical composition of the samples from the latter region has not been sufficiently clarified due to the presence of many unstable or conformationally flexible constituents. Therefore, further phytochemical studies using newly collected samples are required for understanding the chemical diversity of *E. heterophyllum* leaves. In this study, eight additional samples of *E*. *heterophyllum* leaves were collected in Yunnan (samples 1, 2, 7, and 8) and Sichuan (samples 3–6) provinces of China, and a detailed phytochemical study for each sample was performed. Herein, we report the isolation and structure elucidation of thirteen new sesquiterpene lactones from MeOH extracts of the collected samples as well as the differences in chemical compositions of them.

## 2. Results and Discussion

### 2.1. LC-MS Analysis

**Figure 1:**
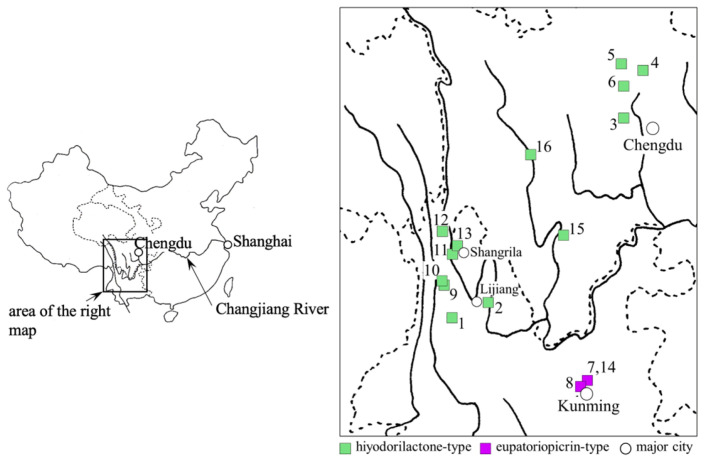
Locations of the collected samples of *E. heterophyllum* (green and purple squares). Samples 9–16 are samples 1–8 from our previous report [6]. Solid and dotted lines indicate rivers and boundaries of provinces, respectively.

**Figure 2:**
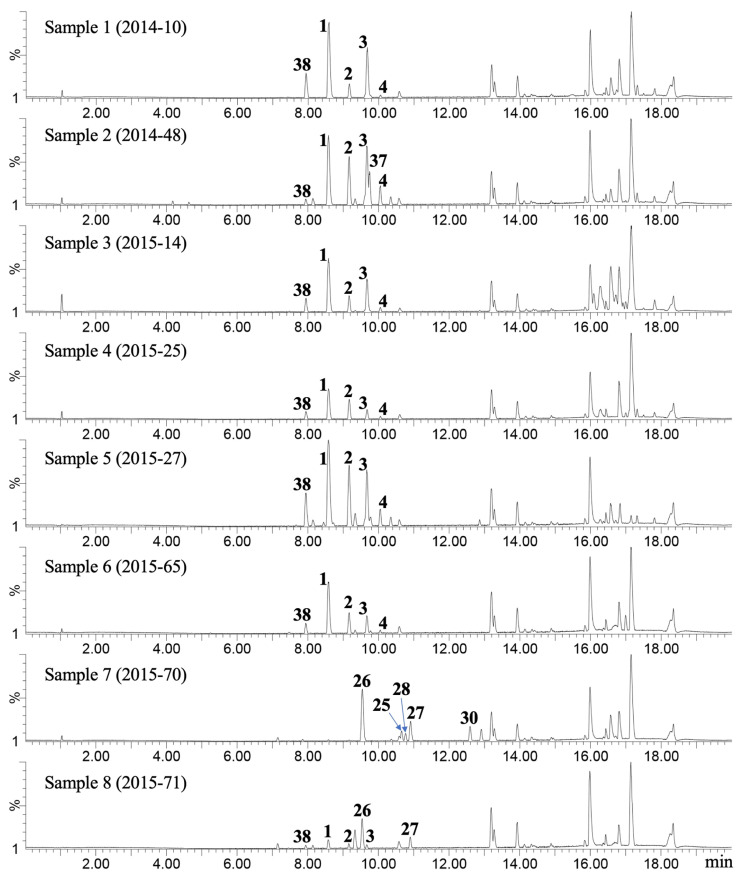
Base peak ion chromatograms of *E. heterophyllum* leaf samples (ESI, positive ion mode). Samples 1–6 were from northwestern Yunnan and Sichuan, and samples 7 and 8 from a region near Kunming of Yunnan.

**Figure 3:**
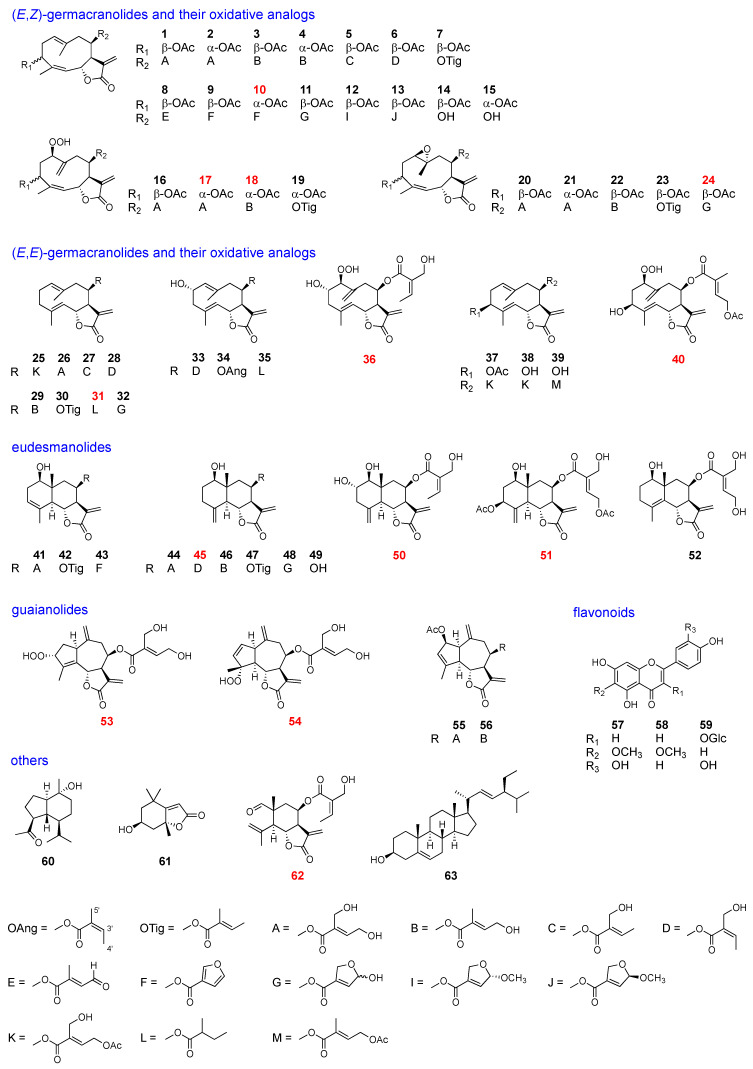
Compounds isolated from the leaves of *E. heterophyllum* (new compounds shown in red).

For an initial assessment of chemical diversity, a portion of each fresh leaf sample collected at the locations shown in Figure 1 was immediately extracted with EtOH, and the chemical composition of the extract was analyzed using LC-MS. The base peak ion (BPI) chromatograms of the samples are shown in Figure 2. Major peaks were identified using isolated compounds (Figure 3). The samples from northwestern Yunnan (samples 1 and 2) and Sichuan (samples 3–6) showed major peaks corresponding to hiyodorilactone D (**38**) [7], hiyodorilactone A (**1**) [8], eupaformosanin (**2**) [9], hiyodorilactone B (**3**) [8], and 20-desoxyeupaformosanin (**4**) [10] at *t*_R_ = 7.95, 8.58, 9.17, 9.68, and 10.05 min, respectively, indicating that these chemical compositions are of hiyodorilactone-type samples. In contrast, the BPI chromatograms of the samples taken from a region near Kunming (samples 7 and 8) showed a significant peak of eupatoriopicrin (**26**) [11] at *t*_R_ = 9.54 min, suggesting the presence of two chemotypes in the samples. There were some differences in the minor peaks of samples 7 and 8. Several peaks derived from (*E*,*E*)-germacranolides (**25** [12], **28** [13], **27** [14], and **30** [15]) were detected at *t*_R_ = 10.66, 10.77, 10.90, and 12.59 min, respectively, for sample 7. Weak peaks characteristic of hyodorilactone-type samples were observed for sample 8 but not for sample 7.

### 2.2. Isolation and Structural Elucidation of Leaf Chemicals

Dried leaves of each sample were extracted with MeOH, and the compounds were separated using silica-gel column chromatography and normal phase HPLC to yield 63 compounds, 13 of which were previously unreported. The isolated compounds were categolized into six types: (*E*,*Z*)-germacranolides and their oxidative analogs, (*E*,*E*)-germacranolides and their oxidative analogs, eudesmanolides, guaianolides, flavonoids, and others, as listed in Figure 3 and Table 1.

Among the isolated compounds, the following were known: hiyodorilactone A (**1**) [8], eupaformosanin (**2**) [9], hiyodorilactone B (**3**) [8], 20-desoxyeupaformasanin (**4**) [10], eupasimplicins B (**5**) [16,17], eupachinsin B (**6**) [18], 3β-acetoxy-8β-tigloyloxyheliangolide (**7**) [19], 4′-dehydrochromolaenide (**8**) [20], santhemoidin A (**9**) [21], 20-dehydroeucannabinolide-semi acetal (**11**) [10], santhemoidin B (**12**) [21], 4′-epi-santhemoidin B (**13**) [19], hiyodorilactone C (**14**) [8], eupaformonin (**15**) [22], hydroperoxyheterophyllin A (**16**) [6], hydroperoxyheterophyllin H (**19**) [6], epoxyeucannabinolid (**20**) [23], 1β,10α-epoxyeupaformosanin (**21**) [9], eupalinin B (**22**) [24], heliangin-3-*O*-acetate (**23**) [25], 8β-(4′-acetoxy-5′-hydroxytigloyloxy)-costunolide (**25**) [12], eupatoriopicrin (**26**) [11], 8β-(5′-hydroxytigloyloxy)-costunolide (**27**) [14], eupaglehnin C (**28**) [13], 20-desoxyeupatoriopicrin (**29**) [10], 8β-tigloyloxycostunolide (**30**) [15], 20-dehydroeupatoriopicrin-semi acetal (**32**) [10], deacetyleupaserrin (**33**) [26], 2α-hydroxyeupatolide-8-*O*-angelate (**34**) [27], 2α-hydroxy-8β-(2-methylbutyryloxy)-germacra-1(10)*E*,4*E*,11(13)-trien-12,6α-olide (**35**) [28], 8β-(4′-acetoxy-5′-hydroxytigloyloxy)-novanin (**37**) [19], hiyodorilactone D (**38**) [7], 4*E*-deacetyl chromolaenide-4′-*O*-acetate (**39**) [29], 1-hydroxy-8-(4′,5′-dihydroxytigloyloxy)-3,11(13)-eudesmadien-6,12-olide (**41**) [12], 1β-hydroxy-8β-tiglinoyloxyarbusculin B (**42**) [25], 1-hydroxy-8-furoyloxy-eudesma-3,11(13)-dien-6,12-olide (**43**) [30], 1-hydroxy-8-(4,5-dihydroxytiglyloxy)-eudesma-4(15),11(13)-dien-6,12-olide (**44**) [30], l-hydroxy-8-sarracenyloxyeudesma-4(15),11(13)-dien-6,12-olide (**46**) [31], 8β-tiglinoyloxyreynosin (**47**) [25], 1-hydroxy-8-(3-[2,5-dihydro-5-hydroxy]-furoyloxy)-eudesma-4(15),11(13)-dien-6,12-olide (**48**) [30], 8β-hydroxyreynosin (**49**) [32], 1-hydroxy-8-(4′,5′-dihydroxytigloyloxy)-4,11(13)-eudesmadien-6,12-olide (**52**) [12], eupahakonesin (**55**) [33], eupachifolin C (**56**) [34], eupafolin (**57**) [35], hispidulin (**58**) [36], quercetin-3-glucoside (**59**) [37], oplopanone (**60**) [38], loliolide (**61**) [39], and stigmasterol (**63**) [40]. The structures of the new compounds (**10**, **17**, **18**, **24**, **31**, **36**, **40**, **45**, **50**, **51**, **53**, **54**, and **62**) were elucidated as follows.

**Figure 4:**
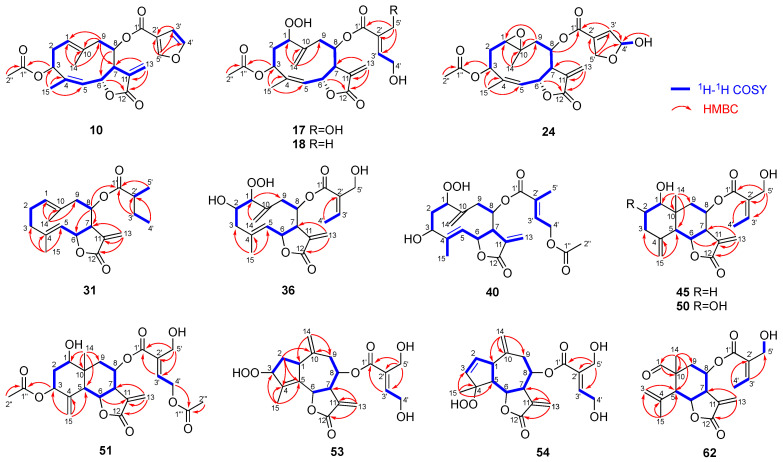
Key ^1^H ^1^H COSY and HMBC correlations for new compounds.

**Figure 5:**
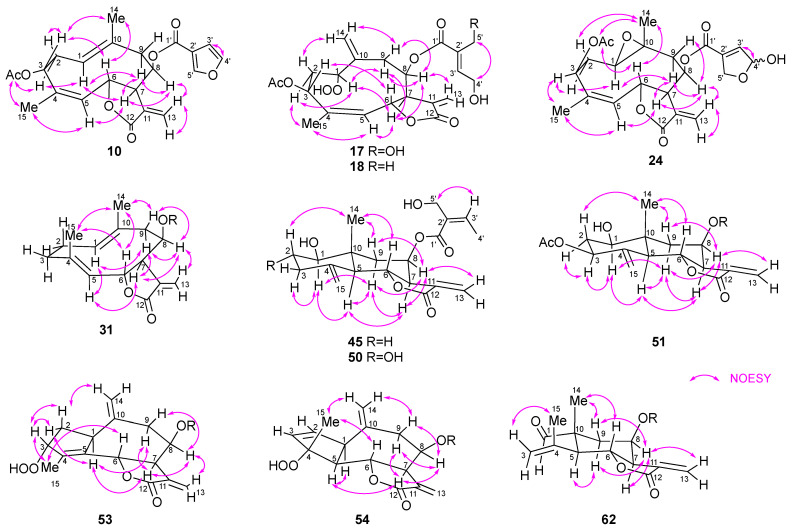
Key NOESY correlations for new compounds.

Compound **10** was obtained as a colorless oil. Its HREIMS spectrum showed the molecular ion peak at *m*/*z* 400.1518 to establish the molecular formula of C_22_H_24_O_7_ with 11 degrees of unsaturation. The IR spectrum of **10** exhibited absorptions at 1765 and 1743 cm^−1^, suggesting the presence of a γ-lactone and an ester group. The ^1^H and ^13^C NMR spectra of **10** (Table 2 and Table 3) were similar to those of santhemoidin A (**9**) [21]. In addition, HMBC correlations from H-3 to C-1″ and from H-6 to C-12 and a NOESY correlation between H-8 and H-13b (δ_H_ 5.80) (Figure 4 and Figure 5) indicated that **10** was a (4*Z*)-germacranolide with an acetoxy group at C-3, a 3-furoyloxy group at C-8, and a γ-lactone between C-12 and C-6, respectively. Thus, **10** has the same planar structure as **9**. However, the signals attributable to H-3 and H-6 were observed at δ_H_ 5.63 and 5.31 in the ^1^H NMR spectrum of **10**, whereas the corresponding signals of **9** were at δ_H_ 5.25 and 5.92, respectively. This characteristic is found between the C-3 epimers of (4*Z*)-germacranolide, hiyodorilactone A (**1**) [8], and eupaformosanin (**2**) [9], which suggests that **10** is the C-3 epimer of **9**. This was confirmed by the NOESY correlations among H-3, H-6, and H_3_-14 (Figure 5). Thus, the structure of **10** was identified as (4*Z*)-3α-acetoxy-8β-(3-furoyloxy)germacra-1(10),4,11(13)-trien-(12,6α)-olide. The absolute configuration was determined to be (3*R*,6*R*,7*R*,8*R*)-**10** because the experimental ECD spectrum of **10** was in good agreement with the theoretical ECD spectrum (Figure S79).

Compound **17** showed a quasimolecular ion [M + H]^+^ at *m*/*z* 453.1764 in its HRFABMS, which suggests a molecular formula of C_22_H_28_O_10_. The ^1^H and ^13^C NMR spectra of **17** (Table 2 and Table 3) resembled those of hydroperoxyheterophyllin A (**16**) [6], suggesting that **17** was a 1β-hydroperoxyheliangolide related to **16**. The major differences between their ^1^H NMR spectra were observed in the chemical shifts of H-3 (**17**: δ_H_ 5.75; **16**: δ_H_ 5.41) and H-6 (**17**: δ_H_ 5.69; **16**: δ_H_ 6.15). These observations were similar to the above-mentioned case of **9** and **10**, indicating an α-orientation of the acetoxy group at C-3 in **17**. This conclusion was supported by the NOE between H-3 and H-6 (Figure 5). Thus, **17** was identified as (4*Z*)-3α-acetoxy-8β-(4′,5′-dihydroxytigloyloxy)-1β-hydroperoxygermacra-4,10(14),11(13)-trien-(12,6α)-olide. In a similar manner, **18** was determined to be a 5′-deoxy derivative of **17**. Its molecular formula C_22_H_28_O_9_ with one less oxygen atom than that of **17**, and the HMBC correlations from H_3_-5′ (δ_H_ 1.82) to C-1′/C-2′/C-3′ support this inference (Figure 4).

The molecular formula of **24** was determined to be C_22_H_26_O_9_ via HRFABMS. Its ^1^H NMR spectrum is similar to that of 1β,10α-epoxyeucannabinolide (**20**) [23] (Table 2), differing only in the signals attributable to the ester group at C-8. The signals δ_H_ 6.66 (m, H-3′), 6.18 (m, H-4′), 4.89 (m, H-5′a), and 4.69 (m, H-5′b) suggested the presence of a 4′,5′-epoxy-4′-hydroxytigloyl group in **24** [10,30]. Moreover, a pair of 4′-OH signals at δ_H_ 3.03/2.94 (each 0.5H, d, *J* = 8.4 Hz) indicated that **24** was a mixture of hemiacetal isomers (*ca*. 1:1). The NOESY correlations shown in Figure 5 suggest that the stereochemistry of the heliangolide core is the same as that of **20**. Thus, **24** was characterized as a C-4′ epimer of (4*Z*)-3β-acetoxy-1β,10α-epoxy-8β-(4′,5′-epoxy-4′-hydroxytigloyloxy)germacra-4,11(13)-dien-(12,6α)-olide.

Compound **31** had the molecular formula of C_20_H_28_O_4_ as indicated by the quasimolecular ion peak at *m*/*z* 333.2059 [M + H]^+^ in its HRCIMS. The ^1^H and ^13^C NMR spectra showed signals corresponding to a 2-methylbutanoyloxy group (Table 2 and Table 3). The remaining signals of **31** were nearly identical to those of the terpene scaffold of 8β-tigloyloxycostunolide (**30**) [15]. The NOESY correlations of H-1/H-5, H-5/H-7, H-7/H-8, H-6/H_3_-14, and H-6/H_3_-15 established the relative configuration of the germacranolide moiety as illustrated in Figure 5. Based on these observations, **31** was identified as 8β-(2′-methylbutanoyloxy)germacra-1(10),4,11(13)-trien-(12,6α)-olide.

Compound **36** showed a [M + K]^+^ peak at *m*/*z* 433.1241 in HRFABMS, confirming its molecular formula as C_20_H_26_O_8_. The IR absorptions at 3380, 1745, and 1715 cm^−1^ suggested the presence of a hydroxy group, γ-lactone, and ester group, respectively. The 1D and 2D NMR spectra of **36** were recorded at 233 K because its ^1^H NMR spectrum exhibited broad signals at room temperature, suggesting conformational flexibility. The ^1^H and ^13^C NMR spectra of **36** (Table 4) were similar to those of deacetyleupaserrin (**33**) [26] except that the signals corresponding to an olefinic methine and a methyl group in **33** were replaced with those of an oxygen-bearing methine [δ_H_ 4.13 (H-1); δ_C_ 97.9 (C-1)] and an exomethylene group [δ_H_ 5.44 and 5.12 (H_2_-14); δ_C_ 120.0 (C-14)], respectively, implying that **36** was a C-1 hydroperoxy analog of deacetyleupaserrin (**33**). This was confirmed by the COSY correlations of H-1/H-2/H_2_-3, H-5/H-6/H-7, H-8/H_2_-9, and H-3′/H-4′, along with the HMBC correlations from H_3_-15 to C-3, C-4, and C-5; from H_2_-14 to C-1; from H-1 to C-9; from H_2_-13 to C-7 and C-12; and from H_3_-4′ to C-2′ (Figure 4). Therefore, the planar structure of **36** was determined as shown in Figure 4. Unfortunately, the NOE correlations required for determining the relative configurations of **36** were not observed; nevertheless, considering the stereochemistry of **33** and other 1-hydroperoxy germacranolides found in this plant, **36** was concluded as 1β-hydroperoxy-2α-hydroxy-8β-(5′-hydroxyangeloyloxy)germacra-4,10(14),11(13)-trien-(12,6α)-olide.

The HRESIMS spectrum of **40** showed a [M + Na]^+^ peak at *m*/*z* 459.1632 to establish a molecular formula of C_22_H_28_O_9_ with nine degrees of unsaturation. The IR absorptions at 3400 cm^−1^ corresponded to a hydroxy group and those at 1743, 1735, and 1715 cm^−1^ are attributed to carbonyl groups. Similar to the case of **36**, the ^1^H NMR spectrum of **40** also afforded broad signals at room temperature. Even at 233 K, the quality of the ^13^C NMR spectrum remained insufficient owing to the small amount of **40** obtained; however, the ^1^H NMR spectrum clearly showed pairs of signals (in a ratio of 2:3 based on the integration), suggesting the coexistence of two conformers. A careful analysis of the ^1^H NMR (Table 4) and the ^1^H-^1^H COSY spectra (Figure 4) of both conformers suggested a structural similarity of **40** with 4*E*-deacetyl chromolaenide-4′-*O*-acetate (**39**) [29] as well as the presence of a hydroperoxy group [δ_H_ 8.56 (major) and 8.43 (minor)] and an additional exomethylene [δ_H_ 5.44/5.13 (major) and 5.35/5.02 (minor)]. The differences in the ^1^H NMR spectrum of **40** with that of **39** were attributable to a 1β-hydroperoxy-10(14)-ene structure, as is the case with **36** and **33**. Therefore, **40** was identified as 8β-(4′-acetoxytigloyloxy)-1β-hydroperoxy-3β-hydroxygermacra-4,10(14),11(13)-trien-(12,6α)-olide.

Compound **45** showed a quasimolecular ion [M + H]^+^ at *m*/*z* 363.1808 in its HRFABMS, which suggests a molecular formula of C_20_H_26_O_6_. The ^1^H NMR data suggested a structural similarity of **45** to that of the known eudesmanolide **44** [30] (Table 5); however, the COSY correlation between H-3′ [δ_H_ 6.40 (q, *J* = 7.3 Hz)] and H_3_-4′ [δ_H_ 2.04 (d, *J* = 7.3 Hz)] and the NOESY correlation between H-3′ and H_2_-5′ indicated that the 4′,5′-dihydroxytigloyl group in **44** was replaced with a 5′-hydroxyangeloyl group in **45** (Figure 4 and Figure 5). Therefore, **45** was identified as 1β-hydroxy-8β-(5′-hydroxyangeloyloxy)eudesma-4(15),11(13)-dien-(12,6α)-olide.

The ^1^H and ^13^C NMR spectra of **50** revealed that it is also an eudesmanolide similar to **45**. Its molecular formula was determined to be C_20_H_26_O_7_, one more oxygen atom than **45**, using HRFABMS. In addition, the COSY correlations between H-1 (δ_H_ 3.19)/H-2 (δ_H_ 3.62)/H_2_-3 (δ_H_ 2.66 and 2.11) and the NOE correlation between H-2β and H_3_-14 suggested the presence of another hydroxy group at C-2α in **50** compared to that in **45** (Figure 4 and Figure 5). Thus, **50** was identified as 1β,2α-dihydroxy-8β-(5′-hydroxyangeloyloxy)eudesma-4(15),11(13)-dien-(12,6α)-olide.

The molecular formula of **51** was determined to be C_24_H_30_O_10_ using HRFABMS. Its ^1^H and ^13^C NMR data (Table 5) were closely related to those of eupakirunsin H [41], suggesting that **51** was also a eudesmanolide. The downfield shift of H-3 (δ_H_ 5.20) and H-8 (δ_H_ 5.83) in the ^1^H NMR spectrum as well as the COSY and HMBC correlations shown in Figure 4 indicated that the hydroxy group at C-3 and tigloyloxy group at C-8 in eupakirunsin H were replaced by acetoxy and 4′-acetoxy-5′-hydroxytigloyl groups, respectively, in **51**.

The HRESIMS spectrum of compound **53** showed a [M + Na]^+^ peak at *m*/*z* 415.1368, which suggests the molecular formula C_20_H_24_O_8_ with nine degrees of unsaturation. The ^1^H and ^13^C NMR spectra revealed the presence of one methyl, two oxymethylenes, three oxymethines, two exocyclic double bonds, one trisubstituted double bond, one tetrasubstituted double bond, and two carbonyls (Table 6). The above spectroscopic data accounted for six degrees of unsaturation, and therefore, **53** should be tricyclic. Compound **53** was deduced to be a guaianolide with oxygen-functionalities at C-3, C-6, and C-8, one of which is a 4′,5′-dihydroxytigloyloxy group, as evidenced by the COSY and HMBC correlations shown in Figure 4. A significant downfield shift of H-8 (δ_H_ 5.72) as well as the NOESY correlation between H-13b and H-8 suggested the presence of a 4′,5′-dihydroxytigloyloxy moiety at C-8 and a γ-lactone between C-12 and C-6. Moreover, the molecular formula of **53** and the chemical shift of C-3 (δ 94.2) suggested the presence of a hydroperoxy group at this position. The elucidated planar structure of **53** is shown in Figure 4. The NOESY spectrum showed a cross-peak between H-7 and H-1, H-8, and H-9α, indicating that these hydrogens were in the same orientation (Figure 5). H-3 showed NOE correlations with H-2a and H-2b, but not with H-1, indicating the α-orientation of hydroperoxy group. Finally, H-6 was assigned a β-orientation owing to its coupling constant (*J*_6,7_ = 10.5 Hz). Therefore, **53** was identified as 8β-(4′,5′-dihydroxytigloyloxy)-3α-hydroperoxyguaia-4,10(14),11(13)-trien-(12,6α)-olide.

HRFABMS and 1D/2D NMR spectra of **54** revealed that it is also a guaianolide with the same molecular formula as that of **53** (Table 6 and Figure 4 and Figure 5). A COSY correlation between two olefinic protons at C-2 (δ_C_ 133.0; δ_H_ 5.77) and C-3 (δ_C_ 137.2; δ_H_ 6.01) indicated a disubstituted double bond. The chemical shift of C-4 observed at δ_C_ 95.2 in the ^13^C NMR spectrum and NOE correlation between H_3_-15 and H-6 indicated the presence of a hydroperoxy group at C-4α. Thus, **54** was concluded to be a 4α-hydroperoxy-2-ene isomer of **53**.

Compound **62** was isolated as a colorless oil. A [M + Na]^+^ peak was observed at *m*/*z* 371.1472 in its HRESIMS, corresponding to the molecular formula of C_19_H_24_O_6_ with eight degrees of unsaturation. The IR spectrum suggested the presence of hydroxy (3501 cm^−1^), γ-lactone (1769 cm^−1^), aldehyde (1726 cm^−1^), and α,β-unsaturated carbonyl (1715 cm^−1^) groups. The ^1^H and ^13^C NMR spectra showed the characteristic signals for a 5′-hydroxyangeloyl moiety (Table 6), which implied that **62** was a norsesquiterpenoid. The ^1^H-^1^H COSY spectrum exhibited a spin system from H-5 to H_2_-9 (Figure 4). Furthermore, in the HMBC spectrum, H_3_-14 was correlated with C-1/C-5/C-9/C-10 and H_3_-15 with C-3/C-4/C-5, indicating that **62** was a 2-norelemanolide. A downfield shift of H-8 (δ_H_ 5.87) as well as the NOE between H-8 and H-13b (δ_H_ 5.57) as shown in Figure 5 confirmed the position of an α-methylene-γ-lactone and 5′-hydroxyangeloyl group. The relative stereochemistry of **62** is similar to that of **45** based on NOE correlations and coupling constants. Thus, **62** was identified as 8β-(5′-hydroxyangeloyloxy)-1-oxo-2-norelema-3,11(13)-dien-(12,6α)-olide.

The experimental ECD spectra of **17**, **18**, **24**, **45**, **50**, **51**, **54**, and **62** showed a similar trend to that of **10**, especially the negative Cotton effect around 210 nm mainly owing to the α-methylene-γ-lactone moiety. This indicated that the absolute configurations at C-6 and C-7 of these compounds are the same as those of **10** while the other chromophore might have a weaker contribution to their experimental ECD spectra [18]. In addition, considering the biosynthesis of sesquiterpenoids in higher plants, the other new compounds, **31**, **36**, **40**, and **53,** would have the same stereochemistry.

### 2.3. Discussion

In this study, 63 compounds, including 13 new compounds, were isolated from 8 leaf samples of *E. heterophyllum* collected in Yunnan and Sichuan provinces (Figure 3). Among the isolated compounds, 57 compounds were sesquiterpene lactones, including germacranolides (**1**–**40**), eudesmanolides (**41**–**52**), guaianolides (**53**–**56**), and elemanolides (**62**). Most of the new compounds would be produced from major constituents vir oxidative metabolism, which often involve the introduction of a hydroperoxy group. The chemical composition of each sample is summarized in Table 1. The major peaks in the BPI chromatograms (Figure 2) confirm the major constituents in each sample. Hiyodorilactones A (**1**) and B (**3**) are the major constituents in samples 1–6, confirming that hiyodorilactone-type samples are predominant in Sichuan and northwestern Yunnan regions. In contrast, eupatoriopicrin (**26**), which is not detected in samples 1–6, is the major constituent in samples 7 and 8 from the region near Kunming. A variety of (*E*,*E*)-germacranolides and eudesmanolides are also contained in these samples. Therefore, samples 7 and 8 should be classified as another chemotype, eupatoriopicrin-type. Considering these results and previously obtained results [6], we conclude the presence of locality-dependent intra-specific diversity in the leaf chemicals of *E. heterophyllum*.

Eupatoriopicrin (**26**) showed higher cytotoxicity against HL-60 cells than hyodorilactones A (**1**) and B (**3**) [42]. Moreover, Pan et al. recently reported genetic diversity in *E. heterophyllum* [43]. Notably, the geographical distribution of the different genotypes is in good agreement with the chemotypes observed in this study, indicating that the intra-specific diversity in the leaf chemicals of *E. heterophyllum* is related to its genetic background. These observations can provide us a new insight into a chemical aspect of adaptation and differentiation of *Eupatorium* plants. We plan to conduct further chemical studies on *E. heterophyllum* sampled from other regions to obtain secondary metabolites produced by limited populations within a species and to understand the relationship between chemical diversity, genetic diversity, and geographical distribution.

## 3. Materials and Methods

### 3.1. General Experimental Procedures

Optical rotations were measured with a JASCO P1020NK digital polarimeter. IR spectra were recorded using a JASCO FT/IR-410 spectrophotometer with the diffuse reflectance method. The 1D and 2D NMR spectra were measured using a Varian Unity *plus* 500 spectrometer (^1^H: 500 MHz; ^13^C: 126 MHz) or JEOL JNM-AL 400 spectrometer (^1^H: 400 MHz; ^13^C: 100 MHz). The coupling constants (*J*) are expressed in Hertz, and the chemical shifts (δ) are reported in ppm with the residual solvent signal used as a reference (CDCl_3_: TMS). Mass spectra, including high-resolution spectra, were recorded on a JEOL JMS-700 MStation. Column chromatography was performed on silica gel 60 (100–210 mesh, Kanto Chemical Co., Inc., Tokyo, Japan). TLC was with silica gel 60 F254 plates (Merck, Rahway, NJ, USA). Preparative HPLC was performed on a JASCO chromatograph (*n*-hexane–EtOAc, CHCl_3_–EtOAc, CHCl_3_–MeOH) equipped with a JASCO PU-2086 pump, a JASCO UV-970 detector, a JASCO RI-2031 detector, and various columns: COSMOSIL 5SL-II (20 × 250 mm, Nacalai Tesque Inc., Kyoto, Japan), COSMOSIL 5SL-II (10 × 250 mm, Nacalai Tesque Inc., Kyoto, Japan), COSMOSIL 5SL-II (4.6 × 250 mm, Nacalai Tesque Inc., Kyoto, Japan), YMC-Pack Diol-120-NP (4.6 × 250 mm, YMC Co., Ltd., Kyoto, Japan), Inertsil CN-3 (4.6 × 250 mm, GL Sciences, Tokyo, Japan), Inertsil Diol (10 × 250 mm, GL science, Tokyo, Japan), TSK gel silica 60 (4.6 × 250 mm, Tosoh, Tokyo, Japan), and TSK gel G1000H_HR_ (7.8 × 300 mm, Tosoh, Tokyo, Japan).

### 3.2. Plant Materials

Samples were collected in August of 2014 (samples 1 and 2) and 2015 (samples 3–8) at the several geographically isolated locations shown in Figure 1 and Table 7. Each sample was authenticated by Dr. Takayuki Kawahara, Japan Forest Technology Association, General Incorporated Association, Japan. The voucher specimens (No. 2014-10, 2014-48, 2015-14, 2015-25, 2015-27, 2015-65, 2015-70, and 2015-71 for samples 1–8, respectively) are deposited in Kunming Institute of Botany, Kunming, China.

### 3.3. LC-MS Analysis

Parts of the fresh leaves (a few grams) of each sample were extracted with ethanol immediately after harvesting, and the extracted ethanol solutions were filtered and then separated using a Waters Acquity™ UPLC I-Class system coupled with a Waters ACQUITY UPLC BEH C18 column (2.1 × 100 mm, 1.7 μm). The temperature of the column was held at 45 °C. The mobile phases, which consisted of two solvent systems (eluent A, 0.1% formic acid in the water, *v*/*v*; eluent B (0.1% formic acid in methanol, *v*/*v*)), were delivered at a flow rate of 0.25 mL/min using a linear gradient program. The linear elution gradient program was set as follows: 0 min (95:5)–14.75 min (2:98)–17.00 min (2:98)–17.20 min (95:5)–20.00 min (95:5).

A Waters SYNAPT G2-Si HDMS mass spectrometer was connected to the UPLC system via an ESI interface. The conditions of analysis were as follows: capillary voltage was set at 1.5 kV under positive ion and 2.0 kV under negative mode, sampling cone voltage at 40.0 V, source temperature at 120 °C, desolvation gas temperature at 500 °C. The cone gas flow was at 500 L/h, desolvation gas at 1200 L/h, and the nebulizer gas at 6.5 bar.

### 3.4. Extraction and Isolation

The dried leaves of sample 1 (39.1 g) were cut into small pieces and extracted with MeOH two times at room temperature. After removal of the solvent under reduced pressure not exceeding 30 °C, a concentrated and combined MeOH extract (7.5 g) was obtained, which was then separated using a silica gel column (40 × 160 mm, *n*-hexane–EtOAc, 9:1, 8:2, 7:3, 6:4, 1:1, 2:8; EtOAc–MeOH, 1:0, 95:5, 9:1, 7:3, 0:1) to afford ten subfractions (Fr. 1–10). Compound **1** (573.7 mg) was obtained as Fr. 7. Fr. 4 (165.1 mg) was purified with semipreparative HPLC (COSMOSIL 5SL-II, 10 × 250 mm, *n*-hexane–EtOAc, 8:2) to give **7** (11.0 mg) and **9** (36.4 mg). Fr. 5 (141.7 mg) was suspended in *n*-hexane–EtOAc (7:3) and then filtered to obtain **58** (6.3 mg) as a precipitate. The filtrate was separated by COSMOSIL 5SL-II (10 × 250 mm, *n*-hexane–EtOAc, 7:3) to afford **58** (7.8 mg), Fr. 5-4 (9.5 mg), and **14** (9.5 mg). Fr. 5-4 (9.5 mg) was purified on YMC-Pack Diol-120-NP (4.6 × 250 mm, *n*-hexane:EtOAc, 7:3) to yield **60** (0.7 mg), a mixture (4:3) of **12** and **13** (2.1 mg), and **15** (0.4 mg). Fr. 6 (670.1 mg) was separated by COSMOSIL 5SL-II (20 × 250 mm, *n*-hexane–EtOAc, 4:6) to give five fractions: Fr. 6-0–6-4. Fr. 6-4 (47.6 mg) was identified as **38**. Fr. 6-2 (251.5 mg) was purified on COSMOSIL 5SL-II (20 × 250 mm, CHCl_3_–EtOAc, 7:3) and gave **11** (83.9 mg) and **3** (112.5 mg). Fr. 6-3 (90.3 mg) was purified on YMC-Actus Triart Diol-HILIC (20 × 250 mm, CHCl_3_–EtOAc, 6:4) and Inertsil CN-3 (4.6 × 250 mm, *n*-hexane–EtOAc, 1:1) to obtain **22** (2.1 mg) and **24** (1.9 mg).

The extract of sample 2 (5.4 g from 31.2 g of leaves) was similarly treated to afford **1** (187.7 mg), **2** (64.3 mg), **3** (85.4 mg), **4** (18.8 mg), **7** (9.6 mg), **8** (3.2 mg), **9** (38.1 mg), **10** (17.9 mg), **11** (20.0 mg), **14** (7.9 mg), **15** (10.8 mg), **17** (11.5 mg), **20** (16.6 mg), **21** (21.0 mg), **22** (4.2 mg), **24** (2.8 mg), **37** (28.1 mg), **51** (7.4 mg), **57** (26.5 mg), and **58** (4.3 mg).

The extract of sample 3 (2.3 g from 14.7 g of leaves) was similarly treated to afford **1** (11.2 mg), **3** (68.6 mg), **4** (2.4 mg), **18** (1.5 mg), and **60** (1.7 mg).

The extract of sample 4 (7.8 g from 46.2 g of leaves) was similarly treated to afford **1** (305.1 mg), **2** (129.6 mg), **3** (161.0 mg), **4** (15.5 mg), **5** (0.5 mg), **7** (6.9 mg), **16** (20.4 mg), **17** (6.4 mg), **19** (0.8 mg), **20** (1.9 mg), **40** (0.9 mg), **53** (0.5 mg), **54** (6.8 mg), and **61** (2.1 mg).

The extract of sample 5 (1.0 g from 5.2 g of leaves) was similarly treated to give **1** (101.7 mg), **2** (37.6 mg), **3** (12.9 mg), **4** (2.2 mg), **7** (0.9 mg), **9** (0.9 mg), **11** (4.8 mg), **14** (0.4 mg), **16** (4.8 mg), **17** (2.2 mg), **38** (6.8 mg), **57** (3.5 mg), and **60** (0.5 mg).

The extract of sample 6 (3.1 g from 24.8 g of leaves) was similarly treated to afford **1** (303.8 mg), **3** (34.6 mg), **4** (6.1 mg), **5** (0.6 mg), **6** (5.2 mg), **7** (1.4 mg), **9** (1.2 mg), **11** (8.1 mg), **12** (0.5 mg), **13** (0.3 mg), **14** (3.0 mg), **15** (0.5 mg), **37** (1.6 mg), **38** (16.1 mg), **39** (0.9 mg), **56** (0.7 mg), **57** (5.4 mg), **58** (1.7 mg), and **61** (1.3 mg).

The extract of sample 7 (4.0 g from 25.9 g of leaves) was similarly treated to afford **3** (2.1 mg), **23** (0.6 mg), **25** (22.5 mg), **26** (514.5 mg), **27** (27.1 mg), **28** (73.0 mg), **29** (11.0 mg), **30** (70.4 mg), **31** (23.6 mg), **32** (1.0 mg), **34** (3.1 mg), **35** (4.7 mg), **42** (4.3 mg), **47** (2.6 mg), **57** (16.5 mg), **61** (1.8 mg), and **63** (21.3 mg).

The extract of sample 8 (3.3 g from 26.2 g of leaves) was similarly treated to afford **1** (7.8 mg), **2** (16.3 mg), **3** (8.8 mg), **4** (1.1 mg), **11** (1.0 mg), **16** (0.2 mg), **26** (50.3 mg), **28** (10.0 mg), **29** (1.2 mg), **32** (7.9 mg), **33** (5.6 mg), **36** (2.0 mg), **38** (8.4 mg), **39** (0.4 mg), **41** (6.9 mg), **43** (1.9 mg), **44** (20.0 mg), **45** (4.6 mg), **46** (1.0 mg), **48** (1.9 mg), **49** (0.2 mg), **50** (1.4 mg), **52** (2.3 mg), **55** (0.2 mg), **57** (23.7 mg), **58** (3.4 mg), **59** (68.1 mg), **60** (7.2 mg), **61** (1.0 mg), and **62** (0.4 mg).

### 3.5. Calculation of ECD Spectra

A conformational search was performed using the Monte Carlo method and the MMFF94 force field with Spartan ′20 (Wavefunction, Irvine, CA, USA). The obtained low-energy conformers within 6 kcal/mol were optimized at the B3LYP/6-31G(d,p) level in MeOH (PCM). The vibrational frequencies were also calculated at the same level to confirm their stability, and no imaginary frequencies were found. The energies, oscillator strengths, and rotational strengths of the low-energy conformers were calculated using TDDFT at the CAM-B3LYP/6-31G(d,p) in MeOH (PCM) level and weight-averaged. The ECD spectra were simulated using GaussView [44] with the overlapping Gaussian function with 0.35 eV exponential half-width, and UV correction was performed (redshifted by 10 nm). All DFT calculations were performed using Gaussian 09 [45].

### 3.6. Compound Data

Compound **10**, Colorless oil; [α${]}_{D}^{20}$: −76.6 (*c* = 0.10, MeOH); FT-IR cm^−1^: 3448, 1765, 1743; MS (EI) *m*/*z*: 400 (M)^+^; HRMS (EI) *m*/*z*: 400.1518 (calcd for C_22_H_24_O_7_: 400.1522). UV (CH_3_OH) λ_max_ (log ε) 204 (0.84) nm; ECD (CH_3_OH, *c* = 5.1 × 10^−5^ mol/L) λ_max_ (Δε): 208 (−22.74) nm. ^1^H and ^13^C NMR: see Table 2 and Table 3.
Compound **17**, Colorless oil; [α${]}_{D}^{30}$: +19.0 (*c* = 0.59, MeOH); FT-IR cm^−1^: 3411, 1740, 1715; MS (FAB) *m*/*z*: 453 (M + H)^+^; HRMS (FAB) *m*/*z*: 453.1764 (calcd for C_22_H_29_O_10_: 453.1761). UV (CH_3_OH) λ_max_ (log ε) 204 (1.03) nm; ECD (CH_3_OH, *c* = 4.6 × 10^−5^ mol/L) λ_max_ (Δε): 233 (+1.39), 215 (−6.48) nm. ^1^H and ^13^C NMR: see Table 2 and Table 3.
Compound **18**, Colorless oil; [α${]}_{D}^{30}$: +9.9 (*c* = 0.12, MeOH); FT-IR cm^−1^: 3390, 1739, 1714; MS (FAB) *m*/*z*: 475 (M + K)^+^; HRMS (FAB) *m*/*z*: 475.1383 (calcd for C_22_H_28_O_9_K: 475.1370). UV (CH_3_OH) λ_max_ (log ε) 203 (0.63) nm; ECD (CH_3_OH, *c* = 4.7 × 10^−5^ mol/L) λ_max_ (Δε): 302 (+0.32), 255 (−0.25), 236 (+0.25), 214 (−4.86) nm. ^1^H and ^13^C NMR: see Table 2 and Table 3.
Compound **24**, Colorless oil; [α${]}_{D}^{17}$: −59.8 (*c* = 0.20, MeOH); FT-IR cm^−1^: 3419, 1760, 1730; MS (FAB) *m*/*z*: 457 (M + Na)^+^; HRMS (FAB) *m*/*z*: 475.1475 (calcd for C_22_H_26_O_9_Na: 475.1475). UV (CH_3_OH) λ_max_ (log ε) 203 (0.87) nm; ECD (CH_3_OH, *c* = 4.3 × 10^−5^ mol/L) λ_max_ (Δε): 296 (+0.23), 263 (−0.16), 238 (+0.67), 214 (−10.21) nm. ^1^H and ^13^C NMR: see Table 2 and Table 3.
Compound **31**, Colorless oil; [α${]}_{D}^{15}$: +39.2 (*c* = 0.54, CHCl_3_); FT-IR cm^−1^: 1769, 1731; MS (CI) *m*/*z*: 333 (M + H)^+^; HRMS (CI) *m*/*z*: 333.2059 (calcd for C_20_H_29_O_4_: 333.2066). ^1^H and ^13^C NMR: see Table 2 and Table 3.
Compound **36**, Colorless oil; [α${]}_{D}^{20}$: +105.1 (*c* = 0.15, MeOH); FT-IR cm^−1^: 3380, 1745, 1715; MS (FAB) *m*/*z*: 433 (M + K)^+^; HRMS (FAB) *m*/*z*: 433.1241 (calcd for C_20_H_26_O_8_K: 433.1265). ^1^H and ^13^C NMR: see Table 4.
Compound **40**, Colorless oil; [α${]}_{D}^{30}$: +9.9 (*c* = 0.12, MeOH); FT-IR cm^−1^: 3400, 1743, 1735, 1715; MS (ESI) *m*/*z*: 459 (M + Na)^+^; HRMS (ESI) *m*/*z*: 459.1632 (calcd for C_22_H_28_O_9_Na: 459.1631). ^1^H and ^13^C NMR: see Table 4.
Compound **45**, Colorless oil; [α${]}_{D}^{29}$: +31.6 (*c* = 0.33, CHCl_3_); FT-IR cm^−1^: 3444, 1769, 1715; MS (FAB) *m*/*z*: 363 (M + H)^+^; HRMS (FAB) *m*/*z*: 363.1808 (calcd for C_20_H_27_O_6_: 363.1808). UV (CH_3_OH) λ_max_ (log ε) 208 (1.25) nm; ECD (CH_3_OH, *c* = 7.6 × 10^−5^ mol/L) λ_max_ (Δε): 258 (−0.79), 238 (+0.02), 216 (−3.22) nm. ^1^H and ^13^C NMR: see Table 5.
Compound **50**, Colorless oil; [α${]}_{D}^{28}$: +9.3 (*c* = 0.16, MeOH); FT-IR cm^−1^: 3390, 1767, 1714; MS (FAB) *m*/*z*: 379 (M + H)^+^; HRMS (FAB) *m*/*z*: 379.1749 (calcd for C_20_H_27_O_7_: 379.1757). UV (CH_3_OH) λ_max_ (log ε) 203 (1.02) nm; ECD (CH_3_OH, *c* = 6.8 × 10^−5^ mol/L) λ_max_ (Δε): 251 (−0.95), 212 (−3.26) nm. ^1^H and ^13^C NMR: see Table 5.
Compound **51**, Colorless oil; [α${]}_{D}^{17}$: +7.7 (*c* = 0.75, MeOH); FT-IR cm^−1^: 3459, 1765, 1745, 1721; MS (FAB) *m*/*z*: 501 (M + Na)^+^; HRMS (FAB) *m*/*z*: 501.1737 (calcd for C_24_H_30_O_10_Na: 501.1737). UV (CH_3_OH) λ_max_ (log ε) 204 (1.02) nm; ECD (CH_3_OH, *c* = 4.5 × 10^−5^ mol/L) λ_max_ (Δε): 255 (−1.31), 216 (−5.00) nm. ^1^H and ^13^C NMR: see Table 5.
Compound **53**, Colorless oil; [α${]}_{D}^{16}$: +32.2 (*c* = 0.07, MeOH); FT-IR cm^−1^: 3384, 1759, 1714; MS (ESI) *m*/*z*: 415 (M + Na)^+^; HRMS (ESI) *m*/*z*: 415.1368 (calcd for C_20_H_24_O_8_Na: 415.1369). ^1^H and ^13^C NMR: see Table 6.
Compound **54**, Colorless oil; [α${]}_{D}^{28}$: −62.9 (*c* = 0.63, CHCl_3_ + MeOH); FT-IR cm^−1^: 3398, 1748, 1714; MS (FAB) *m*/*z*: 415 (M + Na)^+^; HRMS (FAB) *m*/*z*: 415.1369 (calcd for C_20_H_24_O_8_Na: 415.1369). UV (CH_3_OH) λ_max_ (log ε) 203 (1.12) nm; ECD (CH_3_OH, *c* = 5.7 × 10^−5^ mol/L) λ_max_ (Δε): 258 (−1.18), 216 (−1.42) nm. ^1^H and ^13^C NMR: see Table 6.
Compound **62**, Colorless oil; [α${]}_{D}^{29}$: +7.9 (*c* = 0.03, CHCl_3_); FT-IR cm^−1^: 3501, 1769, 1715; MS (ESI) *m*/*z*: 371 (M + Na) ^+^; HRMS (ESI) *m*/*z*: 371.1472 (calcd for C_19_H_24_O_6_Na: 371.1471). UV (CH_3_OH) λ_max_ (log ε) 203 (0.62) nm; ECD (CH_3_OH, *c* = 6.3 × 10^−5^ mol/L) λ_max_ (Δε): 255 (−1.00), 210 (−1.09) nm. ^1^H and ^13^C NMR: see Table 6.

## Figures and Tables

**Table 1:** Chemical composition of samples 1–8 ^a^.

Sample No. ^b^	(*E*,*Z*)-Germacranolides and Their Oxidative Analogs	(*E*,*E*)-Germacranolides and Their Oxidative Analogs	Eudesmanolides	Guaianolides	Flavonoids	Others	Chemotype
1	**1**, **3**, **7**, **9**, **11**, **12**, **13**, **14**, **15**, **22**, **24**	**38**			**58**	**60**	Hiyodorilactone
2	**1**, **2**, **3**, **4**, **7**, **8**, **9**, **10**, **11**, **14**, **15**, **17**, **20**, **21**, **22, 24**	**37**	**51**		**57**, **58**		Hiyodorilactone
3	**1**, **3**, **4**, **18**					**60**	Hiyodorilactone
4	**1**, **2**, **3**, **4**, **5**, **7**, **16**, **17**, **19**, **20**	**40**		**53**, **54**		**61**	Hiyodorilactone
5	**1**, **2**, **3**, **4**, **7**, **9**, **11**, **14**, **16**, **17**	**38**			**57**	**60**	Hiyodorilactone
6	**1**, **3**, **4**, **5**, **6**, **7**, **9**, **11**, **12**, **13**, **14**, **15**	**37**, **38**, **39**		**56**	**57**, **58**	**61**	Hiyodorilactone
7	**3**, **23**	**25**, **26**, **27**, **28**, **29**, **30**, **31**, **32**, **34**, **35**	**42**, **47**		**57**	**61**, **63**	Eupatoriopicrin
8	**1**, **2**, **3**, **4**, **11**, **16**	**26**, **28**, **29**, **32**, **33**, **36**, **38**, **39**	**41**, **43**, **44**, **45**, **46**, **48**, **49**, **50**, **52**	**55**	**57**, **58**, **59**	**60**, **61**, **62**	Eupatoriopicrin

^a^ Red and underlines denote new compounds and major constituents, respectively. ^b^ Samples 1, 2, 7, and 8 were collected in Yunnan Province. Samples 3–6 were collected in Sichuan Province. (See Table 7 and Figure 1).

**Table 2:** ^1^H NMR data of compounds **10**, **17**, **18**, **24**, and **31** (measured at 500 MHz in CDCl_3_).

Position	10		17		18		24		31	
	δ_H_	*mult. (J* in Hz)	δ_H_	*mult. (J* in Hz)	δ_H_	*mult. (J* in Hz)	δ_H_	*mult. (J* in Hz)	δ_H_	*mult. (J* in Hz)
1	5.10	(1H, t, 7.8)	4.00	(1H, dd, 11.7, 3.4)	4.05	(1H, dd, 12.5, 3.5)	2.878	(1H, dd, 10.0, 4.2)	4.88	(1H, m)
2	2.75	(1H, m)	2.45	(1H, td, 12.7, 4.4)	2.50	(1H, td, 12.5, 4.5)	2.589	(1H, dt, 15.5, 4.6)	2.36	(1H, m)
	2.11	(1H, overlapped)	2.31	(1H, td, 12.7, 3.4)	2.29	(1H, td, 12.5, 3.5)	1.746	(1H, ddd, 15.5, 10.0, 2.4)	2.25	(1H, m)
3	5.63	(1H, dd, 11.7, 5.1)	5.75	(1H, dd, 12.7, 4.4)	5.76	(1H, dd, 12.5, 4.5)	5.262	(1H, m)	2.25	(1H, m)
									2.10	(1H, m)
5	5.24	(1H, d, 10.8)	5.34	(1H, d, 11.0)	5.35	(1H, d, 11.0)	5.289	(1H, dq, 11.3, 1.5)	4.76	(1H, d, 9.8)
6	5.31	(1H, dd, 10.8, 1.7)	5.69	(1H, dd, 11.0, 2.2)	5.66	(1H, dd, 11.0, 2.4)	6.137/6.118	(1H, dd, 11.3, 1.9)	5.12	(1H, dd, 9.8, 8.5)
7	3.00	(1H, m)	3.05	(1H, br s)	3.01	(1H, br s)	2.909	(1H, quint, 1.5)	2.92	(1H, dq, 8.5, 3.4, 3.1)
8	5.34	(1H, t, 3.0)	5.19	(1H, m)	5.19	(1H, m)	5.262/5.225	(1H, br s)	5.76	(1H, m)
9	2.78	(1H, dd, 14.2, 3.0)	2.98	(1H, dd, 15.1, 4.1)	2.98	(1H, dd, 15.2, 4.4)	2.824	(1H, dd, 15.0, 4.4)	2.79	(1H, dd, 14.4, 4.6)
	2.44	(1H, dd, 14.2, 3.0)	2.50	(1H, dd, 15.1, 2.7)	2.48	(1H, dd, 15.2, 2.9)	1.410	(1H, br d, 15.0)	2.36	(1H, m)
13	6.38	(1H, d, 2.4)	6.38	(1H, d, 2.2)	6.38	(1H, d, 2.2)	6.400	(1H, t, 2.0)	6.30	(1H, d, 3.4)
	5.80	(1H, d, 1.9)	5.83	(1H, d, 2.0)	5.79	(1H, d, 2.2)	5.813	(1H, d, 1.9)	5.59	(1H, d, 3.1)
14	1.90	(3H, s)	5.74	(1H, s)	5.80	(1H, s)	1.492	(3H, s)	1.50	(1H, s)
			5.42	(1H, s)	5.42	(1H, s)				
15	1.81	(3H, d, 1.2)	1.85	(3H, d, 1.2)	1.86	(3H, d, 1.4)	1.921	(3H, d, 1.5)	1.76	(3H, d, 1.2)
3′	6.70	(1H, dd, 2.0, 0.7)	6.95	(1H, t, 5.9)	6.83	(1H, td, 6.0, 1.2)	6.655	(1H, m)	1.66	(1H, m)
									1.44	(1H, m)
4′	7.43	(1H, t, 1.7)	4.40	(2H, d, 5.9)	4.36	(2H, dd, 6.0, 1.0)	6.177	(1H, m)	0.89	(3H, t, 7.3)
5′	8.01	(1H, dd, 1.5, 0.7)	4.35	(2H, br s)	1.82	(3H, d, 1.2)	4.885	(1H, m)	1.11	(1H, d, 7.1)
							4.690	(1H, m)		
2″	2.11	(3H, s)	2.12	(3H, s)	2.11	(3H, s)	2.163/2.160	(3H, s)		
4′-OH							3.031/2.942	(1H, d, 8.4)		

**Table 3:** ^13^C NMR data of compounds **10**, **17**, **18**, **24**, and **31** (measured at 126 MHz in CDCl_3_).

Position	10	17	18	24	31
1	124.5	83.4	83.7	60.13	130.5
2	30.6	31.5	31.4	30.52	26.2
3	70.6	69.4	69.3	72.81	39.5
4	135.9	137.3	137.0	138.92	142.5
5	125.4	126.3	126.6	125.56	127.4
6	74.2	72.6	72.2	74.48/74.46	75.6
7	48.7	48.4	48.5	48.21	52.8
8	79.1	76.7	76.2	76.90	71.2
9	43.4	38.1	38.3	43.52/43.43	44.3
10	135.9	136.4	136.1	58.00	134.7
11	137.2	136.9	137.0	136.46/136.38	136.7
12	169.3	169.7	169.1	169.21	169.6
13	124.8	125.5	125.1	125.68/125.56	121.1
14	18.6	122.5	122.7	19.55	19.2
15	18.1	17.6	17.6	23.02	17.5
1′	162.1	166.0	166.4	160.83/160.81	175.6
2′	118.5	131.3	127.7	136.21/136.10	41.6
3′	109.6	145.4	142.2	137.52	26.5
4′	144.2	58.9	59.8	103.40	11.8
5′	148.2	56.7	12.9	72.40/72.35	17.1
1″	170.2	170.6	170.2	169.17/169.12	
2″	21.2	21.1	21.1	21.20/21.18	

**Table 4:** ^1^H (400 MHz) and ^13^C (100 MHz) NMR data of compounds **36** and **40** (in CDCl_3_, at 233 K).

Position	36			40 (Major Conformer)		40 (Minor Conformer)	
	δ_H_	*mult. (J* in Hz)	δ_C_	δ_H_	*mult. (J* in Hz)	δ_H_	*mult. (J* in Hz)
1	4.13	(1H, m)	97.9	4.60	(1H, m)	4.45	(1H, m)
2	3.79	(1H, m)	66.0	2.13	(1H, m)	2.47	(1H, m)
				2.06	(1H, m)	2.13	(1H, m)
3	2.50	(1H, d, 11.2)	44.2	4.39	(1H, d, 8.3)	4.10	(1H, d, 4.4)
	2.42	(1H, d, 10.7)					
4			138.7				
5	5.37	(1H, d, 10.2)	127.5	5.41	(1H, d, 11.2)	5.44	overlaped
6	4.99	(1H, t, 10.2)	75.2	5.10	(1H, t, 10.2)	5.02	(1H, t, 12.7)
7	3.62	(1H, m)	45.7	3.55	(1H, m)	2.96	(1H, m)
8	6.04	(1H, m)	66.6	5.92	(1H, m)	5.72	(1H, d, 6.3)
9	2.77	(1H, m)	31.4	2.60	(1H, m)	3.23	(1H, m)
	1.74	(1H, dd, 11.2, 17.1)		1.81	(1H, overlaped)	2.20	(1H, m)
10			140.6				
11			134.5				
12			170.0				
13	6.29	(1H, d, 2.9)	121.2	6.30	(1H, d, 2.9)	6.33	(1H, d, 2.9)
	5.52	(1H, d, 2.9)		5.49	(1H, d, 2.9)	5.66	(1H, d, 2.9)
14	5.44	(1H, s)	120.0	5.44	(1H, s)	5.35	(1H, s)
	5.12	(1H, s)		5.13	(1H, s)	5.02	(1H, s)
15	1.97	(3H, s)	17.4	2.00	(3H, br s)	1.69	(3H, br s)
1′			166.3				
2′			131.0				
3′	6.42	(1H, q, 7.3)	141.1	6.67	(1H, t, 5.9)	6.76	(1H, t, 5.9)
4′	1.99	(3H, d, 7.3)	16.1	4.77	(2H, d, 5.9)	4.79	(2H, d, 5.9)
5′	4.20	(1H, br s)	64.1	1.86	(3H, br s)	1.89	(1H, br s)
1″							
2″				2.18	(3H, s)	2.16	(3H, s)
1-OOH				8.56	(1H, br s)	8.43	(1H, br s)

**Table 5:** ^1^H (500 MHz) and ^13^C (126 MHz) NMR data of compounds **45**, **50**, and **51** in CDCl_3_.

Position	45			50			51		
	δ_H_	*mult.* (*J* in Hz)	δ_C_	δ_H_	*mult. (J* in Hz)	δ_C_	δ_H_	*mult. (J* in Hz)	δ_C_
1	3.53	(1H, dd, 11.4, 4.9)	78.5	3.19	(1H, d, 9.0)	83.0	3.63	(1H, dd, 11.6, 4.5)	76.1
2	1.84	(1H, m)	30.8	3.62	(1H, ddd, 10.9, 9.1, 5.7)	70.6	2.24	(1H, m)	36.6
	1.59	(1H, m)					1.64	(1H, m)	
3	2.36	(1H, ddd, 14.0, 5.2, 1.9)	33.3	2.66	(1H, dd, 13.2, 5.7)	41.8	5.20	(1H, dd, 11.7, 5.6)	70.1
	2.14	(1H, td, 13.5, 5.2)		2.11	(1H, dd, 13.2, 10.9)				
4			141.7			140.1			139.5
5	2.28	(1H, d, 11.0)	53.4	2.37	(1H, d, 11.0)	53.5	2.24	(1H, m)	50.6
6	4.52	(1H, t, 11.0)	75.2	4.52	(1H, t, 11.0)	75.3	4.59	(1H, t, 11.0)	74.3
7	2.88	(1H, dq, 11.0, 3.2, 2.9)	51.9	2.90	(1H, dq, 11.0, 3.2, 2.9)	51.9	2.90	(1H, dq 11.0, 3.4, 2.9)	51.9
8	5.85	(1H, ddd, 3.6, 2.7, 2.5)	66.4	5.84	(1H, ddd, 3.7, 2.9, 2.2)	66.3	5.83	(1H, dd, 5.6, 2.6)	67.0
9	2.47	(1H, dd, 15.4, 2.5)	40.2	2.46	(1H, dd, 15.4, 2.2)	40.0	2.45	(1H, dd, 15.4, 2.6)	40.2
	1.65	(1H, dd, 15.4, 3.6)		1.63	(1H, dd, 15.4, 3.7)		1.64	(1H, m)	
10			42.6			41.9			42.4
11			169.6			134.3			133.9
12			134.5			169.9			169.4
13	6.20	(1H, d, 3.2)	119.7	6.20	(1H, d, 3.2)	119.9	6.19	(1H, d, 3.4)	120.0
	5.50	(1H, d, 2.9)		5.52	(1H, d, 2.9)		5.49	(1H, d, 2.9)	
14	0.96	(3H, s)	13.5	0.97	(3H, s)	14.5	1.00	(1H, s)	13.6
15	5.04	(1H, br s)	111.0	5.11	(1H, br s)	112.6	5.24	(1H, s)	108.5
	4.95	(1H, br s)		5.03	(1H, br s)		5.13	(1H, s)	
1′			166.0			166.2			165.7
2′			131.4			131.5			133.4
3′	6.40	(1H, q, 7.3)	141.4	6.38	(1H, q, 7.3)	140.9	6.73	(1H, t, 6.6)	138.5
4′	2.04	(3H, d, 7.3)	15.9	2.02	(3H, d, 7.3)	15.9	4.86	(2H, d, 6.6)	60.2
5′	4.26	(1H, d, 12.8)	64.7	4.22	(1H, d, 2.7)	64.2	4.38	(3H, s)	57.3
	4.20	(1H, d, 12.8)		4.17	(1H, d, 2.7)				
1″									169.8
2″							2.15	(3H, s)	21.0
1‴									170.8
2‴							2.11	(3H, s)	20.8

**Table 6:** ^1^H (500 MHz) and ^13^C (126 MHz) NMR data of compounds **53**, **54**, and **62** in CDCl_3_.

Position	53			54			62		
	δ_H_	*mult. (J* in Hz)	δ_C_	δ_H_	*mult. (J* in Hz)	δ_C_	δ_H_	*mult.* (*J* in Hz)	δ_C_
1	3.74	(1H, m)	50.8	3.58	(1H, br d, 9.8)	52.0	9.50	(1H, s)	202.8
2	2.40	(1H, dd, 14.2, 6.9)	34.7	5.77	(1H, dd, 5.8, 2.4)	133.0			
	2.11	(1H, m)							
3	4.87	(1H, d, 7.4)	94.2	6.01	(1H, dd, 5.8, 1.5)	137.2	5.10	(1H, br s)	117.3
							4.84	(1H, br s)	
4			134.7 ^a^			95.2			139.3
5			140.5 ^a^	2.96	(1H, dd, 11.3, 9.8)	49.3	2.92	(1H, d, 11.3)	51.7
6	5.25	(1H, dd, 10.5, 1.7)	75.6	4.72	(1H, dd, 11.3, 9.0)	76.8	4.62	(1H, t, 11.3)	75.4
7	3.40	(1H, ddd, 10.5, 5.0, 3.0)	47.3	3.23	(1H, dq, 9.0, 3.5, 2.9)	48.5	2.91	(1H, dd, 11.3, 2.7)	51.1
8	5.72	(1H, m)	67.9	5.71	(1H, ddd, 3.4, 3.0, 2.0)	67.9	5.87	(1H, ddd, 3.4, 2.7, 2.4)	65.8
9	2.60	(1H, dd, 13.7, 4.4)	43.2	2.81	(1H, dd, 14.5, 3.0)	43.6	2.10	(1H, dd, 15.2, 3.4)	36.4
	2.56	(1H, dd, 13.7, 4.4)		2.41	(1H, d, 14.5, 3.4)		1.88	(1H, dd, 15.2, 2.4)	
10			143.3			141.8			51.0
11			134.3			134.6			134.0
12			168.8			169.7			169.1
13	6.31	(1H, d, 3.4)	122.1	6.32	(1H, d, 3.5)	122.9	6.24	(1H, d, 3.2)	120.5
	5.62	(1H, d, 3.0)		5.66	(1H, d, 2.9)		5.57	(1H, d, 3.0)	
14	5.05	(1H, s)	114.9	4.93	(1H, br s)	117.3	1.29	(3H, s)	17.7
	4.99	(1H, s)		4.91	(1H, br s)				
15	2.00	(3H, s)	13.8	1.41	(3H, s)	20.4	1.81	(3H, s)	22.5
1′			165.8			166.0			168.9
2′			131.7			131.6			131.2
3′	6.86	(1H, t, 5.9)	143.9	6.84	(1H, t, 5.9)	144.3	6.43	(1H, q, 7.1)	141.9
4′	3.89	(2H, d, 5.9)	59.3	4.40	(2H, br d, 5.9)	59.0	2.05	(3H, d, 7.1)	15.9
5′	4.33	(2H, br s)	57.5	4.33	(1H, br s)	57.1	4.26	(1H, dd, 12.4, 4.9)	64.7
							4.23	(1H, dd, 12.4, 4.9)	
5′-OH							1.73	(1H, t, 4.9)	

^a^ Interchangeable.

**Table 7:** Collection localities of the eight *E. heterophyllum* samples.

Sample No.	Specimen No.	Location (County, Province)	Longitude/Latitude	Altitude (m)
1	2014-10	Tongdian (Lanpin, Yunnan)	99.51° E/26.72° N	2400
2	2014-48	Jinan (Lijiang, Yunnan)	100.41° E/26.85° N	2400
3	2015-14	Wolong (Wenchuan, Sichuan)	103.14° E/31.00° N	2100
4	2015-25	Jiaochang (Mao, Sichuan)	103.67° E/31.95° N	1900
5	2015-27	Hongyan (Heishui, Sichuan)	103.09° E/32.08° N	2200
6	2015-65	Xiameng (Li, Sichuan)	103.18° E/31.64° N	2000
7	2015-70	Liangwangshan (Songming, Yunnan)	102.74° E/25.26° N	2100
8	2015-71	Maanshan (Kunming, Yunnan)	102.62° E/25.09° N	2200

## Data Availability

Not applicable.

## Supplementary Materials

The following supporting information can be downloaded at: https://www.mdpi.com/article/10.3390/molecules28135107/s1, Figures S1 and S2: LC-MS chromatograms of samples 7 and 8 (under negative mode); Figures S3–S78: 1D and 2D NMR spectra of compounds **10**, **17**, **18**, **24**, **31**, **36**, **40**, **45**, **50**, **51**, **53**, **54**, and **62**; Figure S79: Experimental and calculated ECD spectra of **10**. Table S1: LC-HR-MS data for the characteristic compounds.

## Sample Availability

Samples of the compounds are not available from the authors.
