# Supplementary material for: Chemical Compositions of Eupatorium heterophyllum Leaf Samples from Yunnan and Sichuan Provinces of China—Isolation of 13 New Sesquiterpene Lactones
Source: Molecules. 2023 Jun 29;28(13):5107. doi: 10.3390/molecules28135107 (PMC10343470; doi:10.3390/molecules28135107)

# Index

**Table S1.** LC-HR-MS data for the characteristic compounds. ....1

**Figure S1.** LC-MS profiles of samples 7 (2015-70) in negative ion mode....2

**Figure S2.** LC-MS profiles of samples 8 (2015-71) in negative ion mode....4

**Figure S3.** $^{1}H$ NMR spectrum of **10** (measured in $CDCl_3$, 500 MHz)....6

**Figure S4.** $^{13}C$ NMR spectrum of **10** (measured in $CDCl_3$, 126 MHz). ....7

**Figure S5.** $^{1}H$-$^{1}H$ COSY spectrum of **10** (measured in $CDCl_3$, 500 MHz)....8

**Figure S6.** HSQC spectrum of **10** (measured in $CDCl_3$, 500 MHz). ....9

**Figure S7.** HMBC spectrum of **10** (measured in $CDCl_3$, 500 MHz)....10

**Figure S8.** NOESY spectrum of **10** (measured in $CDCl_3$, 500 MHz). ....11

**Figure S9.** $^{1}H$ NMR spectrum of **17** (measured in $CDCl_3$, 500 MHz)....12

**Figure S10.** $^{13}C$ NMR spectrum of **17** (measured in $CDCl_3$, 126 MHz). ....13

**Figure S11.** $^{1}H$-$^{1}H$ COSY spectrum of **17** (measured in $CDCl_3$, 500 MHz)....14

**Figure S12.** HSQC spectrum of **17** (measured in $CDCl_3$, 500 MHz)....15

**Figure S13.** HMBC spectrum of **17** (measured in $CDCl_3$, 500 MHz). ....16

**Figure S14.** NOESY spectrum of **17** (measured in $CDCl_3$, 500 MHz)....17

**Figure S15.** $^{1}H$ NMR spectrum of **18** (measured in $CDCl_3$, 500 MHz). ....18

**Figure S16.** $^{13}C$ NMR spectrum of **18** (measured in $CDCl_3$, 126 MHz). ....19

**Figure S17.** ¹H-¹H COSY spectrum of **18** (measured in $CDCl_3$, 500 MHz). ........ 20

**Figure S18.** HSQC spectrum of **18** (measured in $CDCl_3$, 500 MHz)........ 21

**Figure S19.** HMBC spectrum of **18** (measured in $CDCl_3$, 500 MHz). ........ 22

**Figure S20.** NOESY spectrum of **18** (measured in $CDCl_3$, 500 MHz)........ 23

**Figure S21.** $^1H$ NMR spectrum of **24** (measured in $CDCl_3$, 500 MHz). ........ 24

**Figure S22.** $^{13}C$ NMR spectrum of **24** (measured in $CDCl_3$, 126 MHz). ........ 25

**Figure S23.** $^1H$-$^1H$ COSY spectrum of **24** (measured in $CDCl_3$, 500 MHz). ........ 26

**Figure S24.** HSQC spectrum of **24** (measured in $CDCl_3$, 500 MHz)........ 27

**Figure S25.** HMBC spectrum of **24** (measured in $CDCl_3$, 500 MHz). ........ 28

**Figure S26.** NOESY spectrum of **24** (measured in $CDCl_3$, 500 MHz)........ 29

**Figure S27.** $^1H$ NMR spectrum of **31** (measured in $CDCl_3$, 500 MHz). ........ 30

**Figure S28.** $^{13}C$ NMR spectrum of **31** (measured in $CDCl_3$, 126 MHz). ........ 31

**Figure S29.** $^1H$-$^1H$ COSY spectrum of **31** (measured in $CDCl_3$, 500 MHz). ........ 32

**Figure S30.** HSQC spectrum of **31** (measured in $CDCl_3$, 500 MHz)........ 33

**Figure S31.** HMBC spectrum of **31** (measured in $CDCl_3$, 500 MHz). ........ 34

**Figure S32.** NOESY spectrum of **31** (measured in $CDCl_3$, 500 MHz)........ 35

**Figure S33.** $^1H$ NMR spectrum of **36** (measured in $CDCl_3$, 500 MHz, 298 K)........ 36

**Figure S34.** $^1H$ NMR spectrum of **36** (measured in $CDCl_3$, 400 MHz, 233 K)........ 37

**Figure S35.** $^{13}C$ NMR spectrum of **36** (measured in $CDCl_3$, 100 MHz, 233 K). .................................. 38

**Figure S36.** $^{1}H$-$^{1}H$ COSY spectrum of **36** (measured in $CDCl_3$, 400 MHz, 233 K)............................. 39

**Figure S37.** HSQC spectrum of **36** (measured in $CDCl_3$, 400 MHz, 233 K)......................................... 40

**Figure S38.** HMBC spectrum of **36** (measured in $CDCl_3$, 400 MHz, 233 K)......................................... 41

**Figure S39.** NOESY spectrum of **36** (measured in $CDCl_3$, 400 MHz, 233 K)...................................... 42

**Figure S40.** $^{1}H$ NMR spectrum of **40** (measured in $CDCl_3$, 500 MHz, 298 K)..................................... 43

**Figure S41.** $^{1}H$ NMR spectrum of **40** (measured in $CDCl_3$, 400 MHz, 233 K)..................................... 44

**Figure S42.** $^{1}H$-$^{1}H$ COSY spectrum of **40** (measured in $CDCl_3$, 400 MHz, 233 K)............................. 45

**Figure S43.** $^{1}H$ NMR spectrum of **45** (measured in $CDCl_3$, 500 MHz). .................................................. 46

**Figure S44.** $^{13}C$ NMR spectrum of **45** (measured in $CDCl_3$, 126 MHz)................................................. 47

**Figure S45.** $^{1}H$-$^{1}H$ COSY spectrum of **45** (measured in $CDCl_3$, 500 MHz). ......................................... 48

**Figure S46.** HSQC spectrum of **45** (measured in $CDCl_3$, 500 MHz)....................................................... 49

**Figure S47.** HMBC spectrum of **45** (measured in $CDCl_3$, 500 MHz). .................................................... 50

**Figure S48.** NOESY spectrum of **45** (measured in $CDCl_3$, 500 MHz)................................................... 51

**Figure S49.** $^{1}H$ NMR spectrum of **50** (measured in $CDCl_3$, 500 MHz). .................................................. 52

**Figure S50.** $^{13}C$ NMR spectrum of **50** (measured in $CDCl_3$, 126 MHz)................................................. 53

**Figure S51.** $^{1}H$-$^{1}H$ COSY spectrum of **50** (measured in $CDCl_3$, 500 MHz). ......................................... 54

**Figure S52.** HSQC spectrum of **50** (measured in $CDCl_3$, 500 MHz)....................................................... 55

**Figure S53.** HMBC spectrum of **50** (measured in $CDCl_3$, 500 MHz). .................................................... 56

**Figure S54.** NOESY spectrum of **50** (measured in $CDCl_3$, 500 MHz).................................................... 57

**Figure S55.** $^1H$ NMR spectrum of **51** (measured in $CDCl_3$, 500 MHz). ................................................. 58

**Figure S56.** $^{13}C$ NMR spectrum of **51** (measured in $CDCl_3$, 126 MHz). ................................................ 59

**Figure S57.** $^1H$-$^1H$ COSY spectrum of **51** (measured in $CDCl_3$, 500 MHz). ......................................... 60

**Figure S58.** HSQC spectrum of **51** (measured in $CDCl_3$, 500 MHz)....................................................... 61

**Figure S59.** HMBC spectrum of **51** (measured in $CDCl_3$, 500 MHz). .................................................... 62

**Figure S60.** NOESY spectrum of **51** (measured in $CDCl_3$, 500 MHz).................................................... 63

**Figure S61.** $^1H$ NMR spectrum of **53** (measured in $CDCl_3$, 500 MHz). ................................................. 64

**Figure S62.** $^{13}C$ NMR spectrum of **53** (measured in $CDCl_3$, 126 MHz). ................................................ 65

**Figure S63.** $^1H$-$^1H$ COSY spectrum of **53** (measured in $CDCl_3$, 500 MHz). ......................................... 66

**Figure S64.** HSQC spectrum of **53** (measured in $CDCl_3$, 500 MHz)....................................................... 67

**Figure S65.** HMBC spectrum of **53** (measured in $CDCl_3$, 500 MHz). .................................................... 68

**Figure S66.** NOESY spectrum of **53** (measured in $CDCl_3$, 500 MHz).................................................... 69

**Figure S67.** $^1H$ NMR spectrum of **54** (measured in $CDCl_3$, 500 MHz). ................................................. 70

**Figure S68.** $^{13}C$ NMR spectrum of **54** (measured in $CDCl_3$, 126 MHz). ................................................ 71

**Figure S69.** $^1H$-$^1H$ COSY spectrum of **54** (measured in $CDCl_3$, 500 MHz). ......................................... 72

**Figure S70.** HSQC spectrum of **54** (measured in $CDCl_3$, 500 MHz)....................................................... 73

**Figure S71.** HMBC spectrum of **54** (measured in $CDCl_3$, 500 MHz). .................................................... 74

**Figure S72.** NOESY spectrum of **54** (measured in $CDCl_3$, 500 MHz)................................................... 75

**Figure S73.** $^1$H NMR spectrum of **62** (measured in $CDCl_3$, 500 MHz). ................................................ 76

**Figure S74.** $^{13}$C NMR spectrum of **62** (measured in $CDCl_3$, 126 MHz). ............................................... 77

**Figure S75.** $^1$H-$^1$H COSY spectrum of **62** (measured in $CDCl_3$, 500 MHz). ......................................... 78

**Figure S76.** HSQC spectrum of **62** (measured in $CDCl_3$, 500 MHz)...................................................... 79

**Figure S77.** HMBC spectrum of **62** (measured in $CDCl_3$, 500 MHz). .................................................... 80

**Figure S78.** NOESY spectrum of **62** (measured in $CDCl_3$, 500 MHz)................................................... 81

**Figure S79**. Experimental and calculated ECD spectra of **10**. ...........................................82

**Table S1.** LC-HR-MS data for the characteristic compounds.

| Compound | $t_R$ (min) | Ions mode | Formula | Detected | Calculated | Error (mDa) |
|---|---|---|---|---|---|---|
| **1** | 8.56 | $[M+Na]^+$ | $C_{22}H_{28}O_8$ | 443.1686 | 443.1682 | 0.4 |
| **2** | 9.14 | $[M+Na]^+$ | $C_{22}H_{28}O_9$ | 443.1685 | 443.1682 | 0.3 |
| **3** | 9.65 | $[M+Na]^+$ | $C_{22}H_{28}O_7$ | 427.1732 | 427.1733 | -0.1 |
| **4** | 10.02 | $[M+Na]^+$ | $C_{22}H_{28}O_7$ | 427.1735 | 427.1733 | 0.2 |
| **11** | 9.02 | $[M+Na]^+$ | $C_{22}H_{26}O_8$ | 441.1532 | 441.1525 | 0.7 |
| **16** | 6.11 | $[M+Na]^+$ | $C_{22}H_{28}O_{10}$ | 475.1572 | 475.1580 | -0.8 |
| **25** | 10.62 | $[M+Na]^+$ | $C_{22}H_{28}O_7$ | 427.1735 | 427.1733 | 0.2 |
| **26** | 9.51 | $[M+Na]^+$ | $C_{20}H_{26}O_6$ | 385.1627 | 385.1627 | 0.0 |
| **27** | 10.88 | $[M+Na]^+$ | $C_{20}H_{26}O_5$ | 369.1687 | 369.1678 | 0.9 |
| **28** | 10.73 | $[M+Na]^+$ | $C_{20}H_{26}O_5$ | 369.1682 | 369.1678 | 0.4 |
| **29** | 10.65 | $[M+Na]^+$ | $C_{20}H_{26}O_5$ | 369.1673 | 369.1678 | -0.5 |
| **30** | 12.67 | $[M+Na]^+$ | $C_{20}H_{26}O_4$ | 353.1736 | 353.1729 | 0.7 |
| **37** | 9.71 | $[M+Na]^+$ | $C_{24}H_{30}O_9$ | 485.1796 | 485.1788 | 0.8 |
| **38** | 7.93 | $[M+Na]^+$ | $C_{22}H_{28}O_8$ | 443.1685 | 443.1682 | 0.3 |
| **44** | 6.48 | $[M+Na]^+$ | $C_{20}H_{26}O_7$ | 401.1581 | 401.1576 | 0.5 |
| **55** | 8.70 | $[M+Na]^+$ | $C_{22}H_{26}O_8$ | 441.1524 | 441.1525 | -0.1 |
| **57** | 8.94 | $[M+Na]^+$ | $C_{16}H_{12}O_7$ | 339.0475 | 339.0481 | -0.6 |
| **59** | 6.77 | $[M+Na]^+$ | $C_{21}H_{20}O_{12}$ | 487.085 | 487.0852 | -0.2 |
| **57** | 8.94 | $[M-H]^-$ | $C_{16}H_{12}O_7$ | 315.0505 | 315.0505 | 0.0 |
| **59** | 6.77 | $[M-H]^-$ | $C_{21}H_{20}O_{12}$ | 463.0877 | 463.0877 | 0.0 |

**Figure S1.** LC-MS profiles of sample 7 (2015-70) in negative ion mode for detecting flavonoidal constituents, **57** and **59**. A: total ion chromatogram; B: base peak ion chromatogram; C: extracted ion chromatogram of *m/z* 463.09; D: extracted ion chromatogram of *m/z* 315.05.

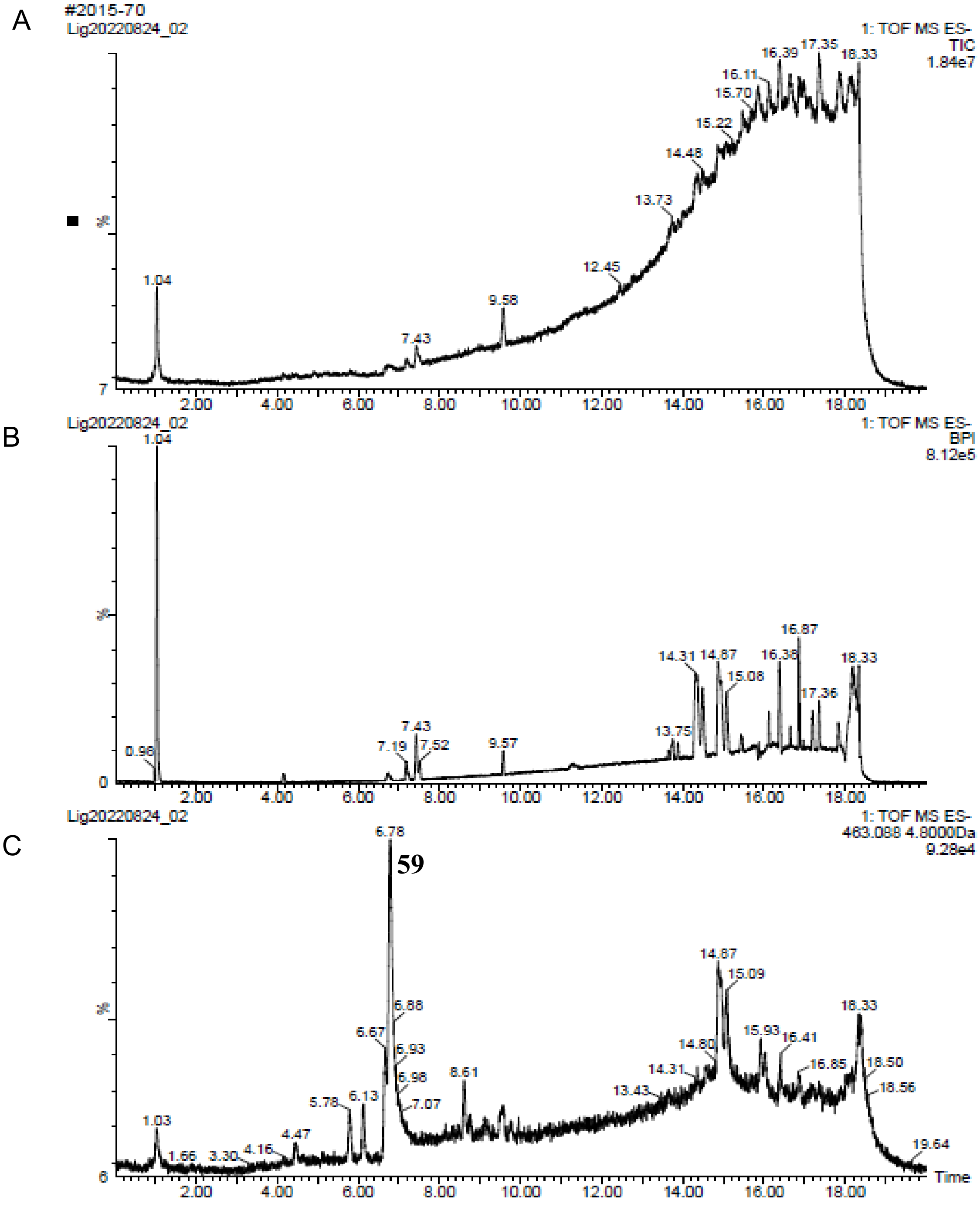

D

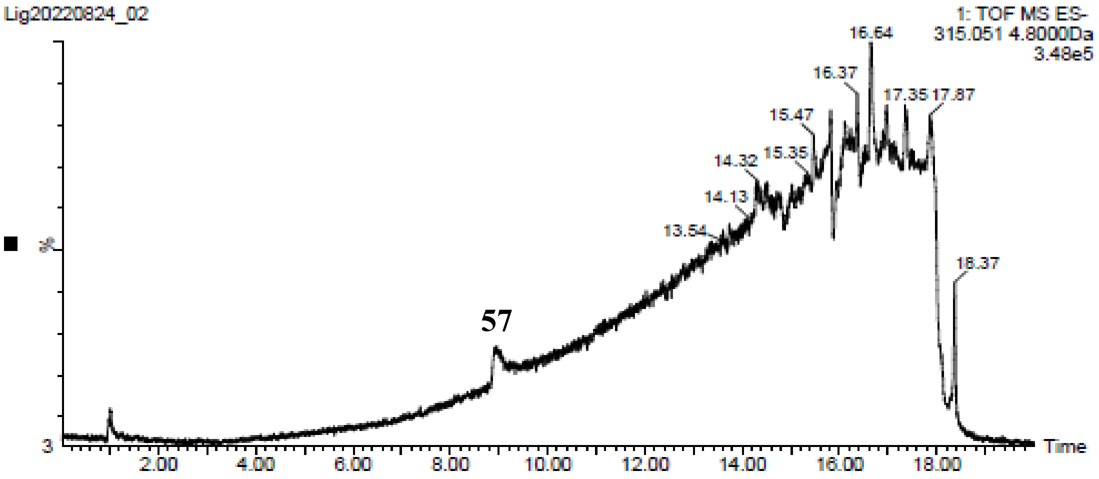

Lig20220824_02
1: TOF MS ES-
315.051 4.8000Da
3.48e5
%
57
13.54
14.13
14.32
15.35
15.47
16.37
16.64
17.35
17.87
18.37
3
2.00
4.00
6.00
8.00
10.00
12.00
14.00
16.00
18.00
Time

**Figure S2.** LC-MS profiles of sample 8 (2015-71) in negative ion mode for detecting flavonoidal constituents, **57** and **59**. A: total ion chromatogram; B: base peak ion chromatogram; C: extracted ion chromatogram of *m/z* 315.05; D: extracted ion chromatogram of *m/z* 463.09.

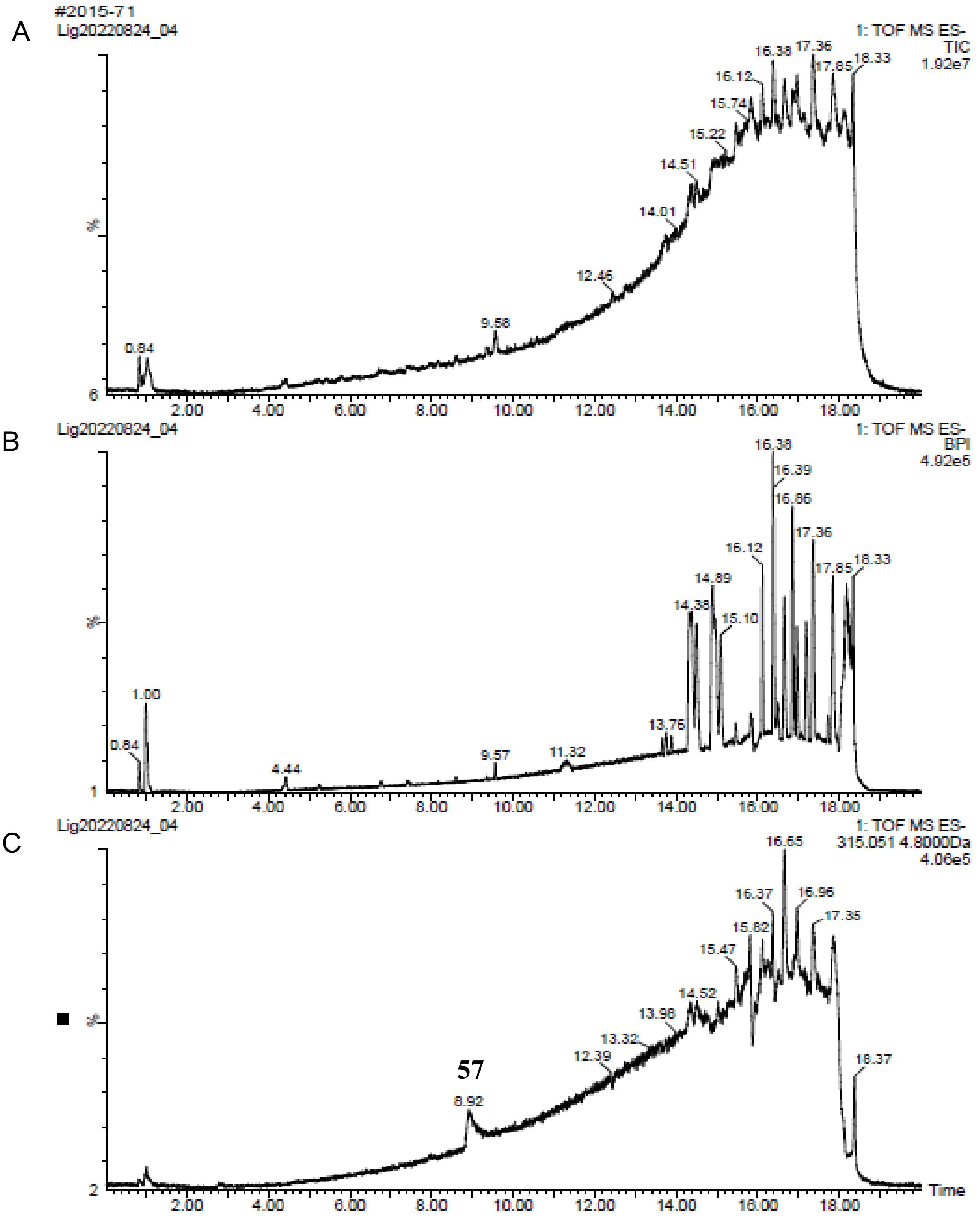

D

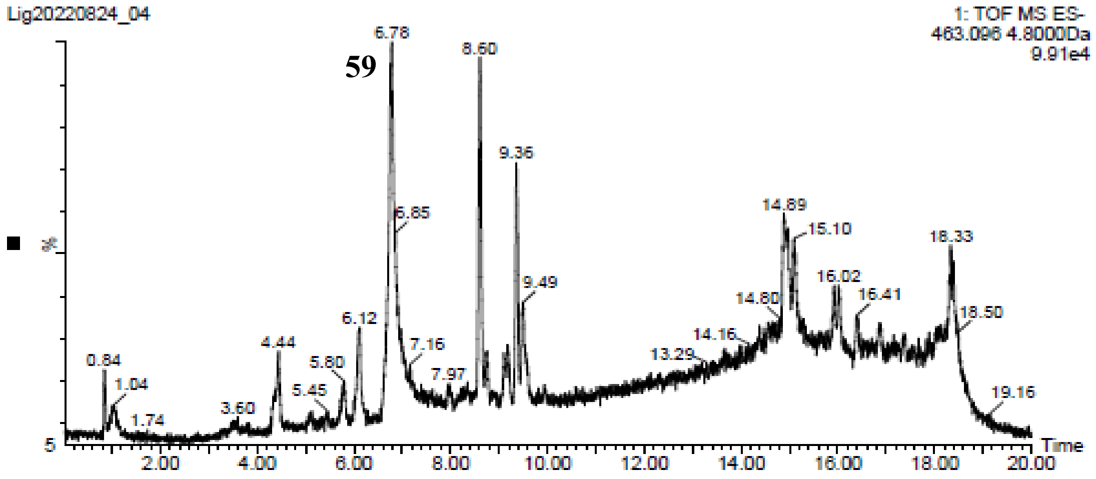
Lig20220824_04
1: TOF MS ES-
463.096 4.8000Da
9.91e4
59
6.78
8.60
9.36
6.85
14.89
15.10
18.33
9.49
16.02
16.41
14.80
18.50
6.12
14.16
7.16
4.44
13.29
5.80
0.84
7.97
1.04
5.45
19.16
3.60
1.74
%
5
Time
2.00
4.00
6.00
8.00
10.00
12.00
14.00
16.00
18.00
20.00

**Figure S3.** $^{1}$H NMR spectrum of **10** (measured in $CDCl_3$, 500 MHz).

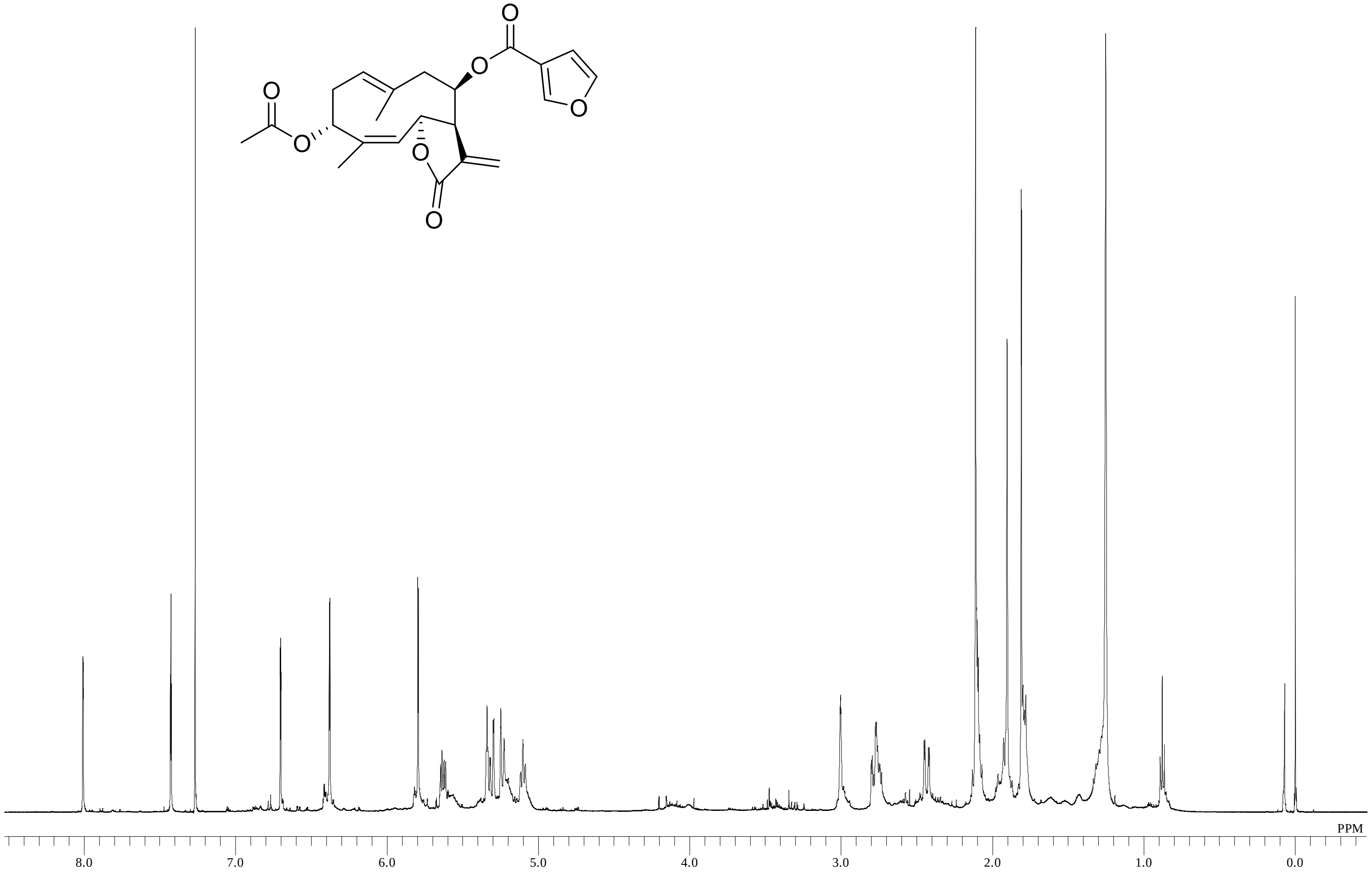

**Figure S4.** $^{13}$C NMR spectrum of **10** (measured in $CDCl_3$, 126 MHz).

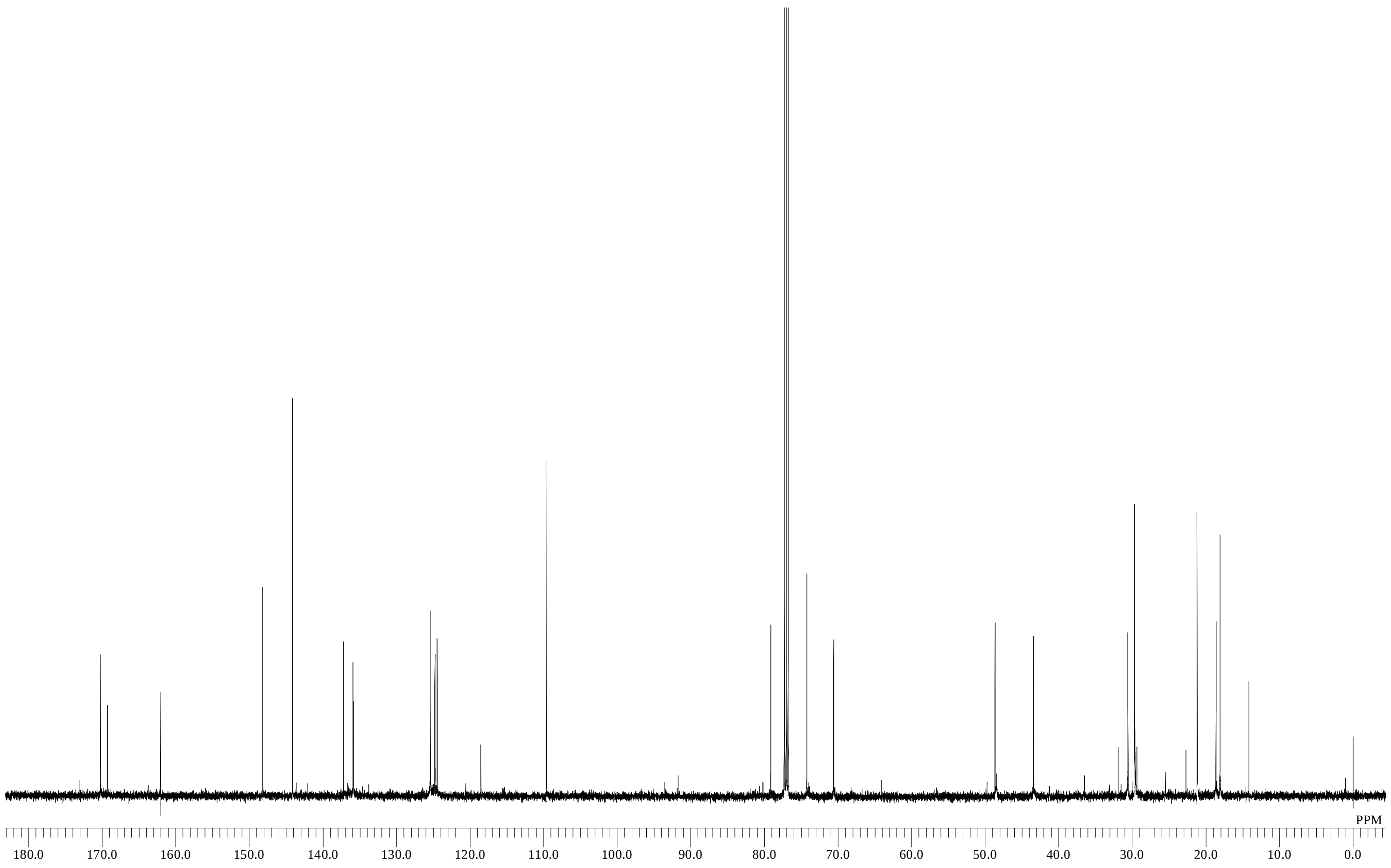

**Figure S5.** $^{1}H$-$^{1}H$ COSY spectrum of **10** (measured in $CDCl_3$, 500 MHz).

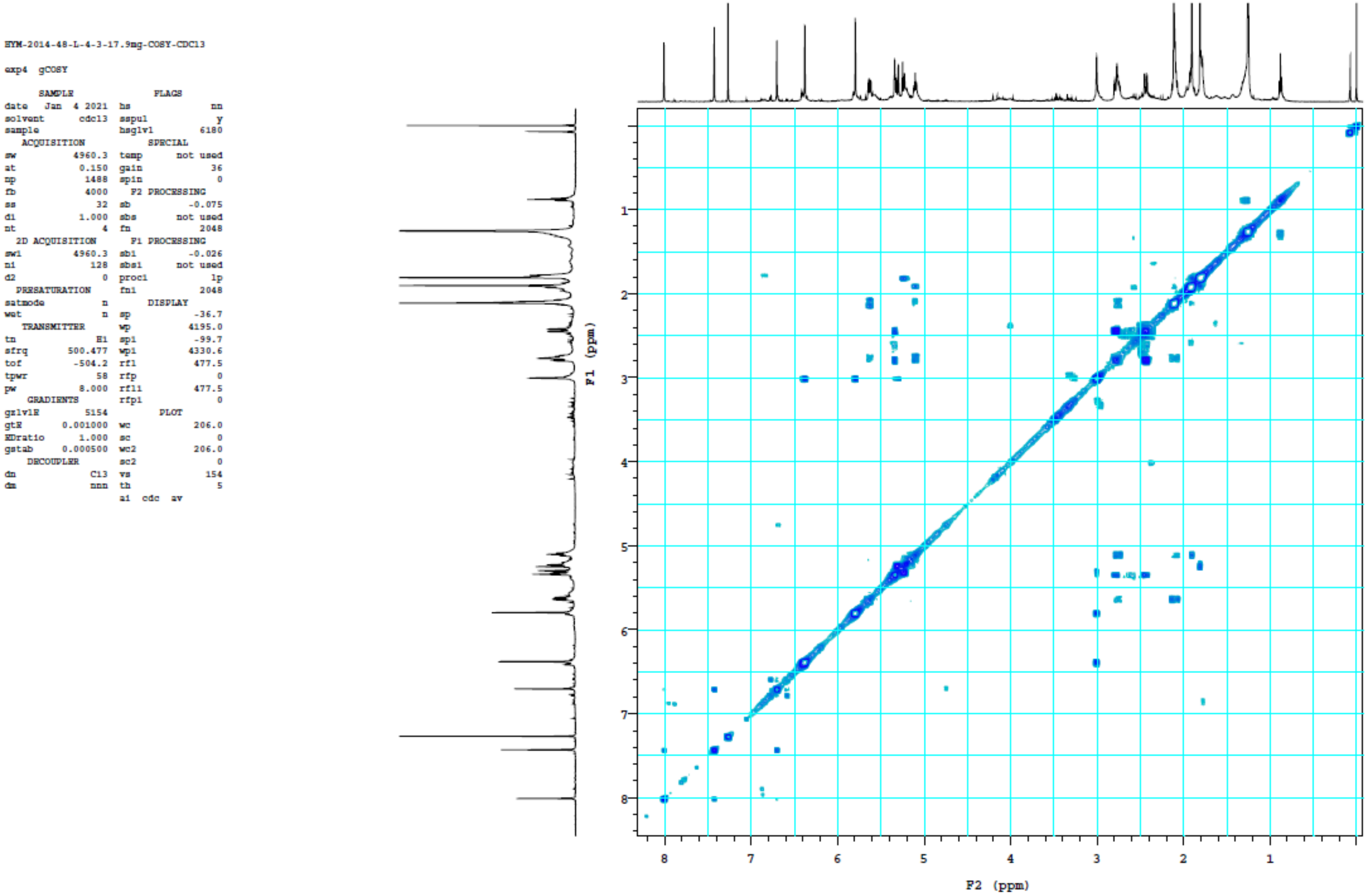

**Figure S6.** HSQC spectrum of **10** (measured in $CDCl_3$, 500 MHz).

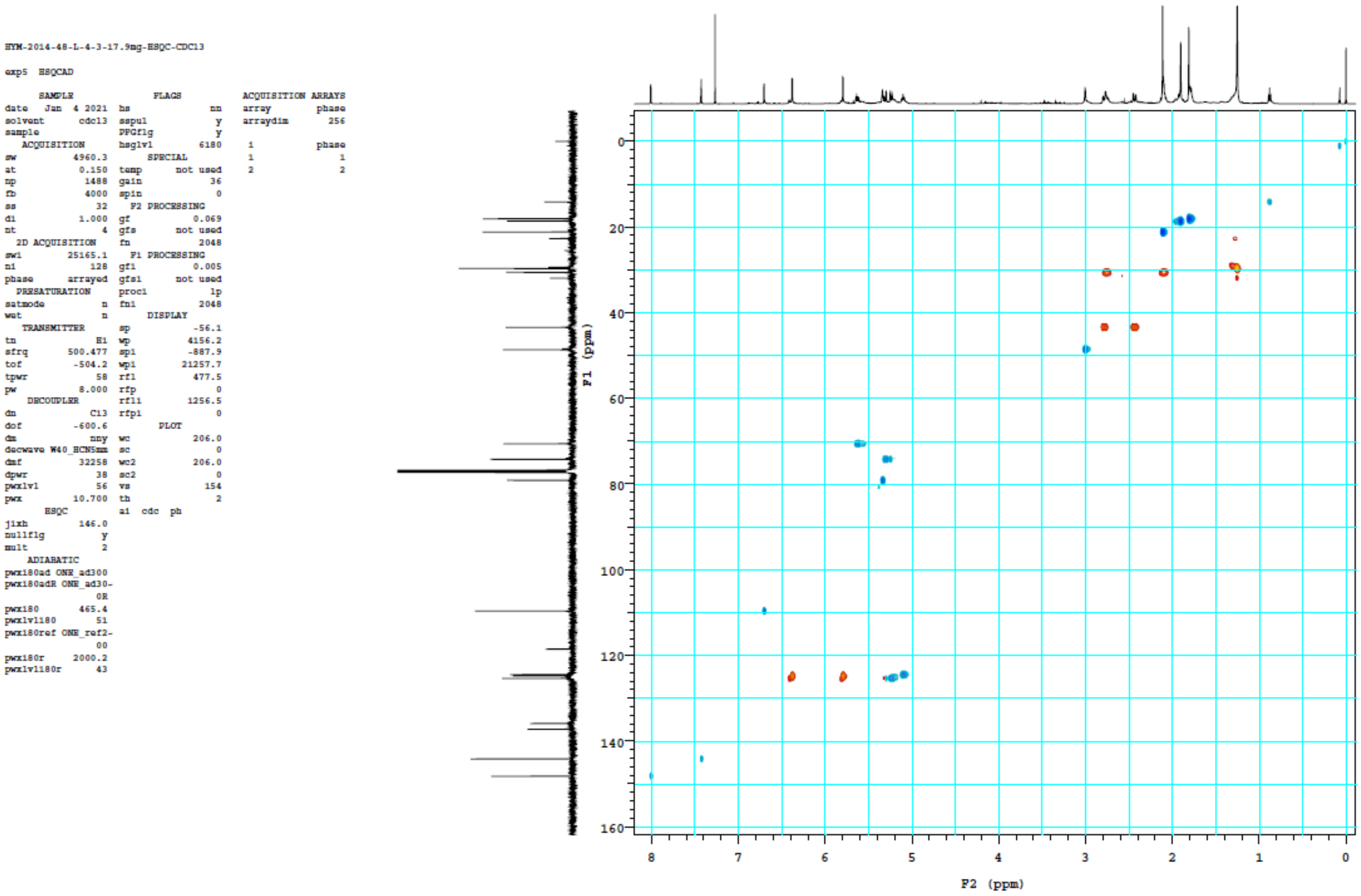

**Figure S7.** HMBC spectrum of **10** (measured in $CDCl_3$, 500 MHz).

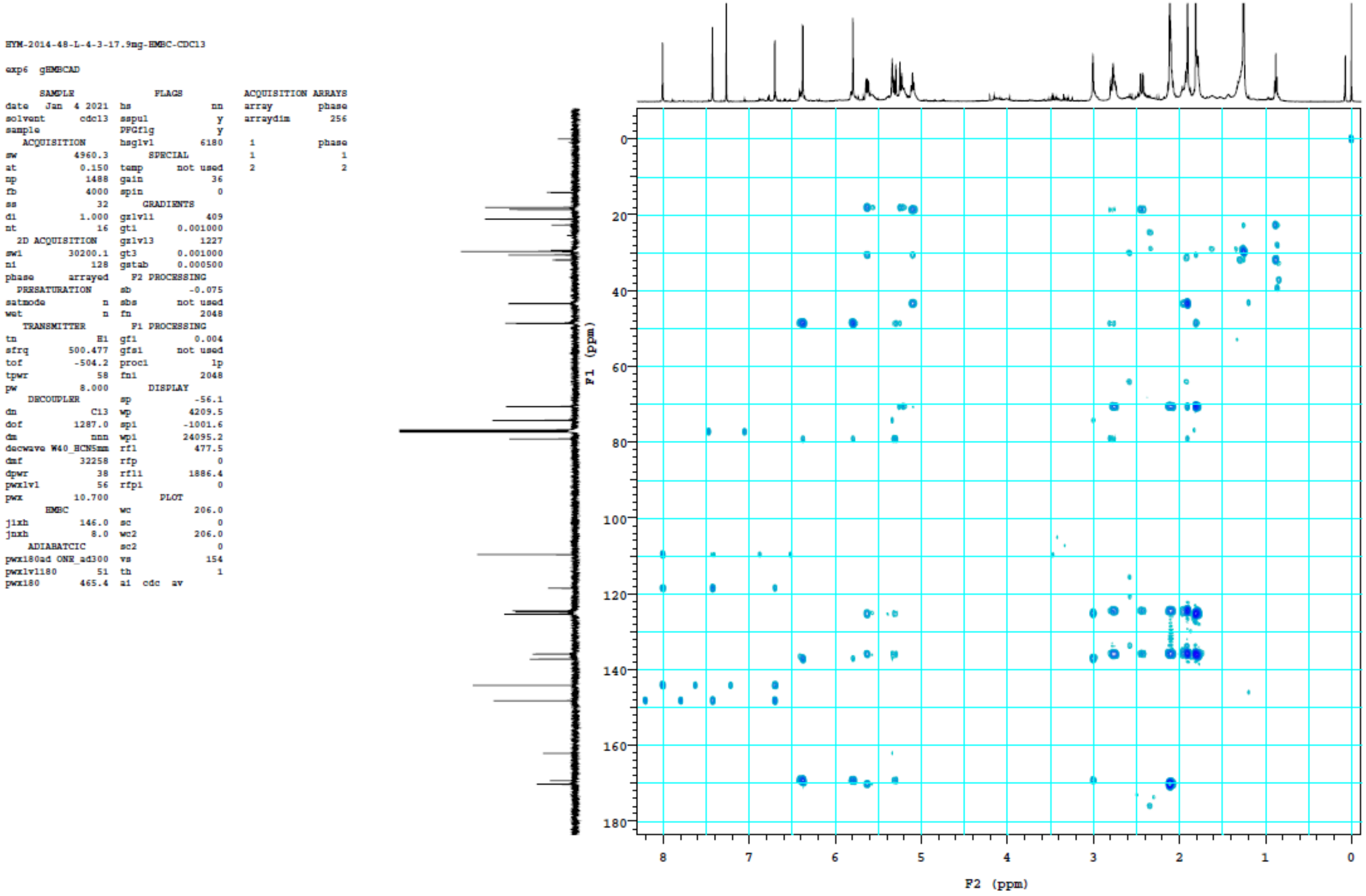

**Figure S8.** NOESY spectrum of **10** (measured in $CDCl_3$, 500 MHz).

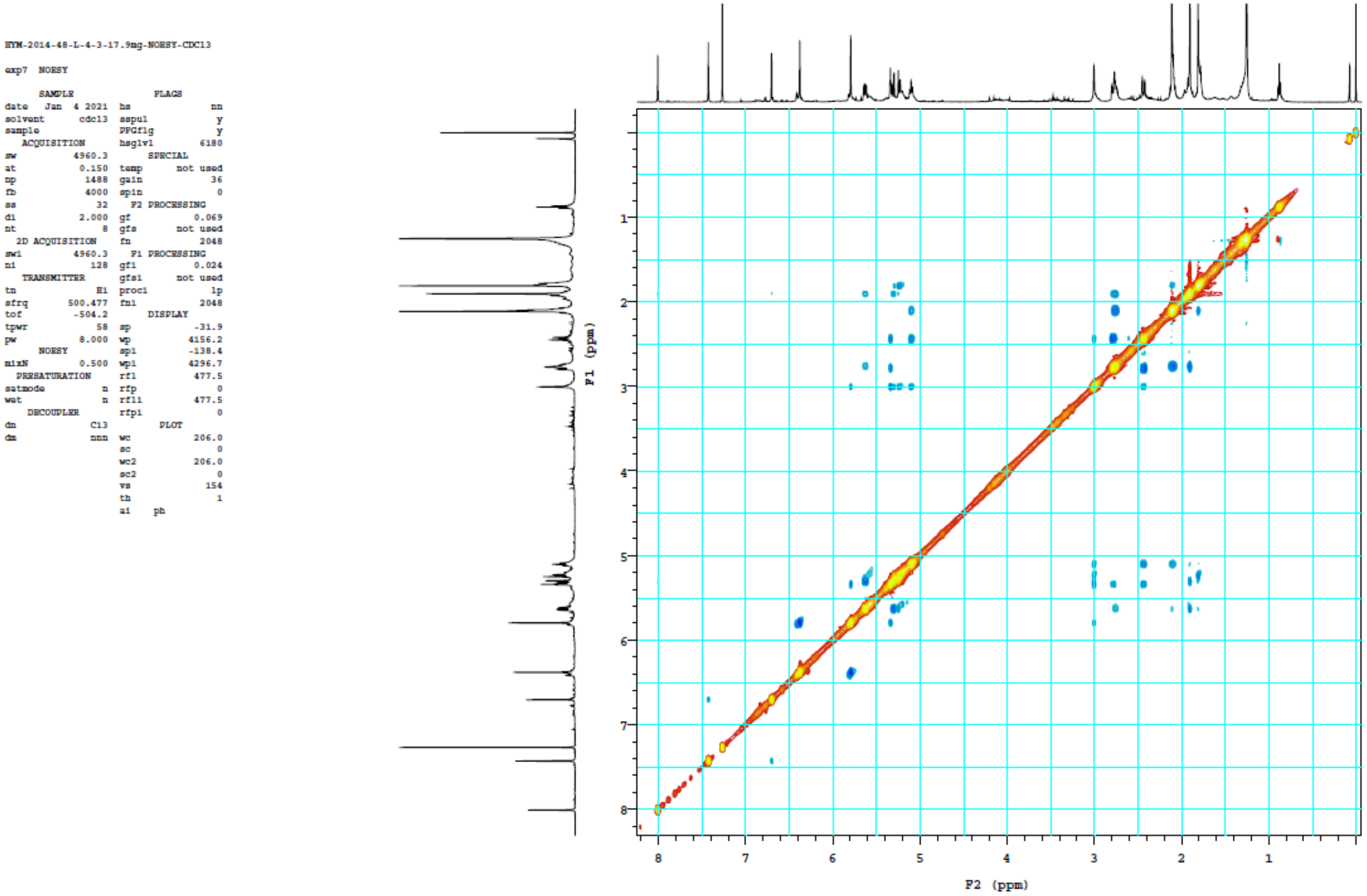

**Figure S9.** $^{1}$H NMR spectrum of **17** (measured in $CDCl_3$, 500 MHz).

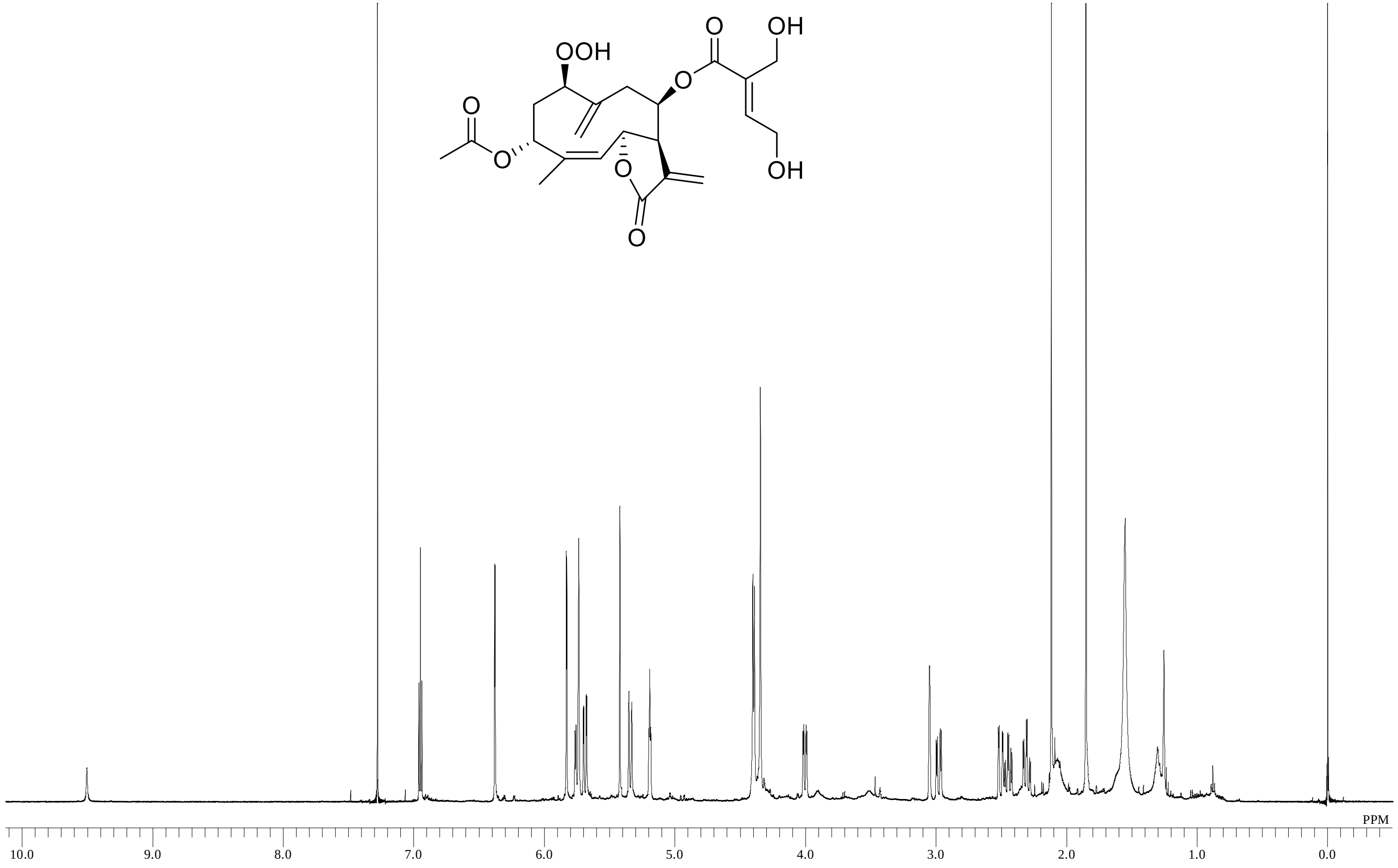

**Figure S10.** $^{13}C$ NMR spectrum of **17** (measured in $CDCl_3$, 126 MHz).

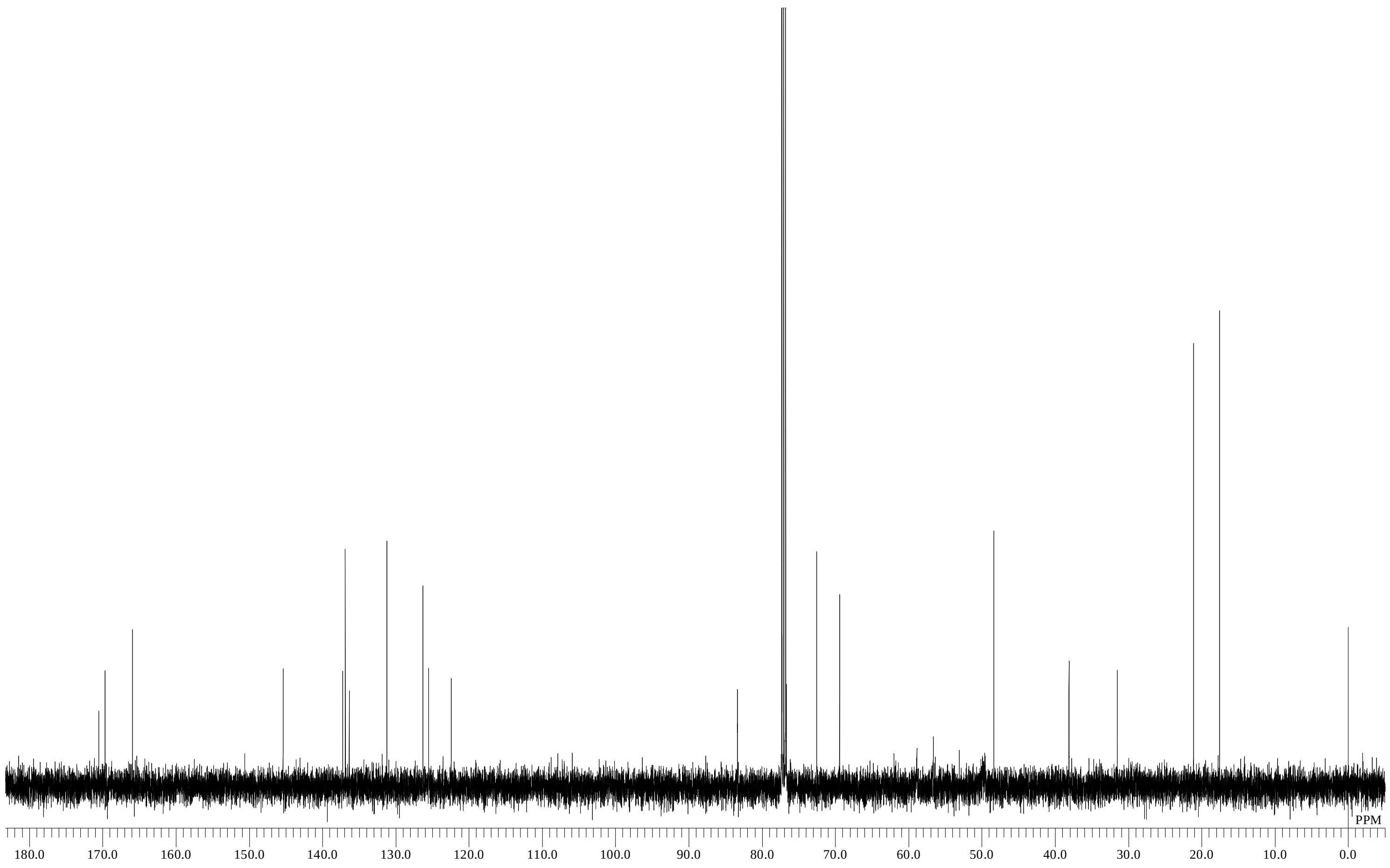

**Figure S11.** $^1$H-$^1$H COSY spectrum of **17** (measured in $CDCl_3$, 500 MHz).

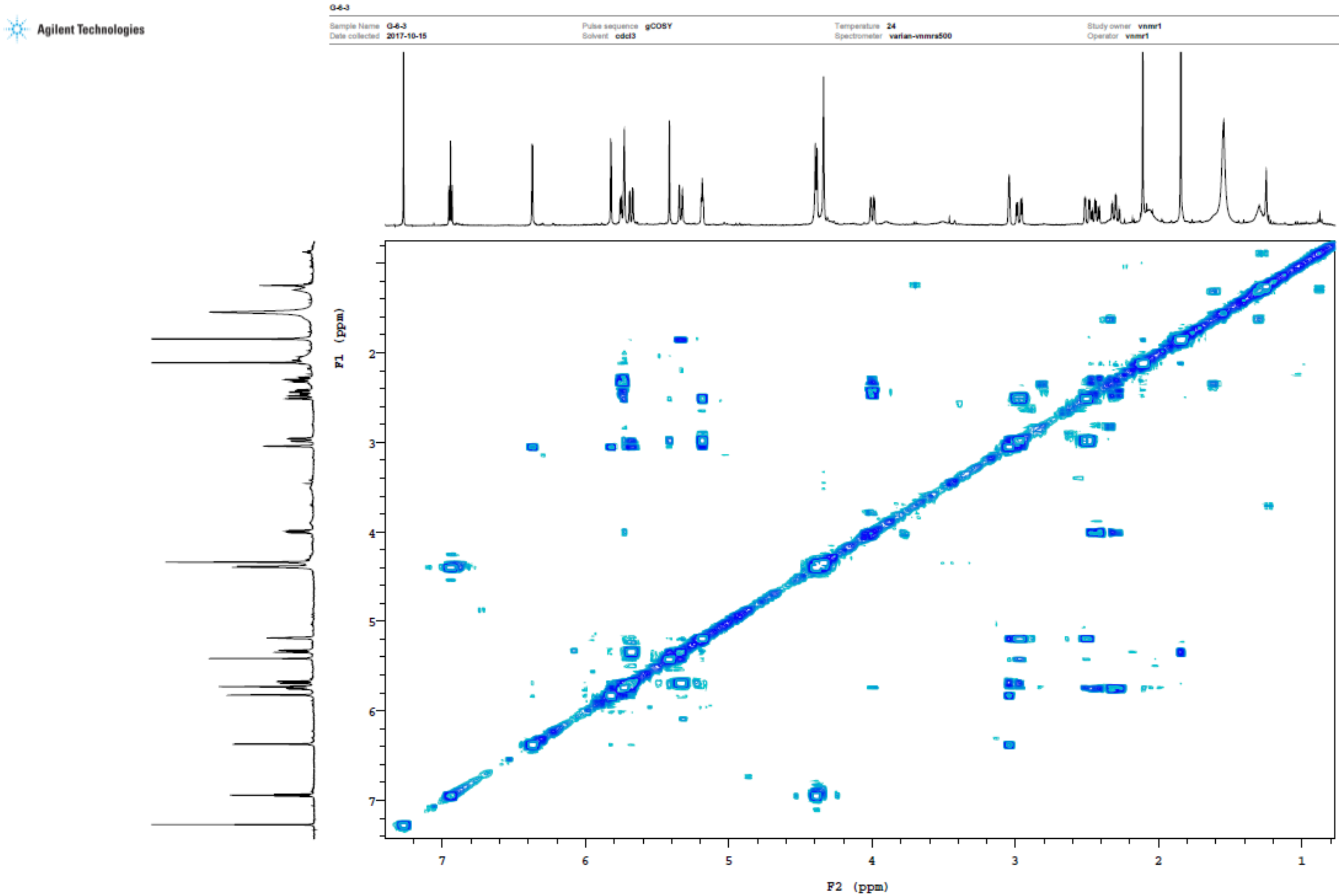

**Figure S12.** HSQC spectrum of **17** (measured in $CDCl_3$, 500 MHz).

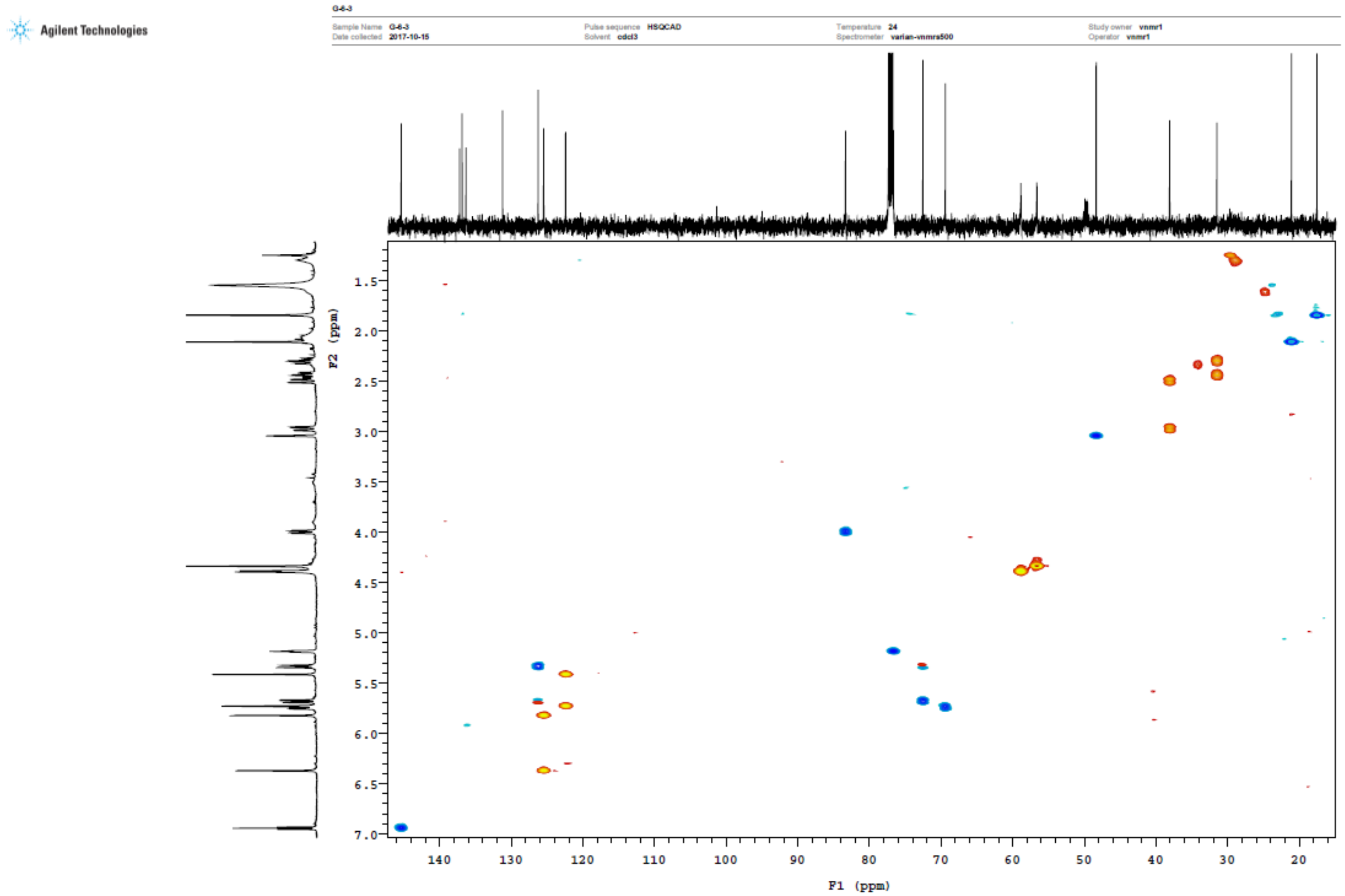

**Figure S13.** HMBC spectrum of **17** (measured in $CDCl_3$, 500 MHz).

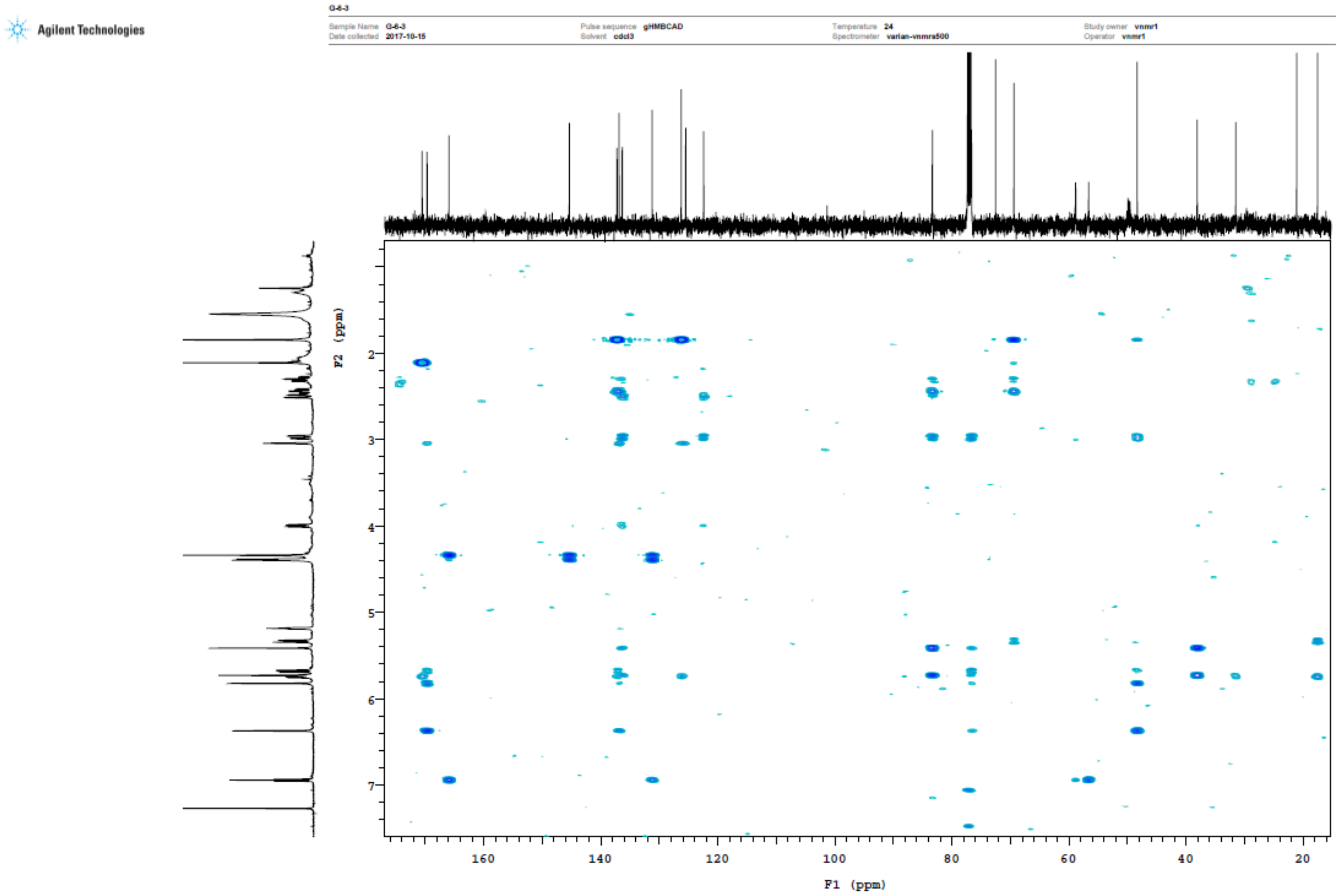

**Figure S14.** NOESY spectrum of **17** (measured in $CDCl_3$, 500 MHz).

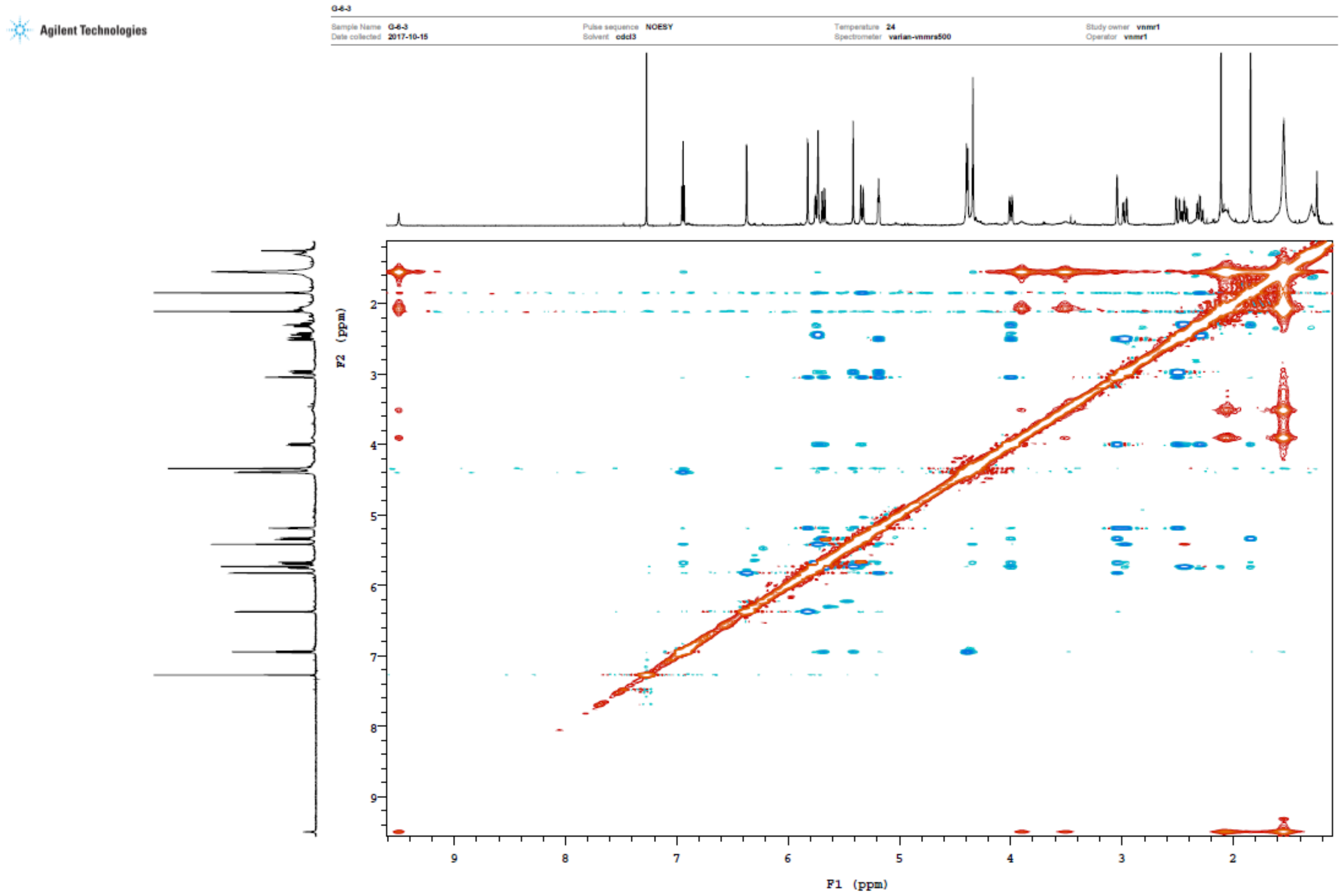

**Figure S15.** $^{1}$H NMR spectrum of **18** (measured in $CDCl_3$, 500 MHz).

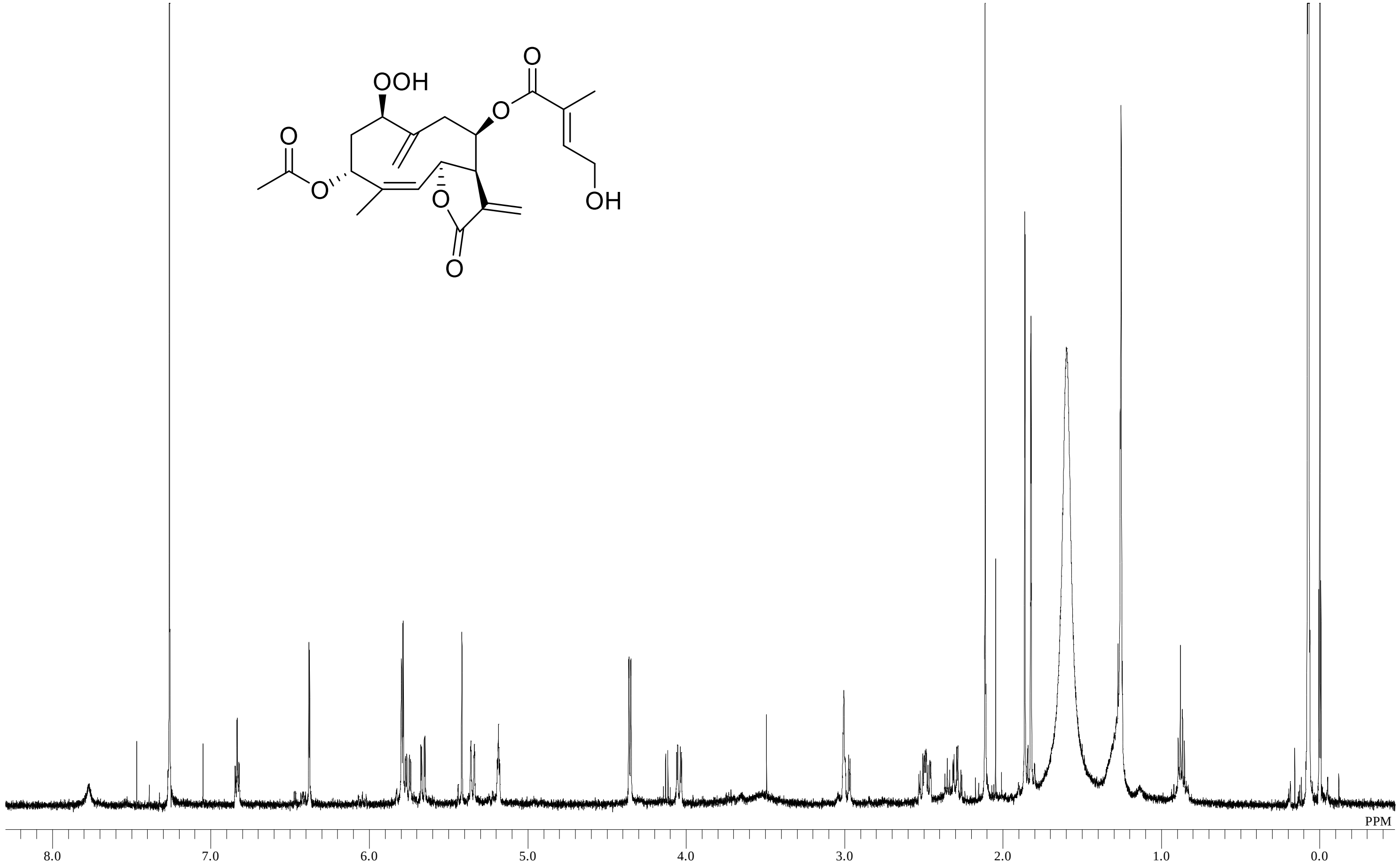

**Figure S16.** $^{13}$C NMR spectrum of **18** (measured in $CDCl_3$, 126 MHz).

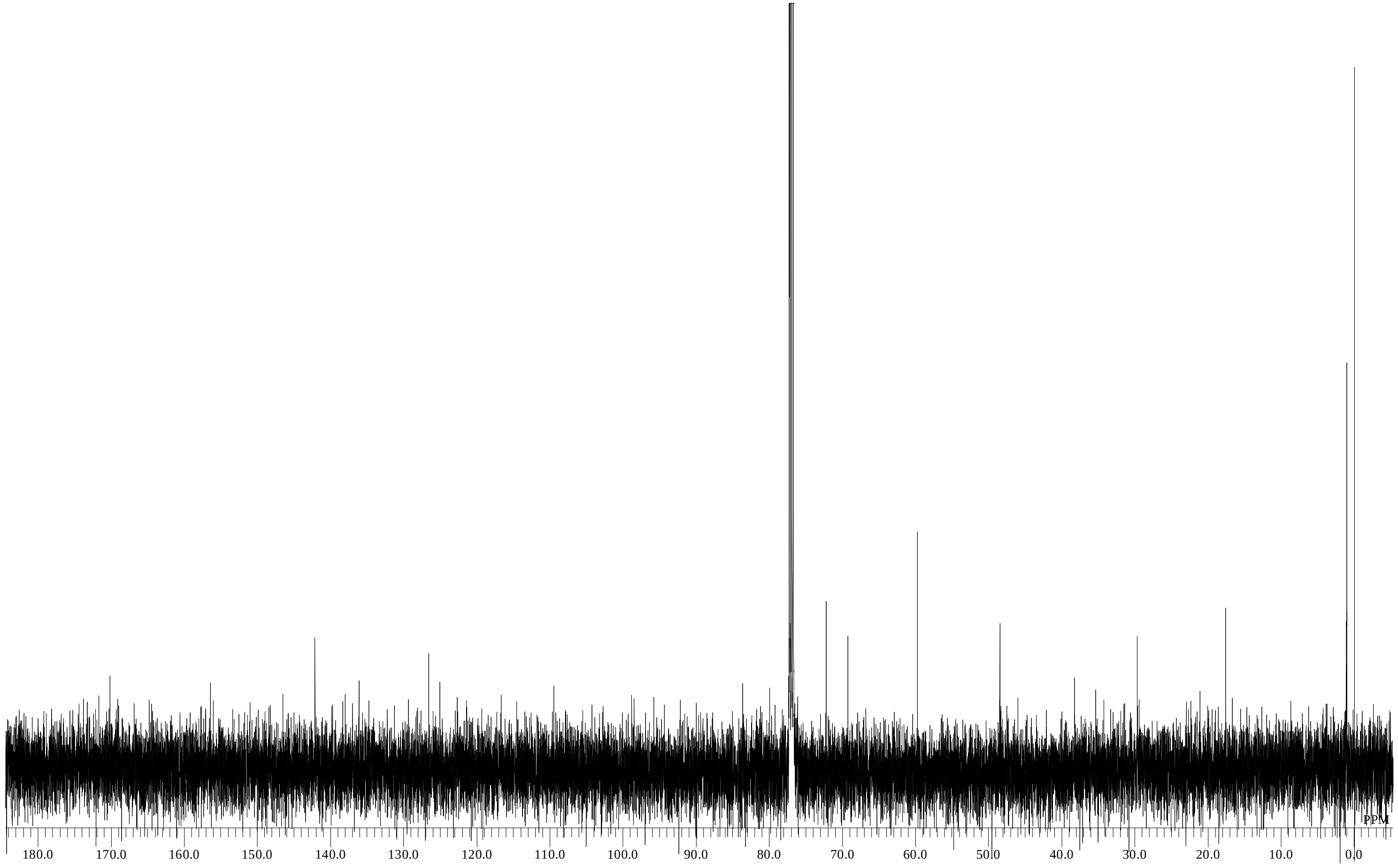

**Figure S17.** $^{1}$H-$^{1}$H COSY spectrum of **18** (measured in $CDCl_3$, 500 MHz).

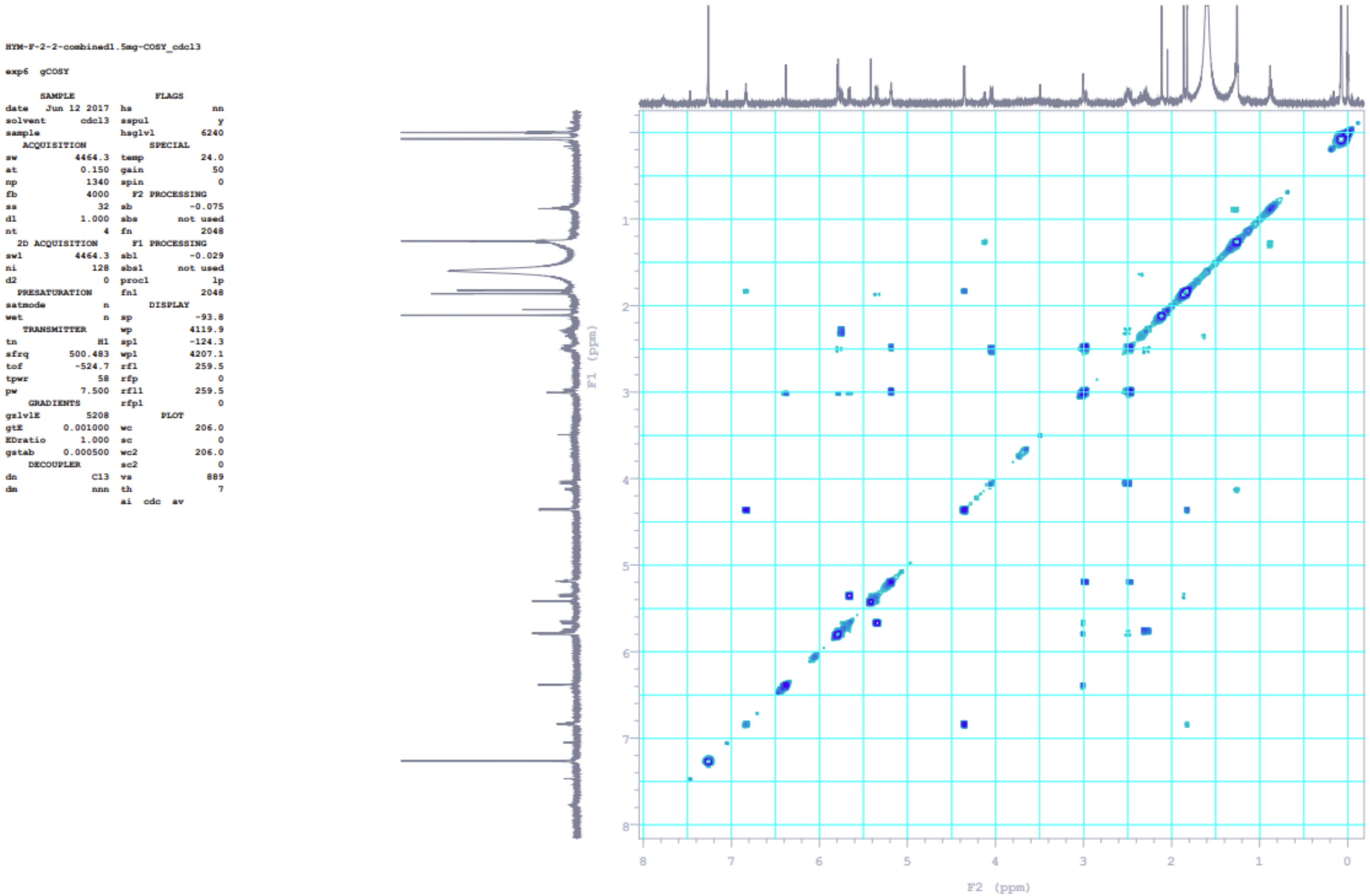

**Figure S18.** HSQC spectrum of **18** (measured in $CDCl_3$, 500 MHz).

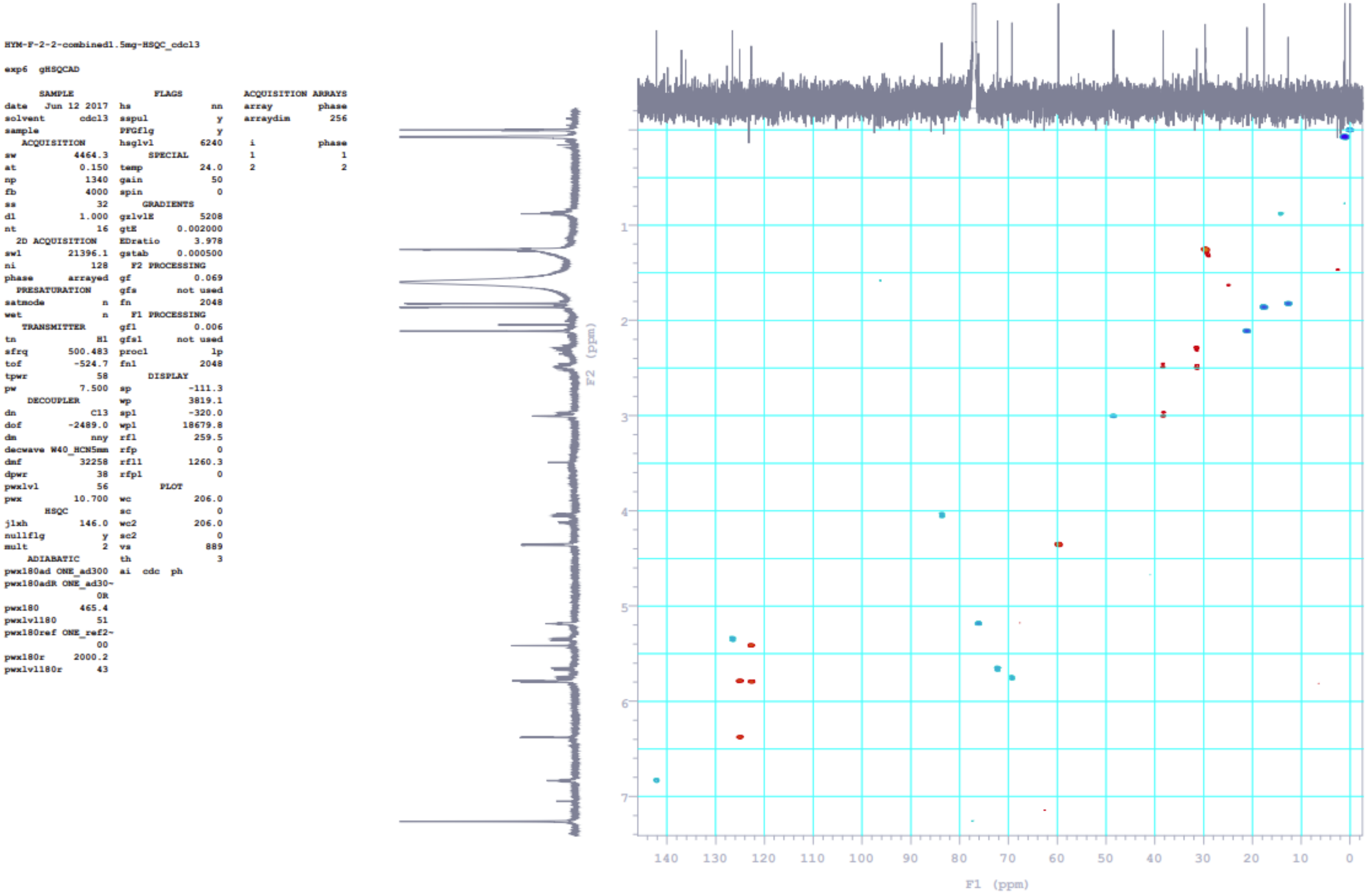

**Figure S19.** HMBC spectrum of **18** (measured in $CDCl_3$, 500 MHz).

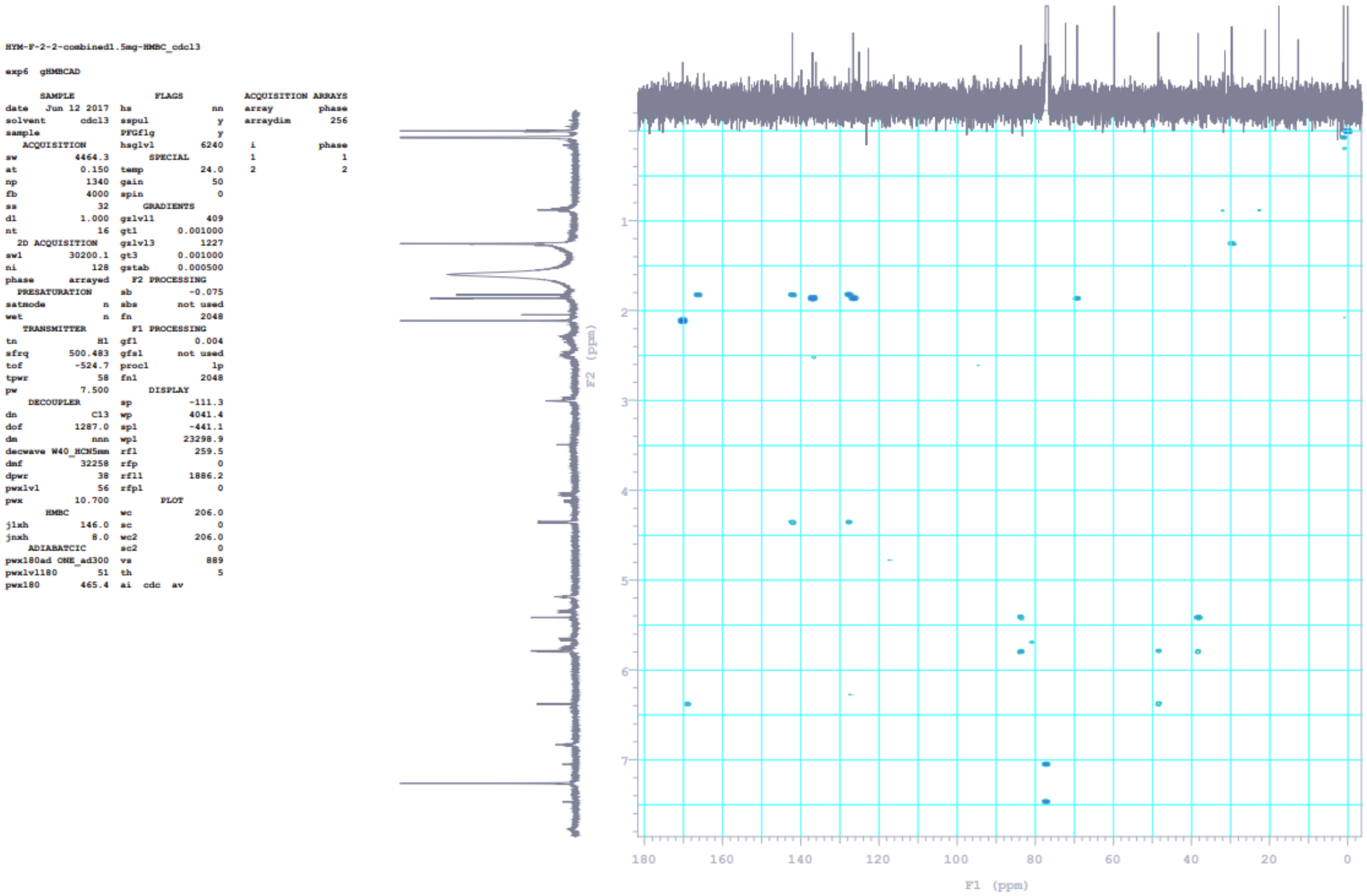

**Figure S20.** NOESY spectrum of **18** (measured in $CDCl_3$, 500 MHz).

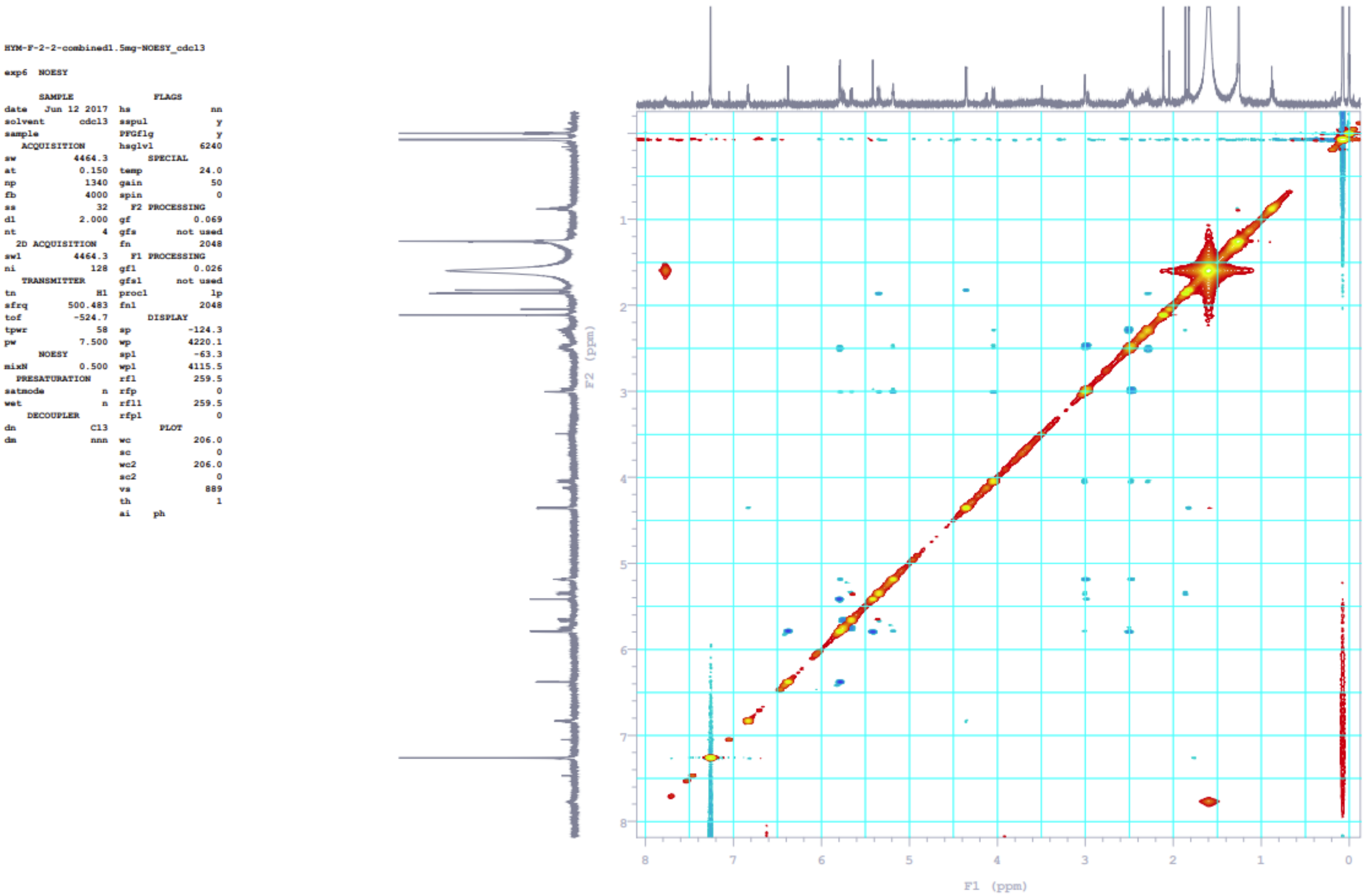

**Figure S21.** $^{1}$H NMR spectrum of **24** (measured in $CDCl_3$, 500 MHz).

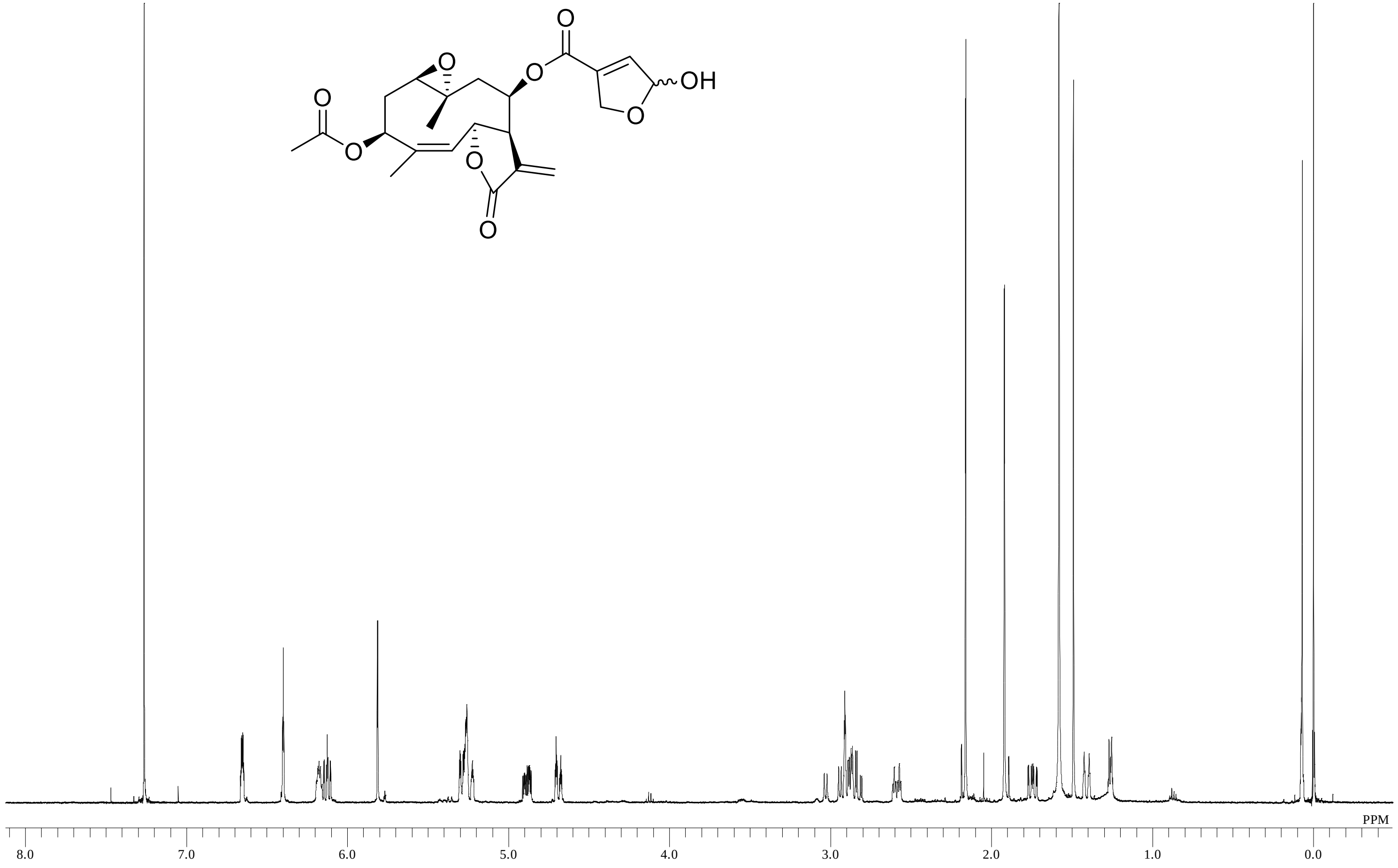

**Figure S22.** $^{13}C$ NMR spectrum of **24** (measured in $CDCl_3$, 126 MHz).

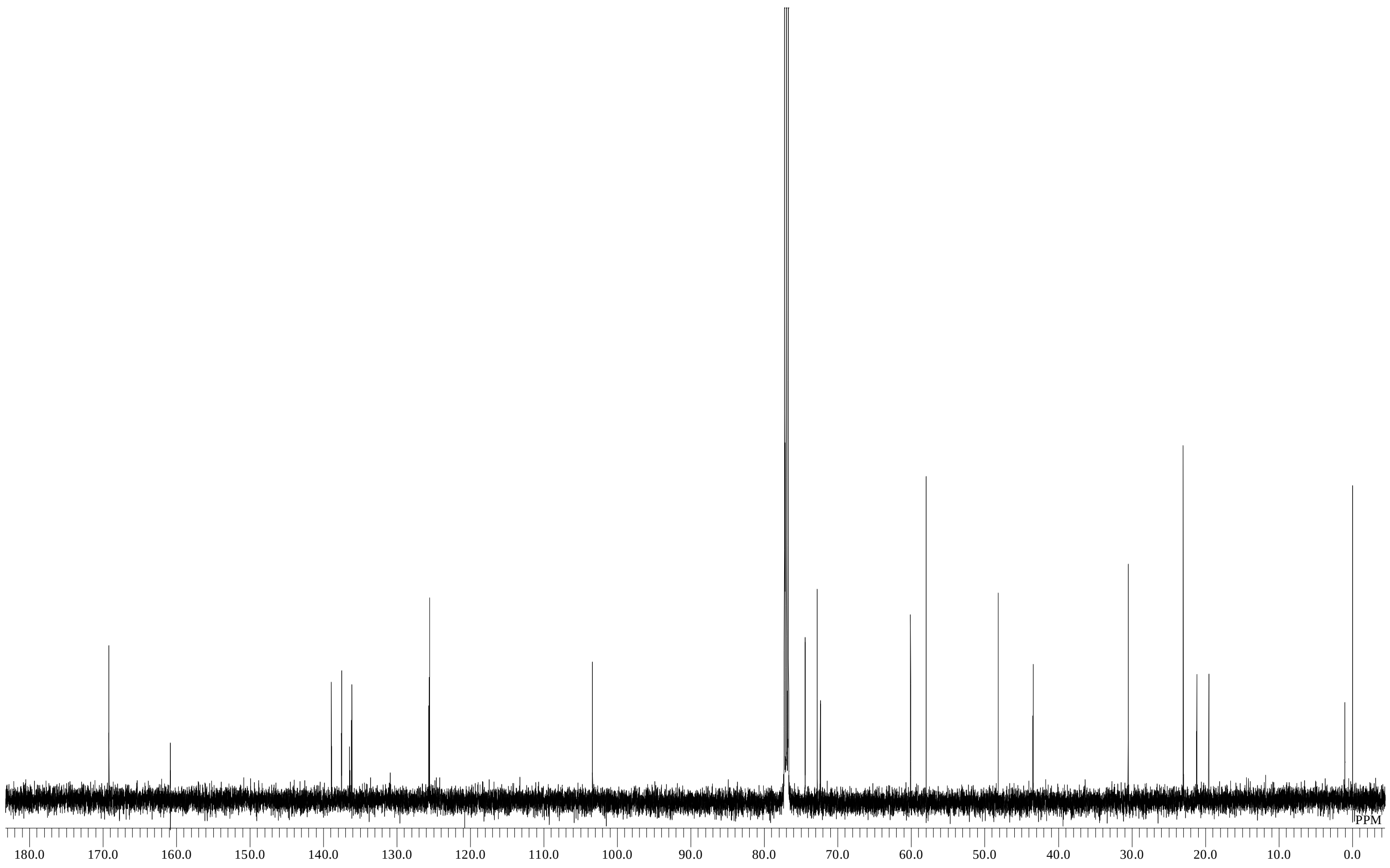

**Figure S23.** ¹H-¹H COSY spectrum of **24** (measured in $CDCl_3$, 500 MHz).

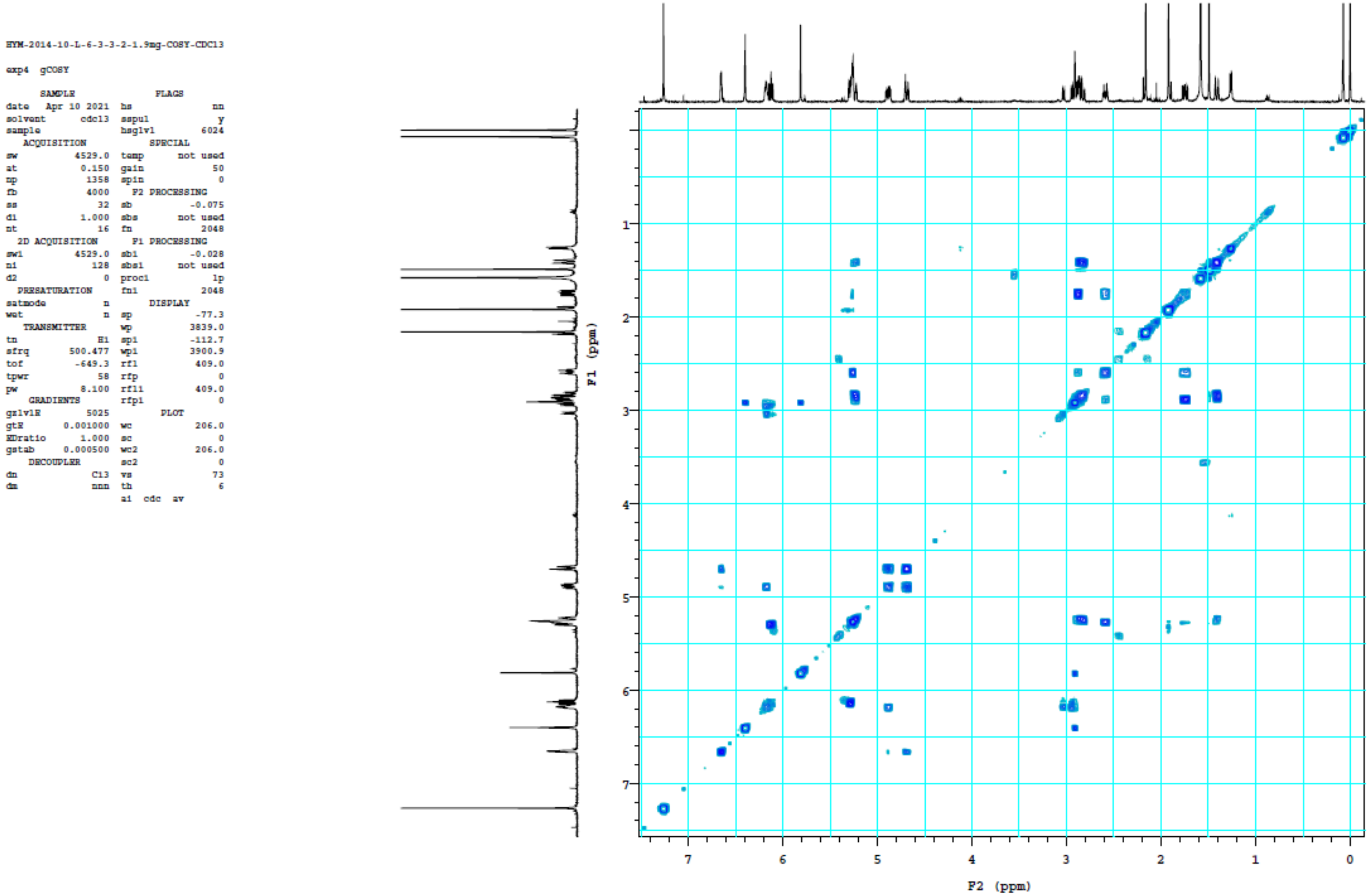

**Figure S24.** HSQC spectrum of **24** (measured in $CDCl_3$, 500 MHz).

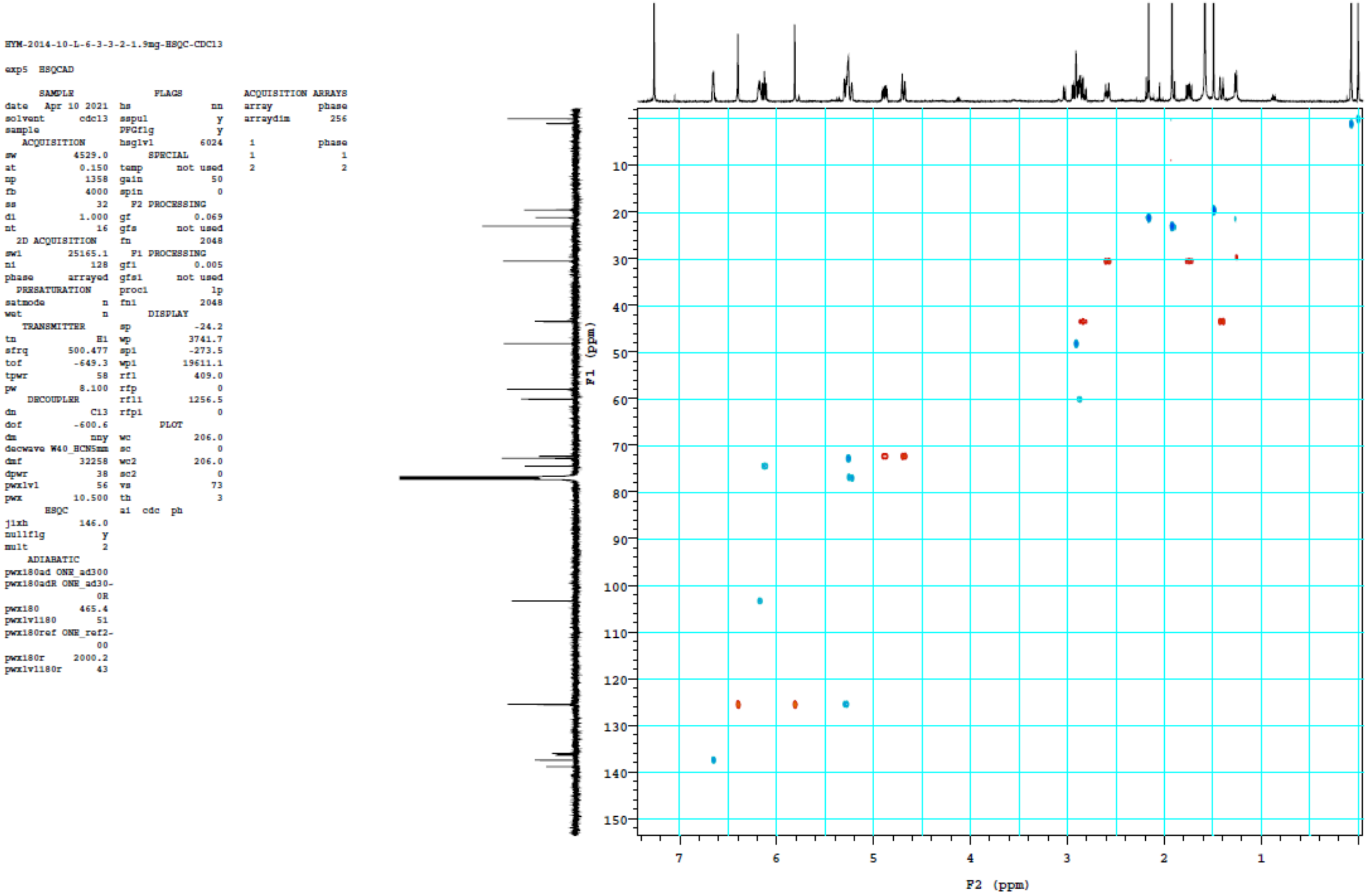

**Figure S25.** HMBC spectrum of **24** (measured in $CDCl_3$, 500 MHz).

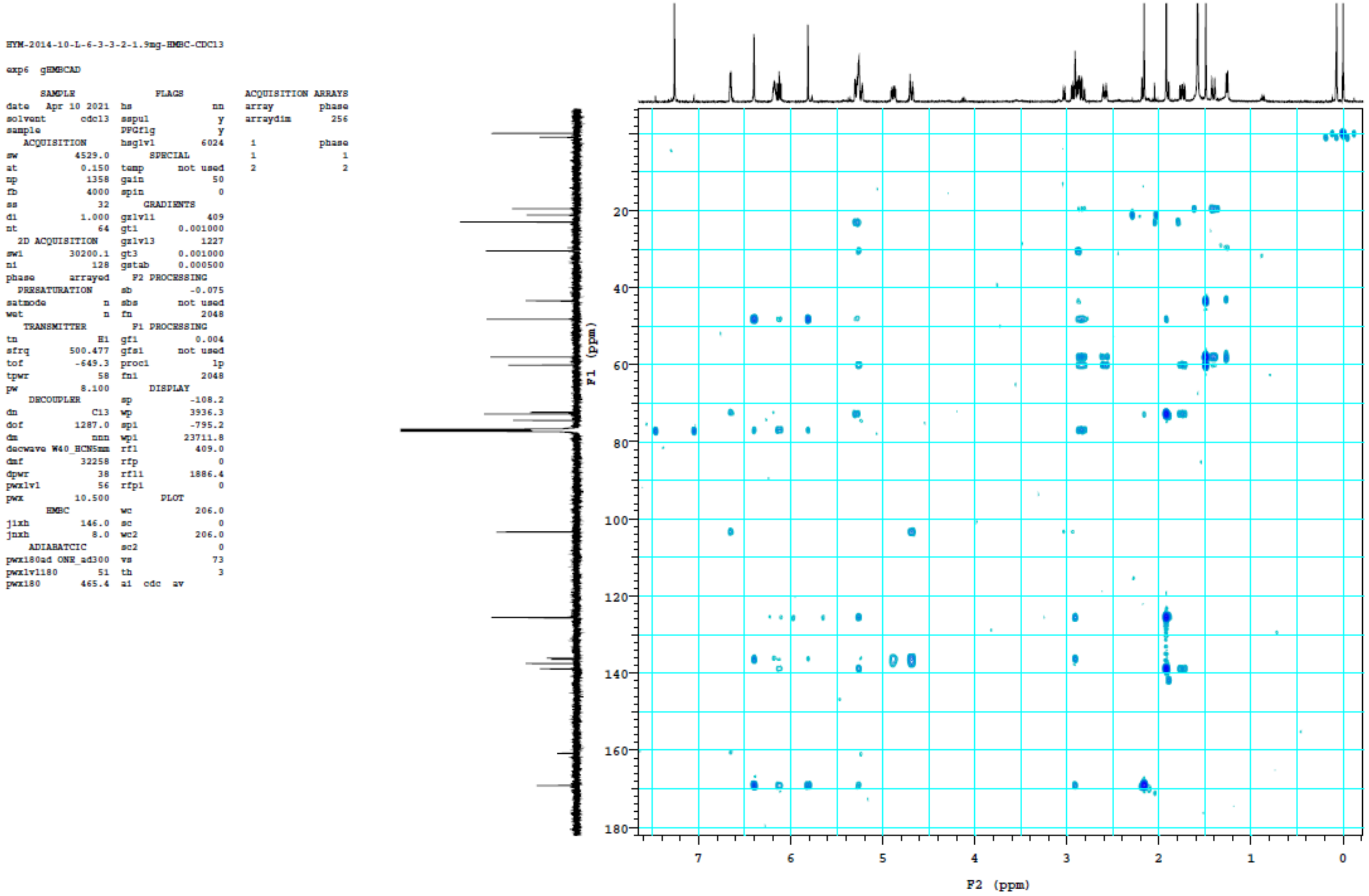

**Figure S26.** NOESY spectrum of **24** (measured in $CDCl_3$, 500 MHz).

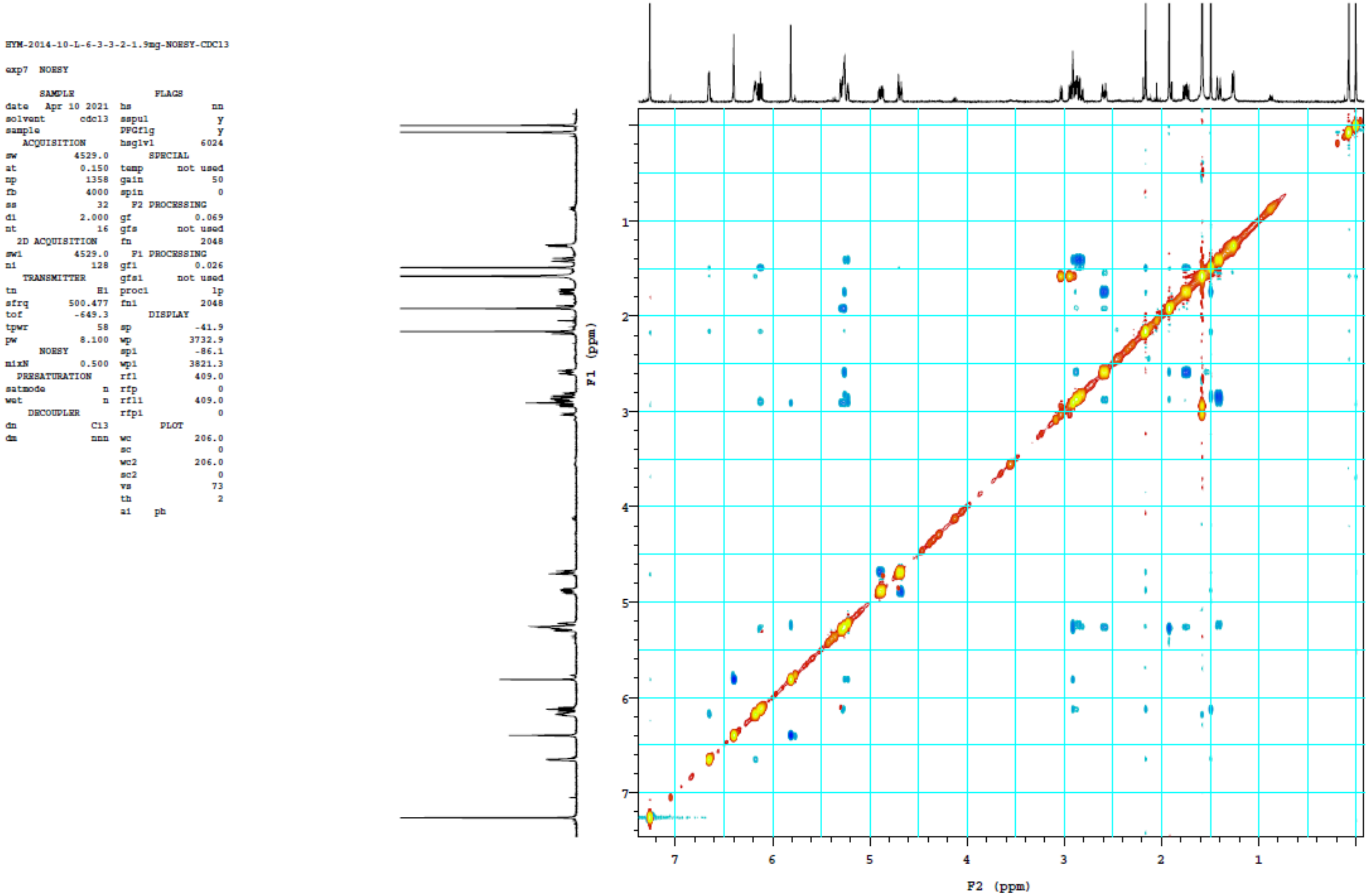

**Figure S27.** $^1$H NMR spectrum of **31** (measured in $CDCl_3$, 500 MHz).

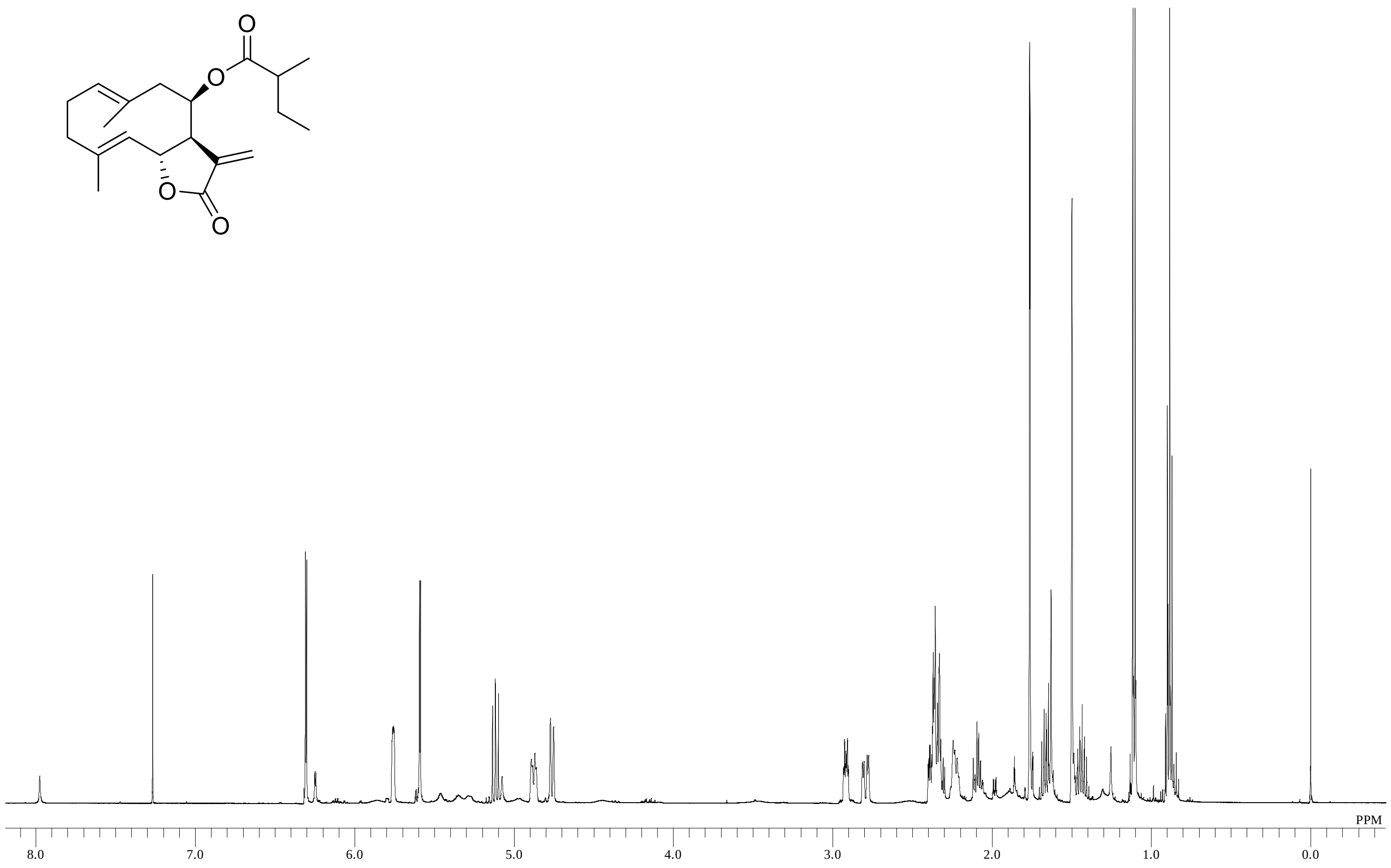

**Figure S28.** $^{13}$C NMR spectrum of **31** (measured in $CDCl_3$, 126 MHz).

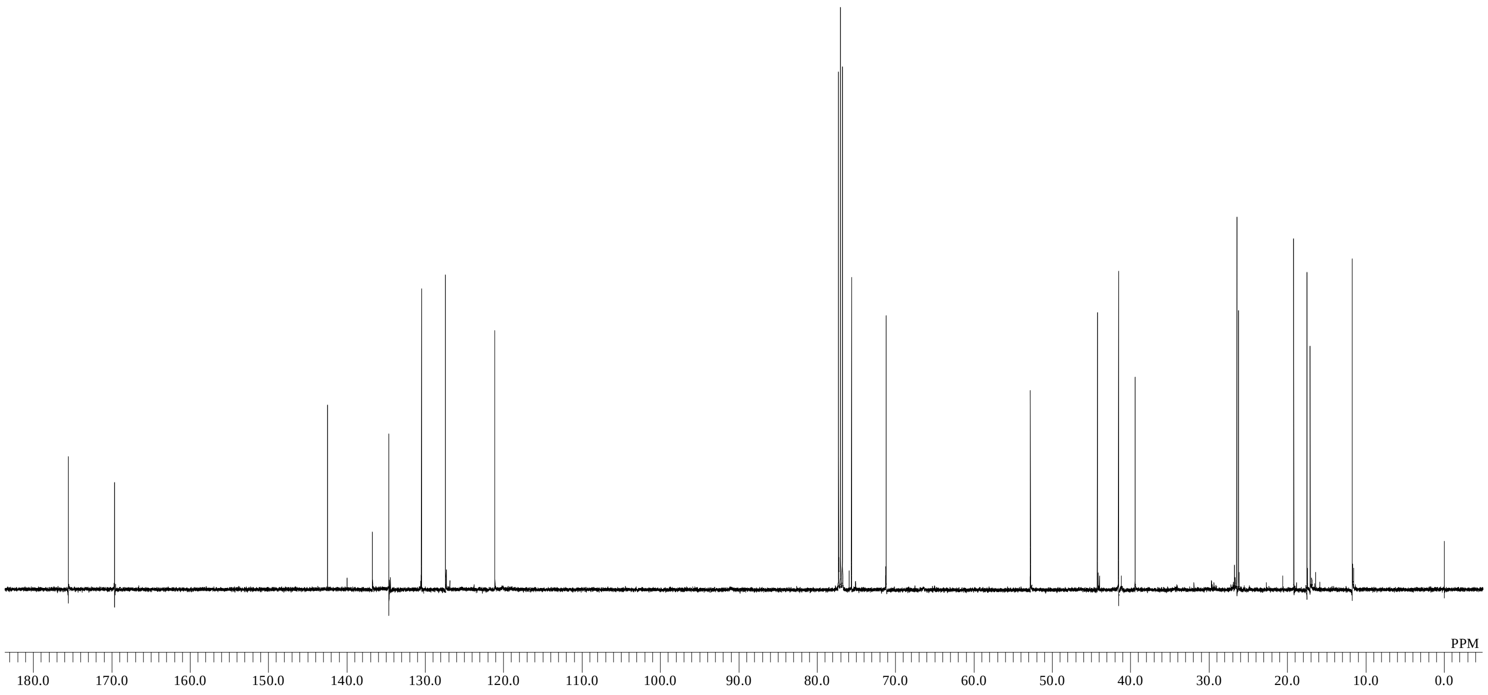

**Figure S29.** $^{1}$H-$^{1}$H COSY spectrum of **31** (measured in $CDCl_3$, 500 MHz).

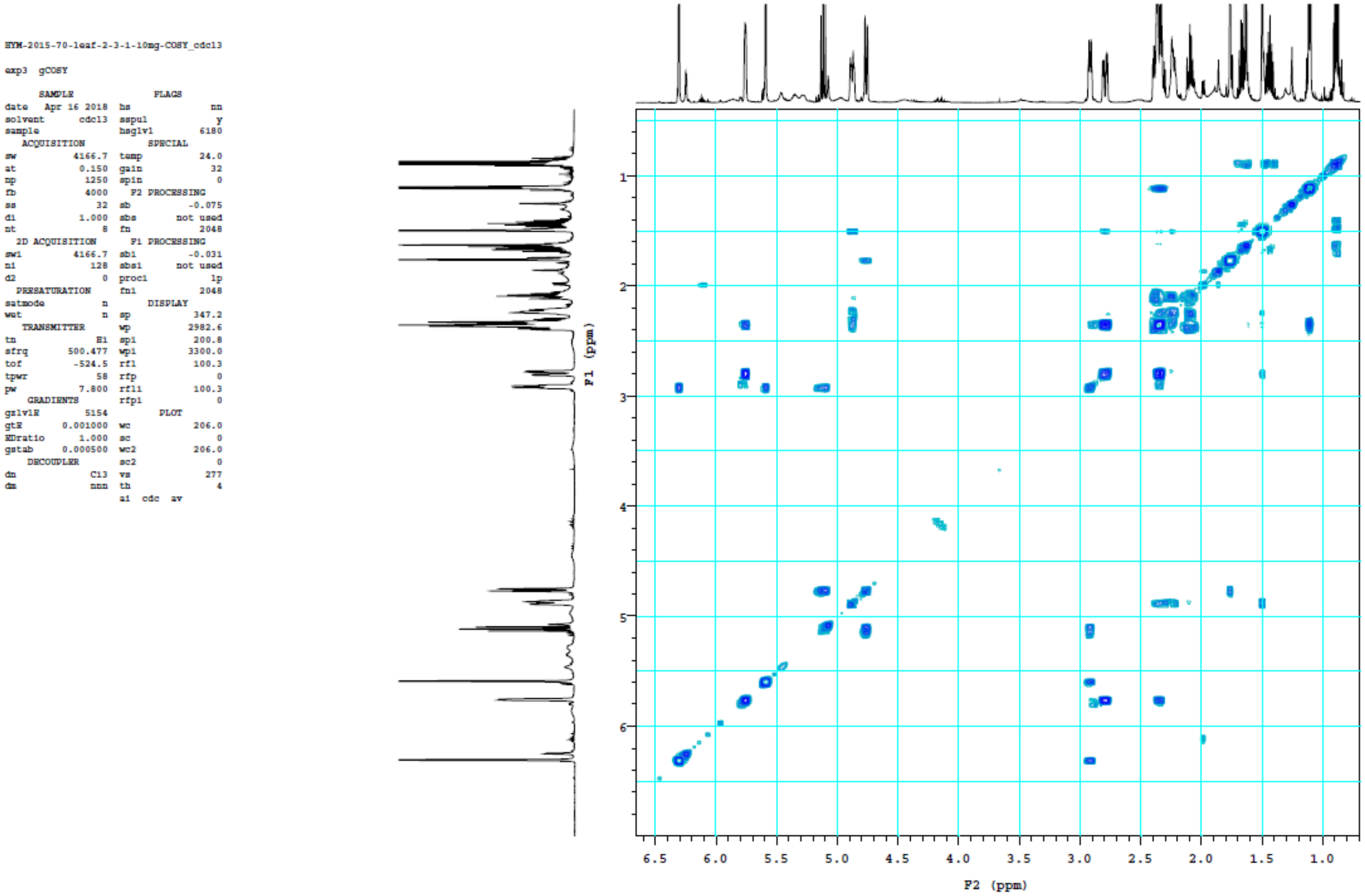

**Figure S30.** HSQC spectrum of **31** (measured in $CDCl_3$, 500 MHz).

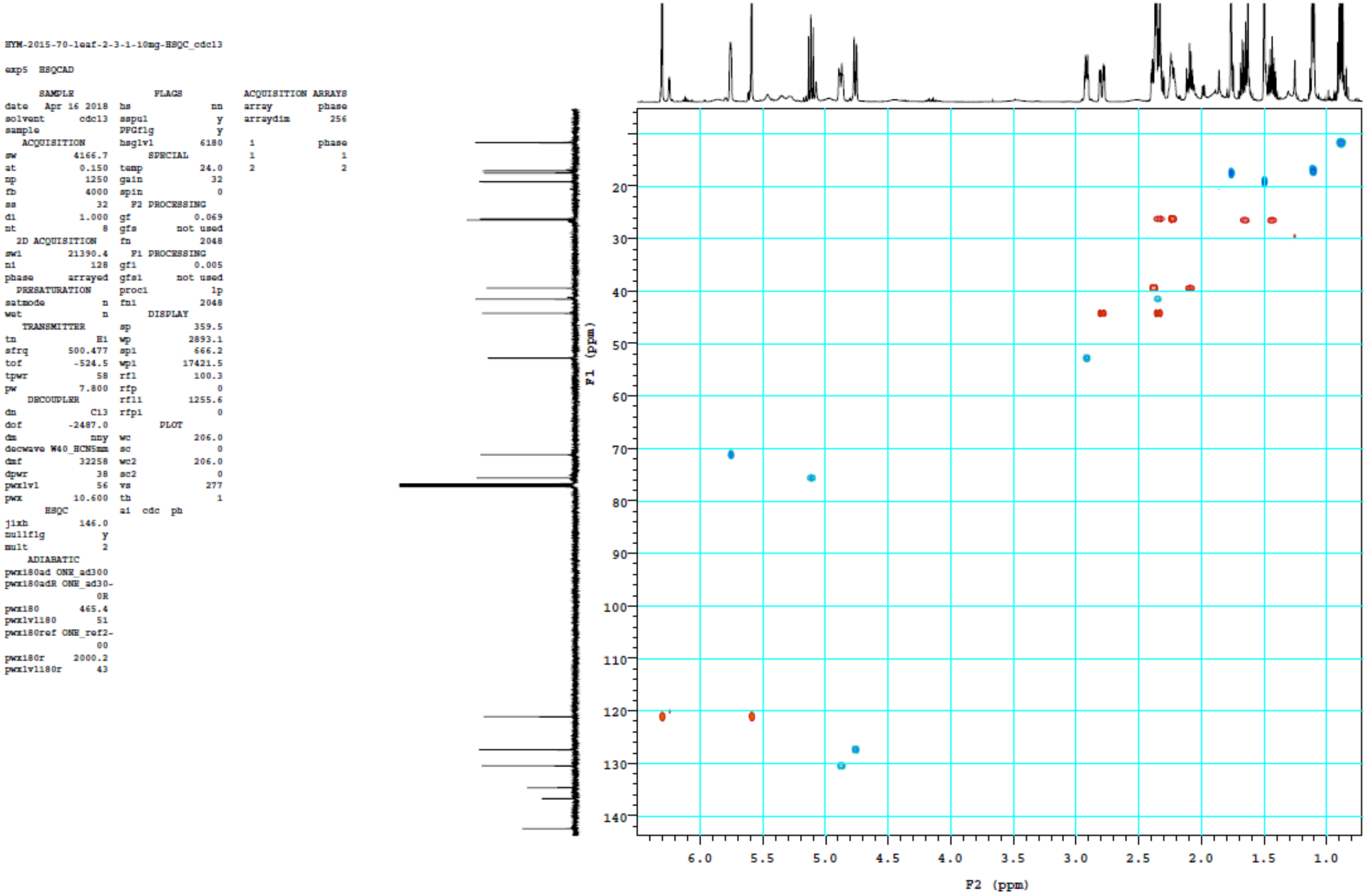

**Figure S31.** HMBC spectrum of **31** (measured in $CDCl_3$, 500 MHz).

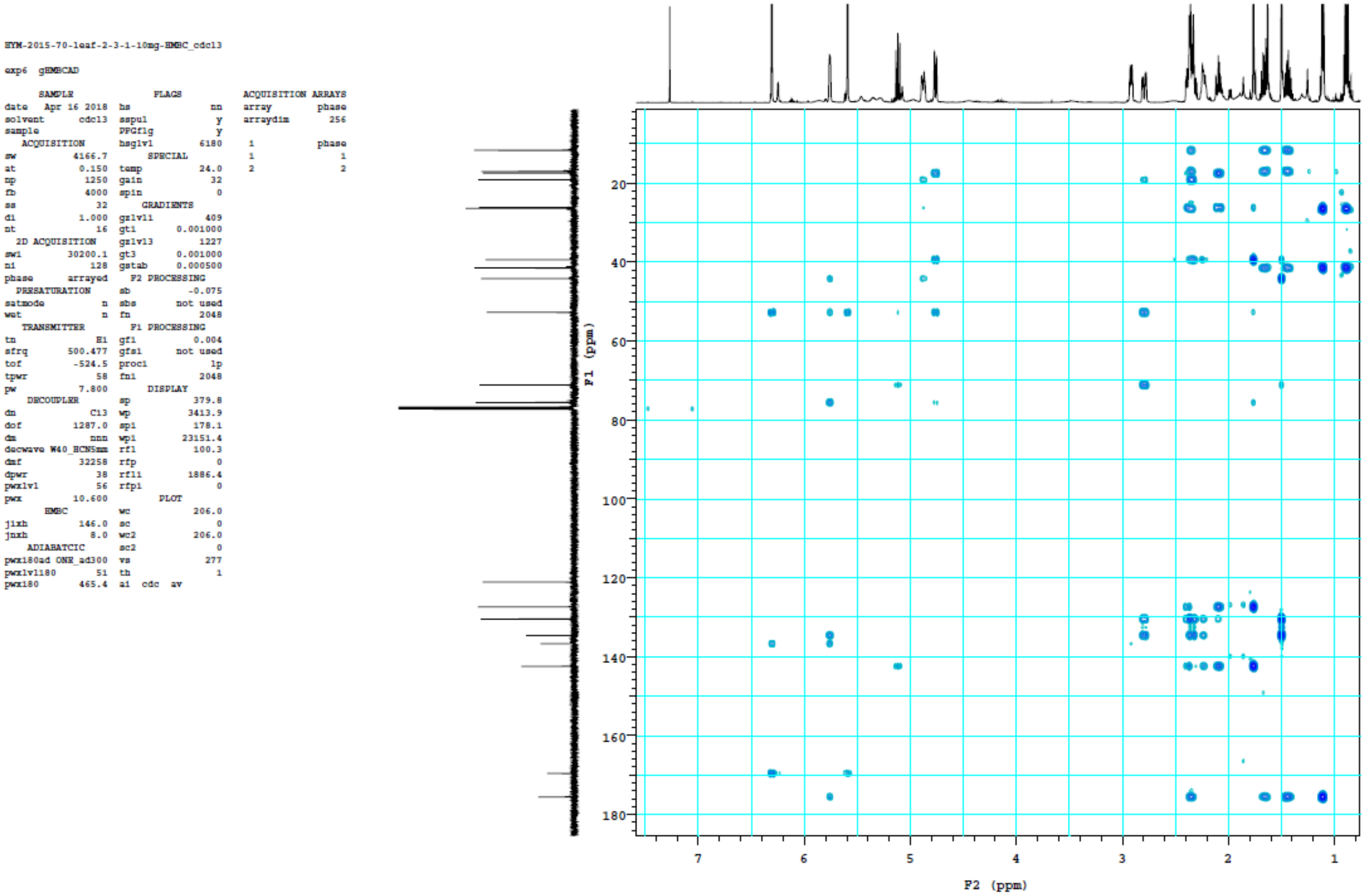

**Figure S32.** NOESY spectrum of **31** (measured in $CDCl_3$, 500 MHz).

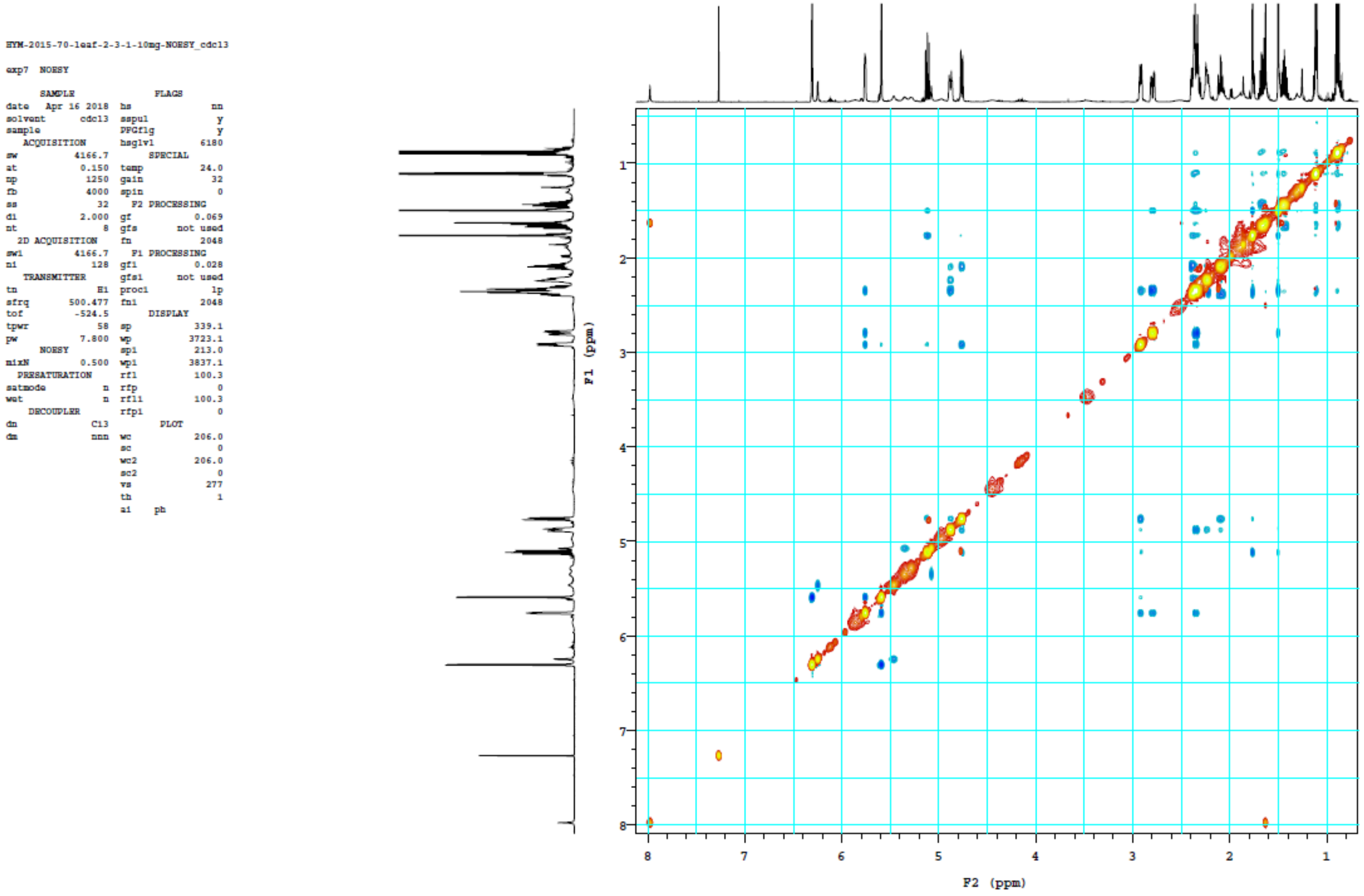

**Figure S33.** $^{1}$H NMR spectrum of **36** (measured in $CDCl_3$, 500 MHz, 298 K).

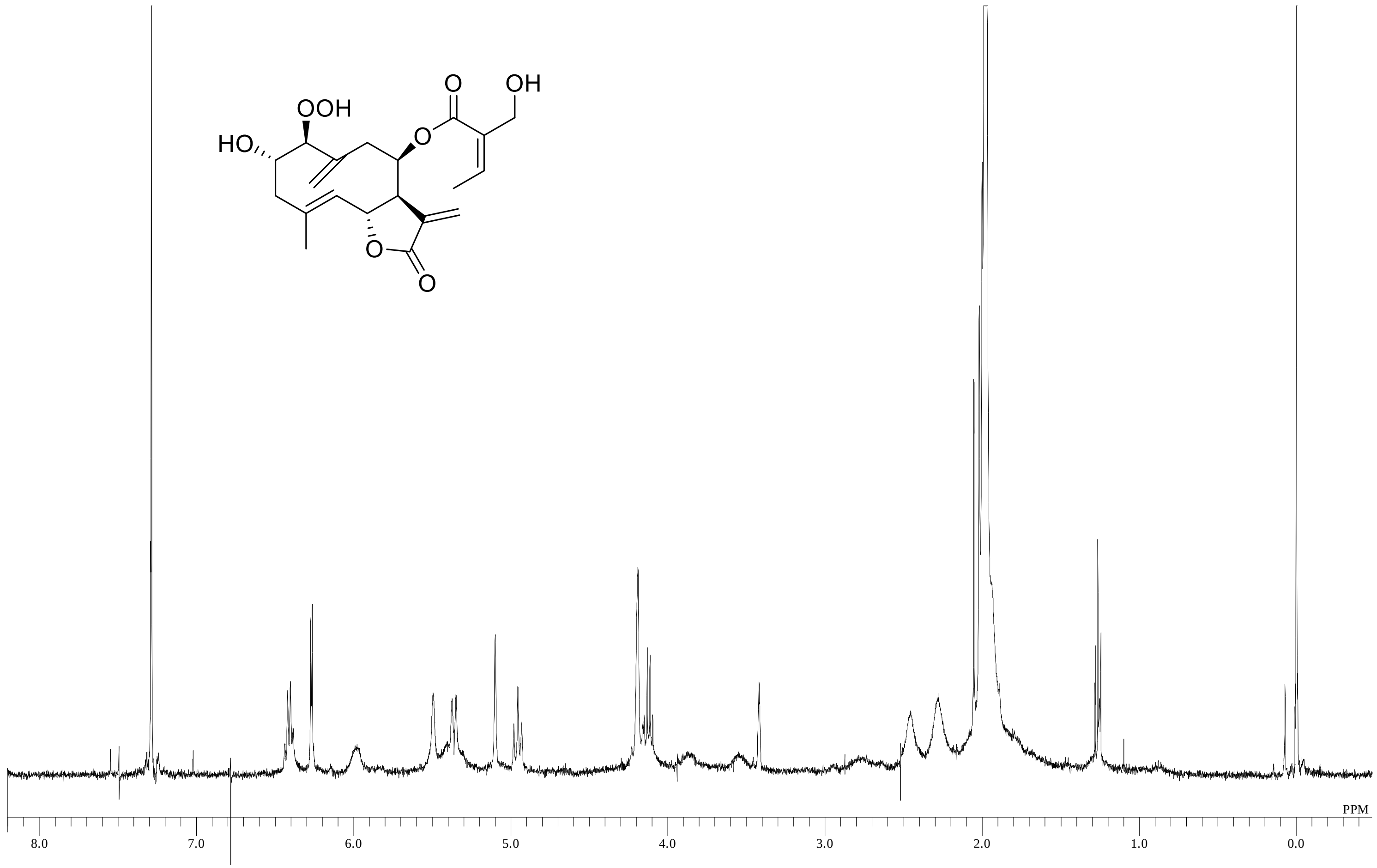

**Figure S34.** $^{1}$H NMR spectrum of **36** (measured in $CDCl_3$, 400 MHz, 233 K).

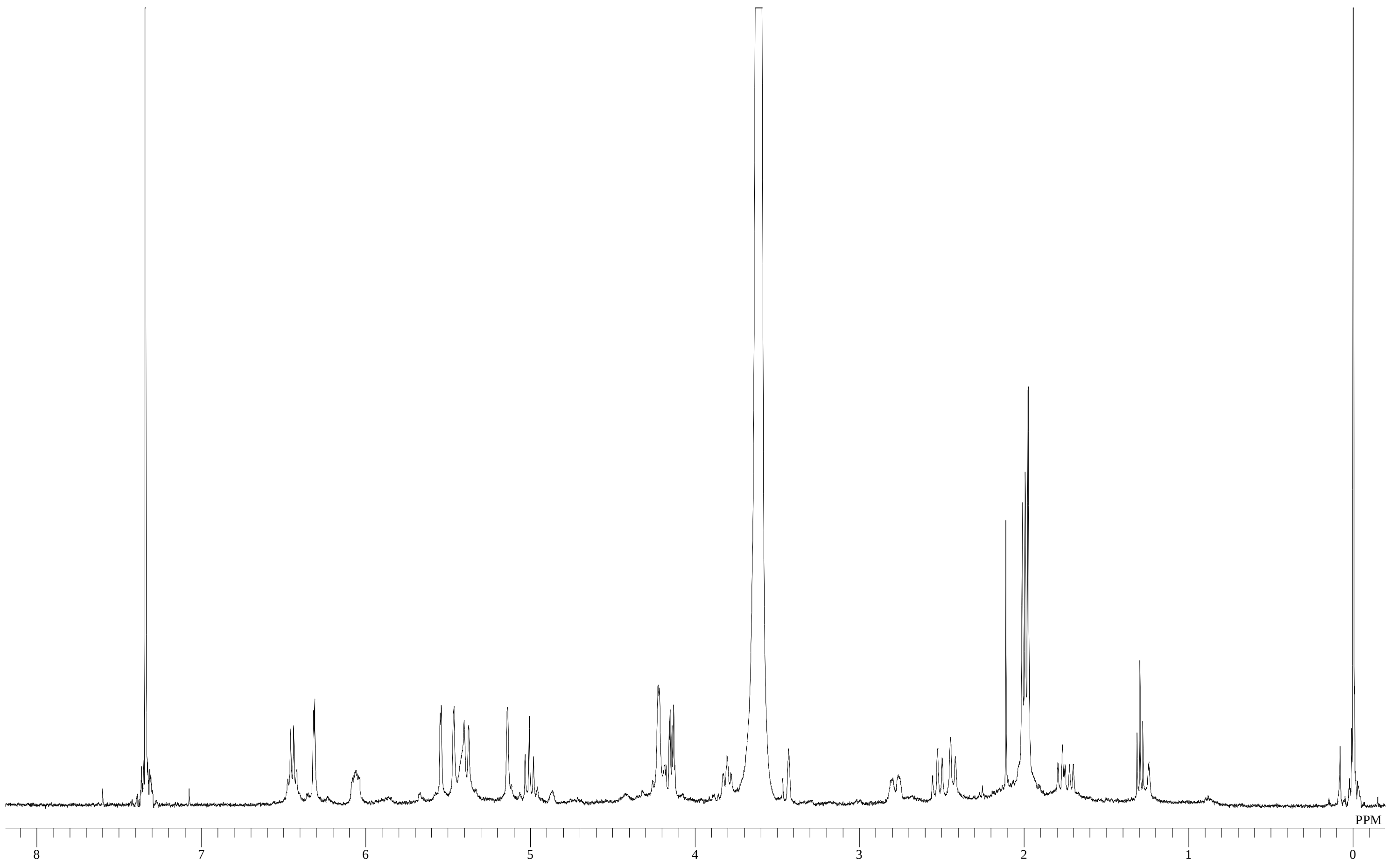

**Figure S35.** $^{13}$C NMR spectrum of **36** (measured in $CDCl_3$, 100 MHz, 233 K).

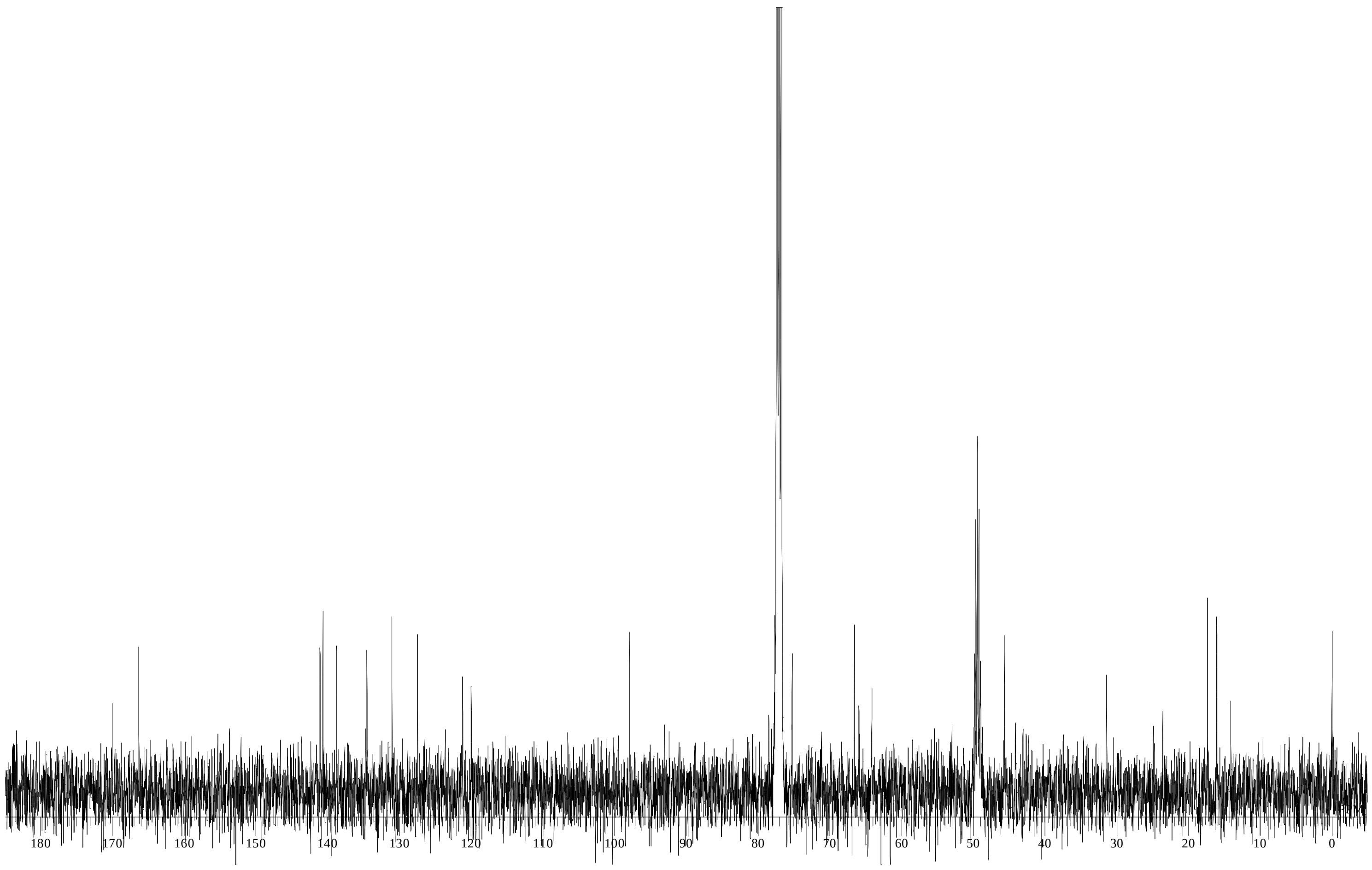

**Figure S36.** $^{1}H$-$^{1}H$ COSY spectrum of **36** (measured in $CDCl_3$, 400 MHz, 233 K).

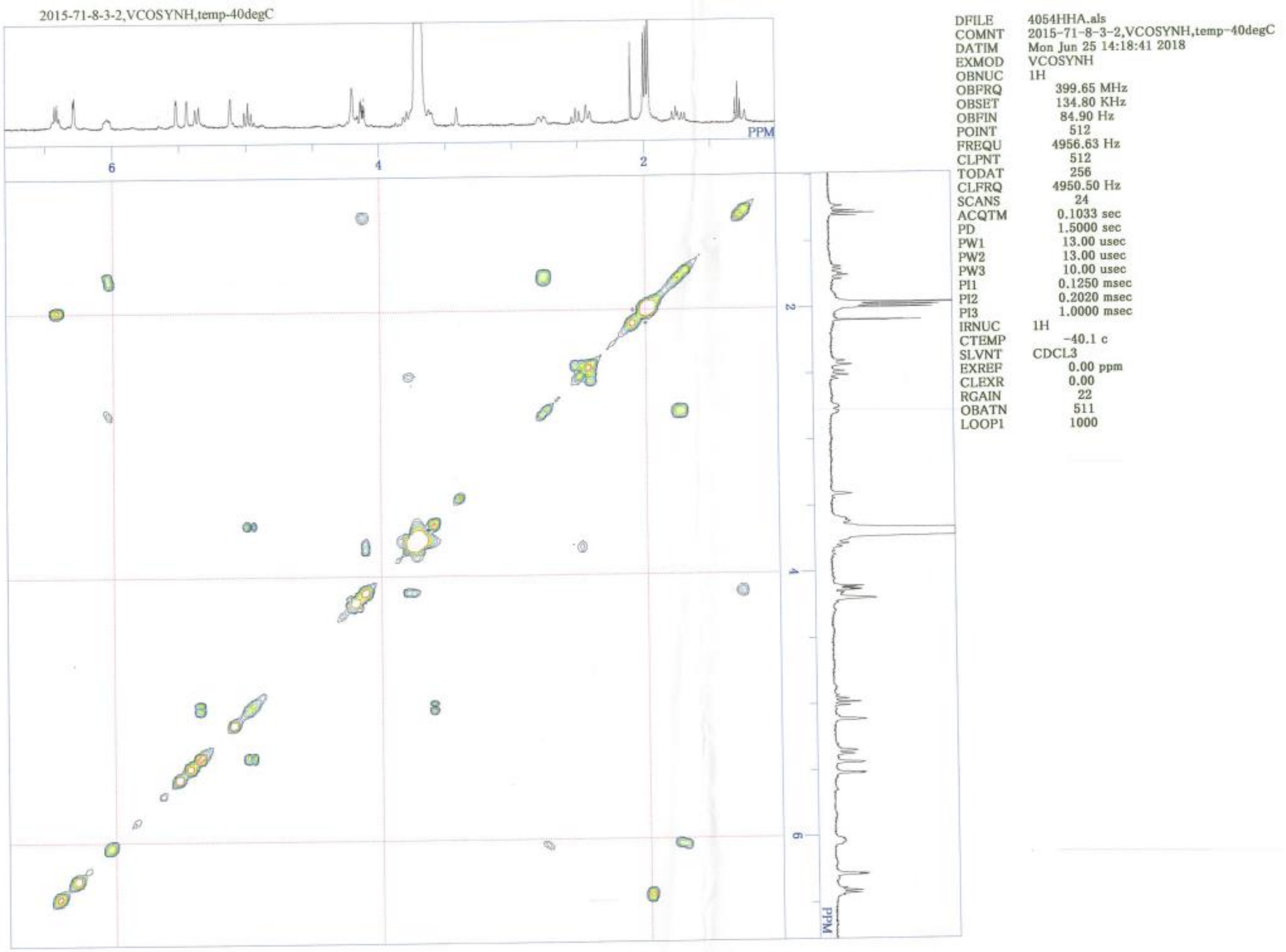

**Figure S37.** HSQC spectrum of **36** (measured in $CDCl_3$, 400 MHz, 233 K).

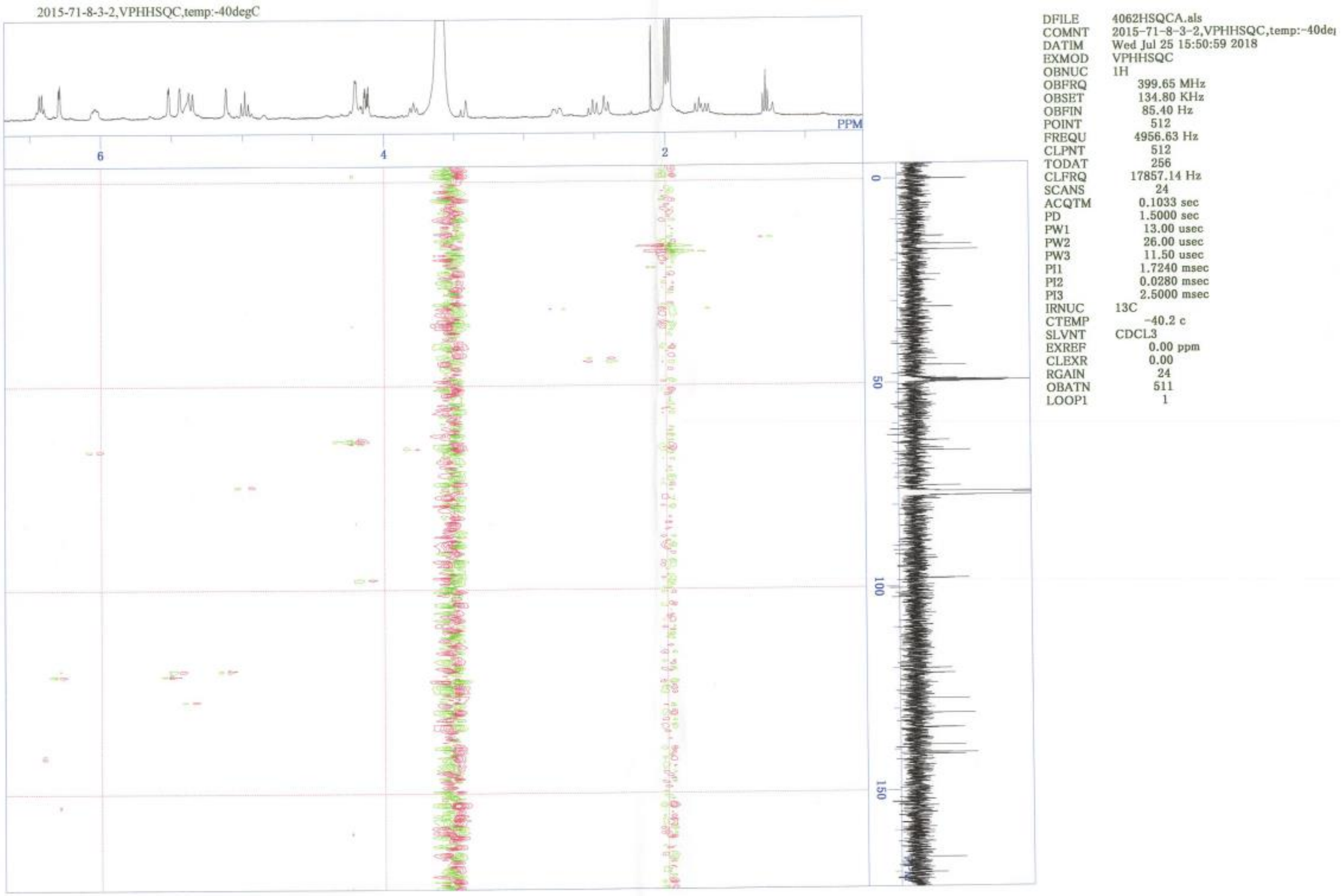

**Figure S38.** HMBC spectrum of **36** (measured in $CDCl_3$, 400 MHz, 233 K).

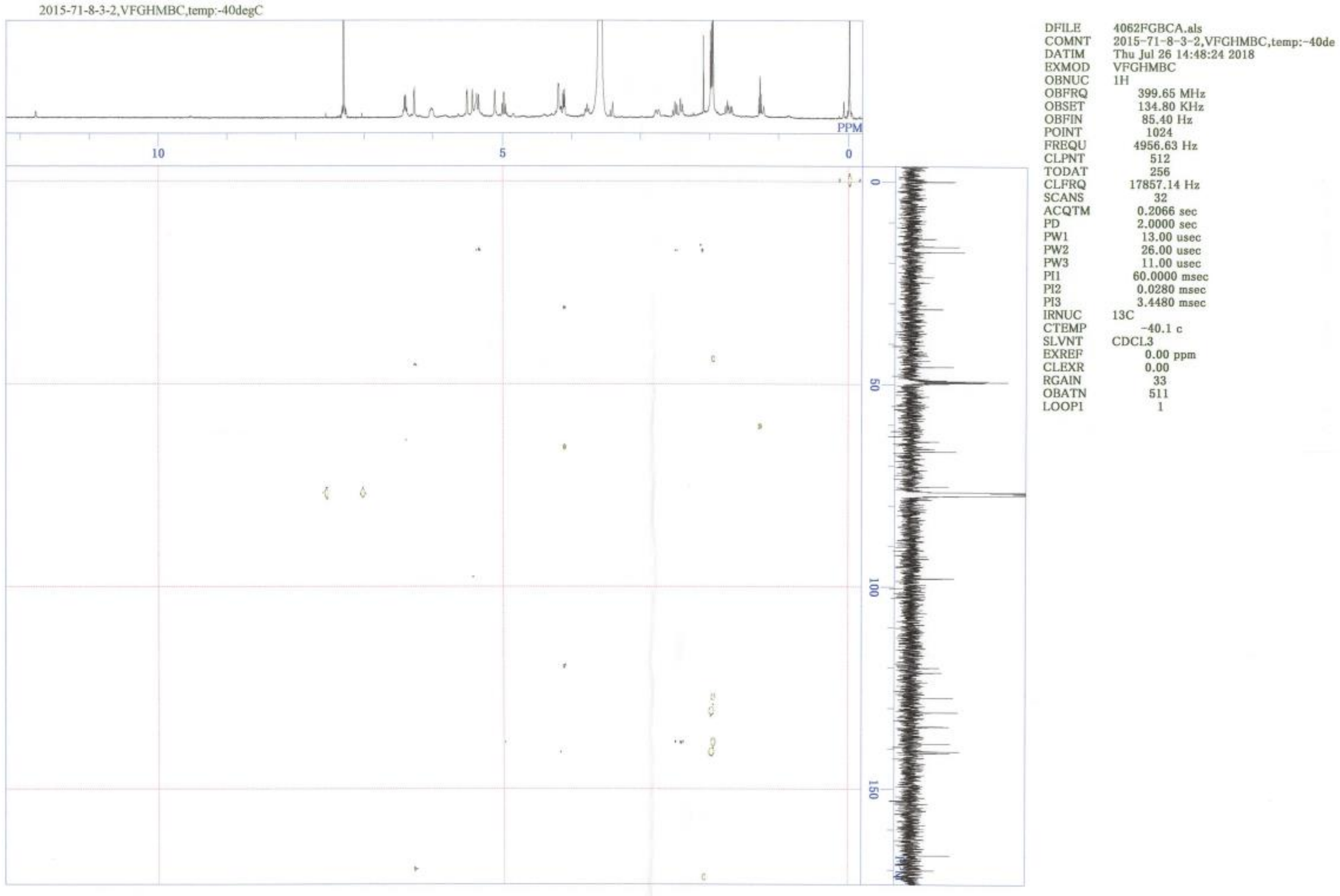

**Figure S39.** NOESY spectrum of **36** (measured in $CDCl_3$, 400 MHz, 233 K).

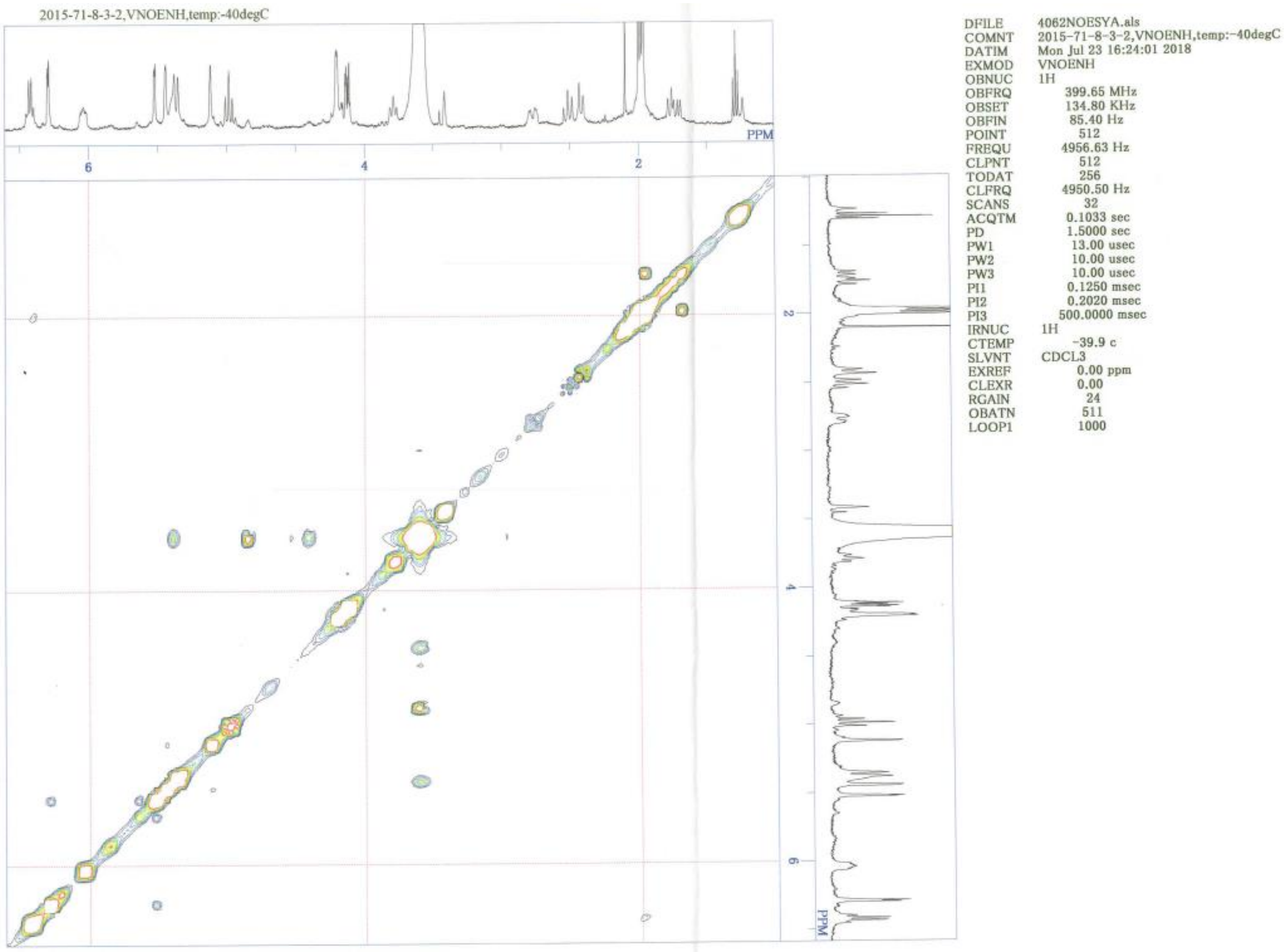

**Figure S40.** $^{1}$H NMR spectrum of **40** (measured in $CDCl_3$, 500 MHz, 298 K).

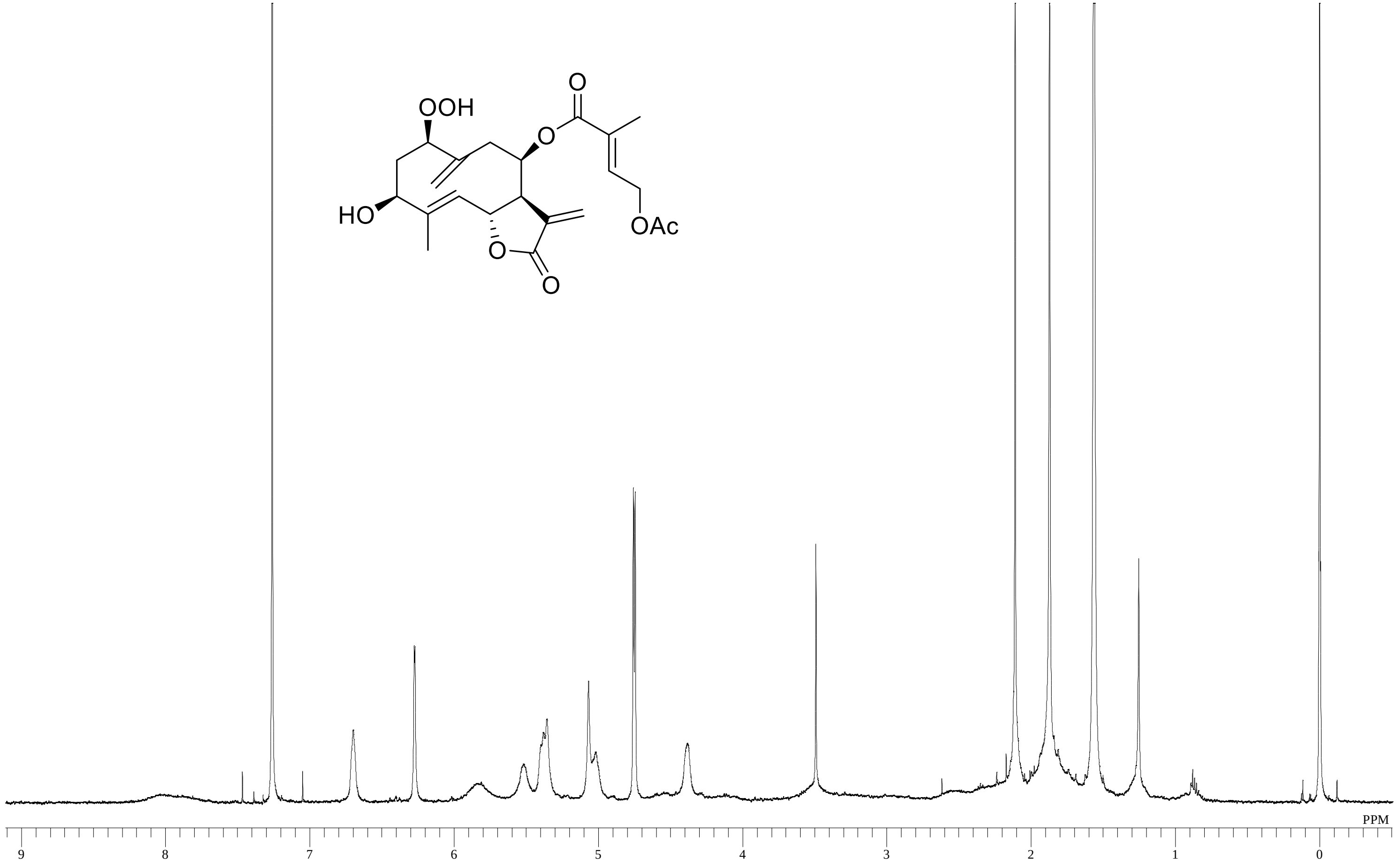

**Figure S41.** $^1H$ NMR spectrum of **40** (measured in $CDCl_3$, 400 MHz, 233 K).

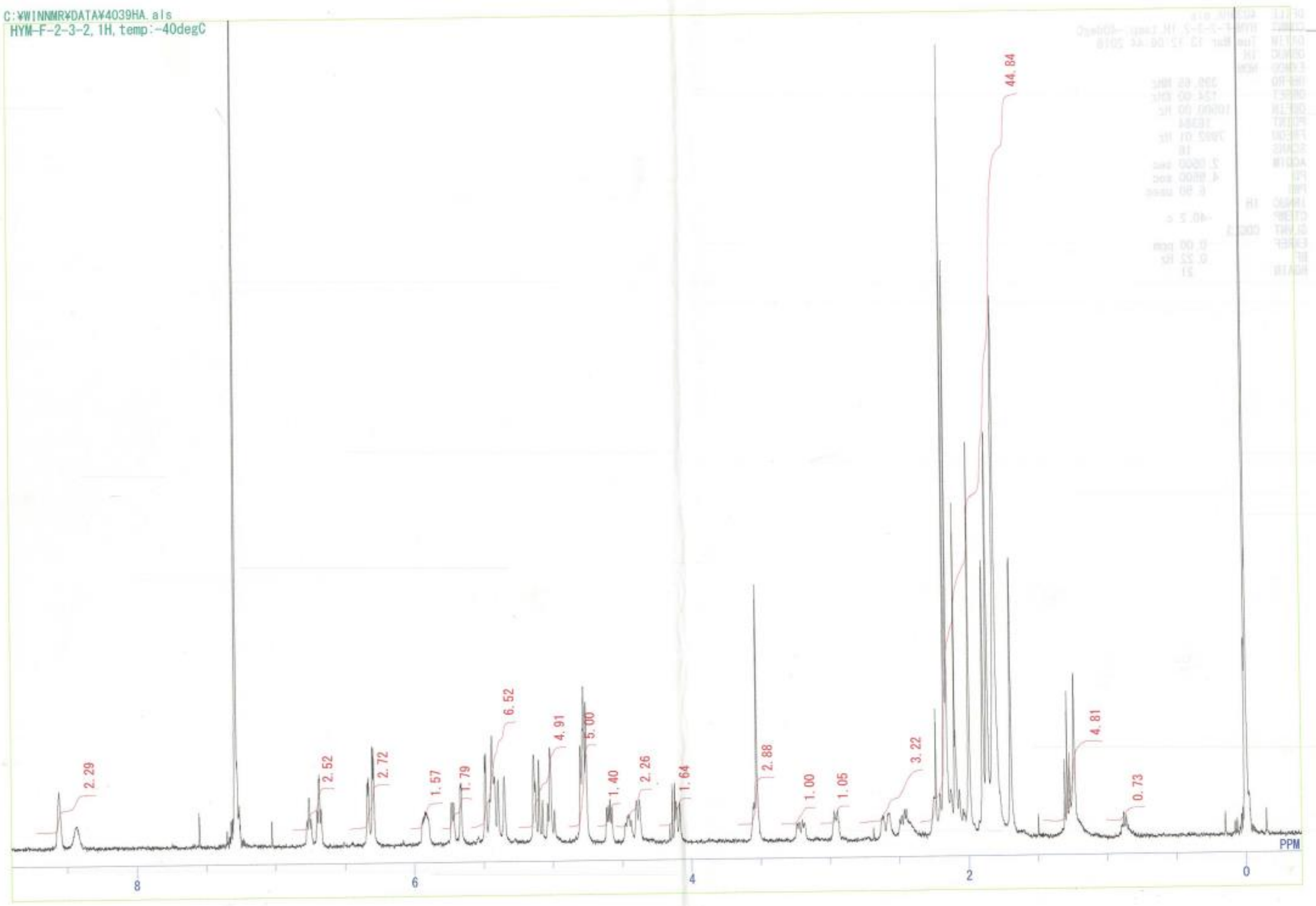

**Figure S42.** $^1H$-$^1H$ COSY spectrum of **40** (measured in $CDCl_3$, 400 MHz, 233 K).

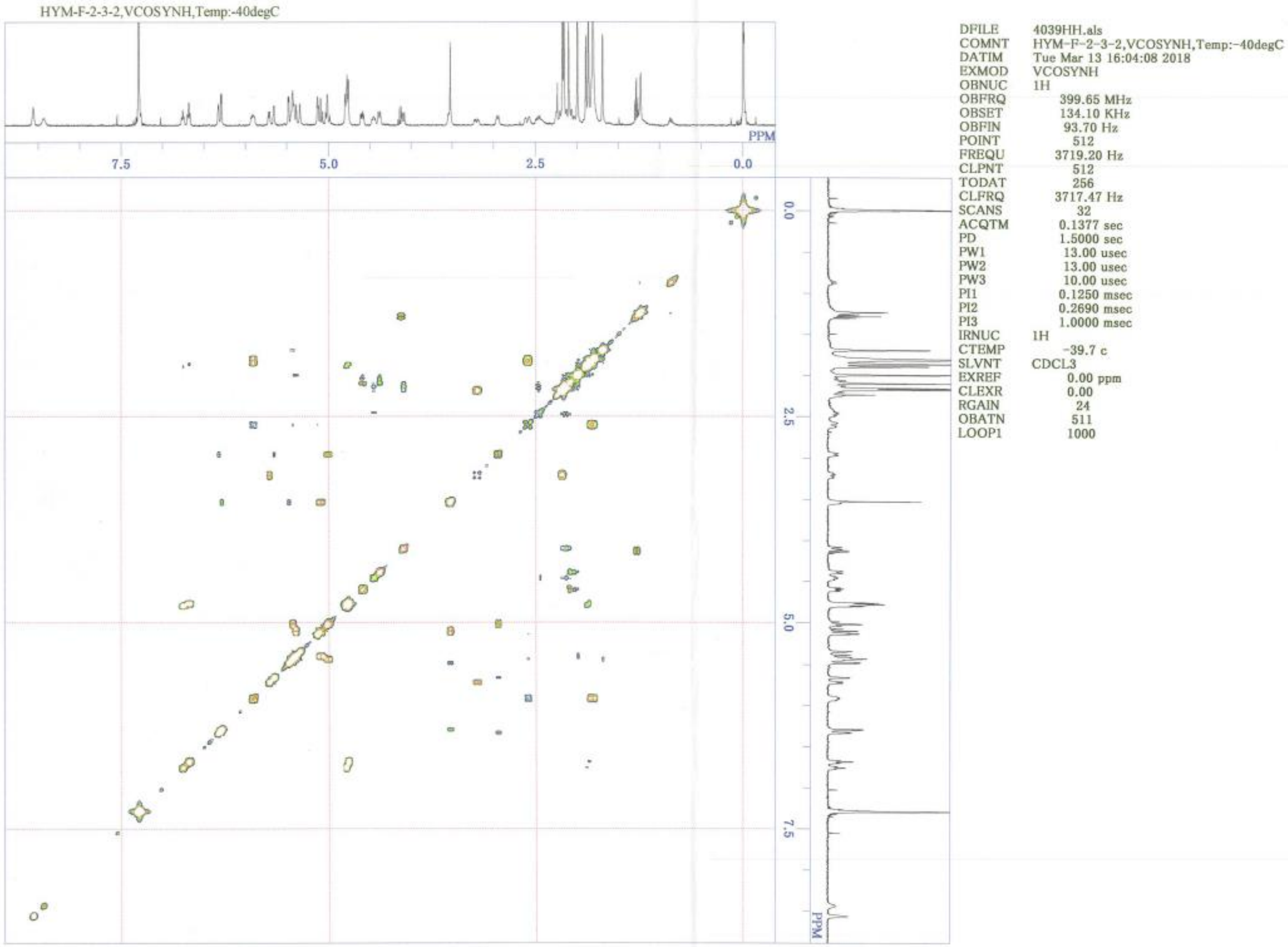

**Figure S43.** $^{1}$H NMR spectrum of **45** (measured in $CDCl_3$, 500 MHz).

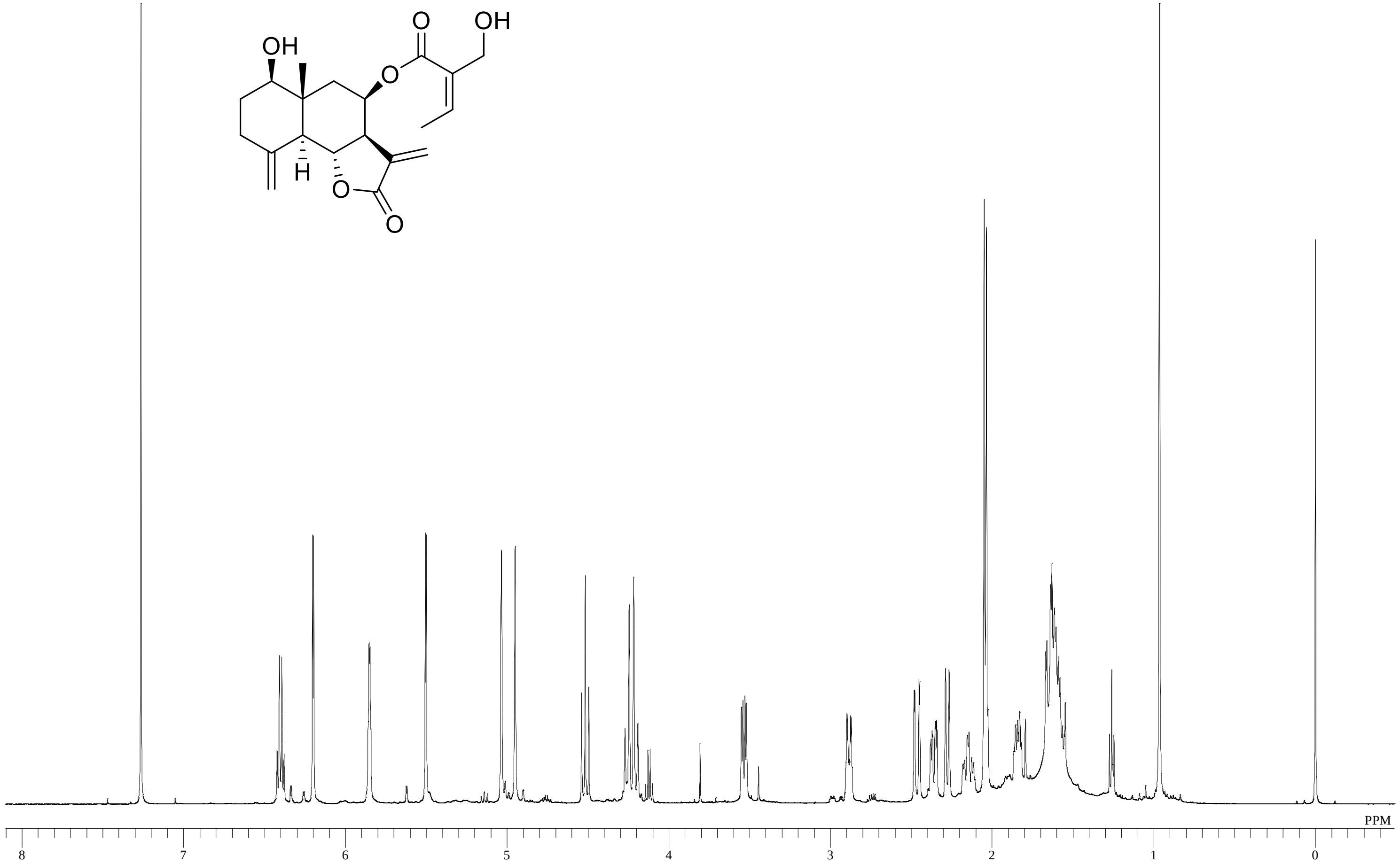

**Figure S44.** $^{13}C$ NMR spectrum of **45** (measured in $CDCl_3$, 126 MHz).

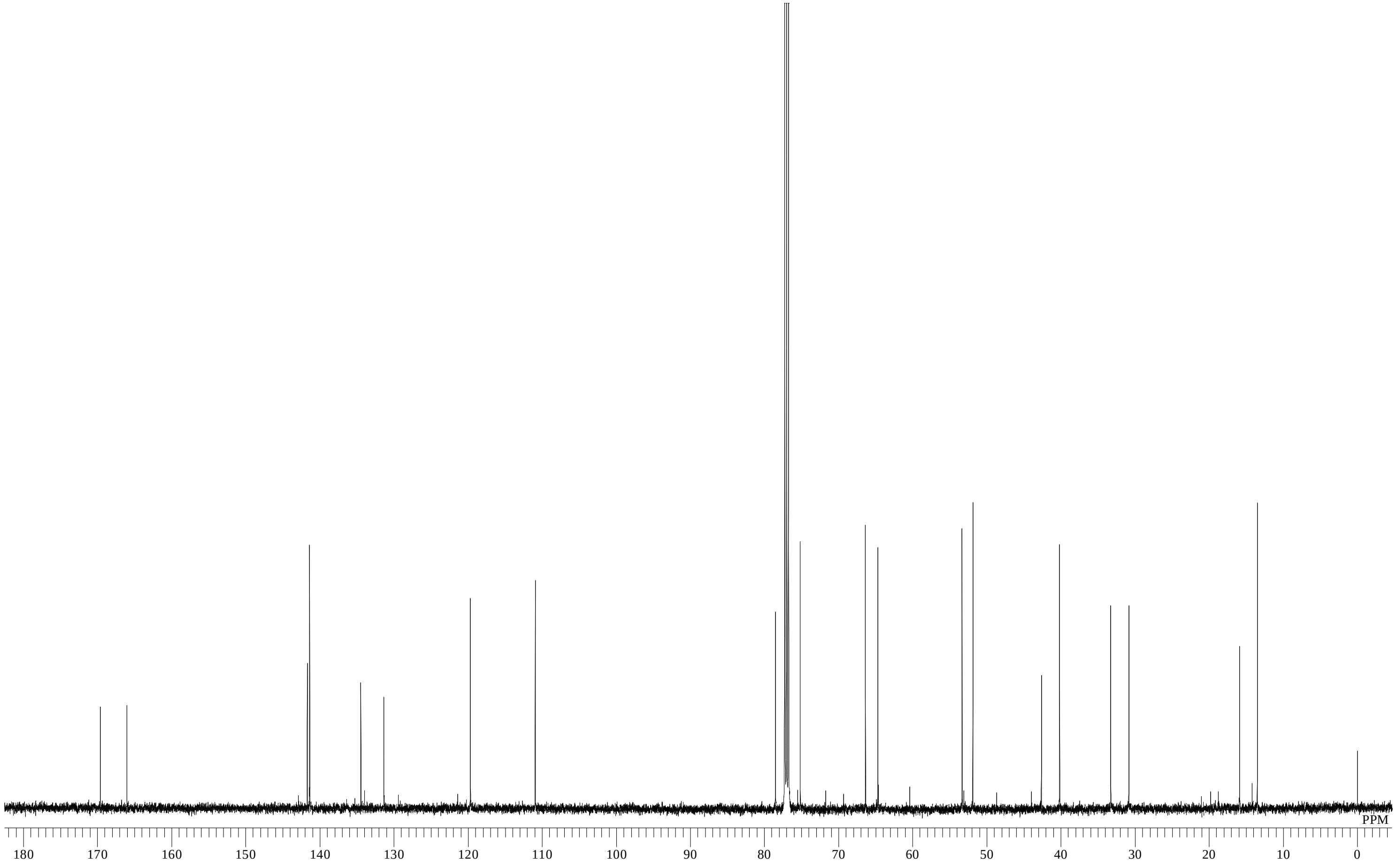

**Figure S45.** $^{1}$H-$^{1}$H COSY spectrum of **45** (measured in $CDCl_3$, 500 MHz).

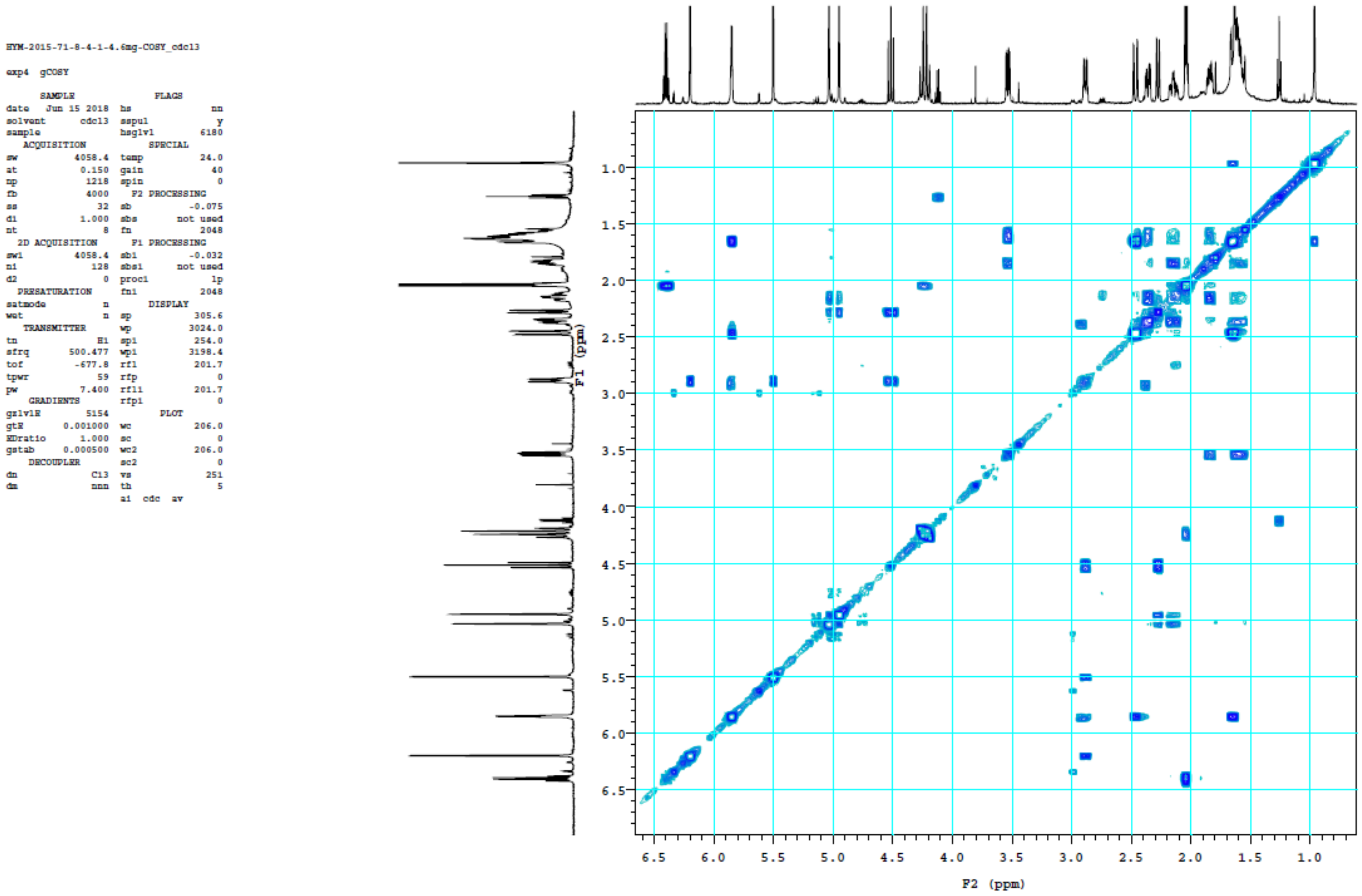

**Figure S46.** HSQC spectrum of **45** (measured in $CDCl_3$, 500 MHz).

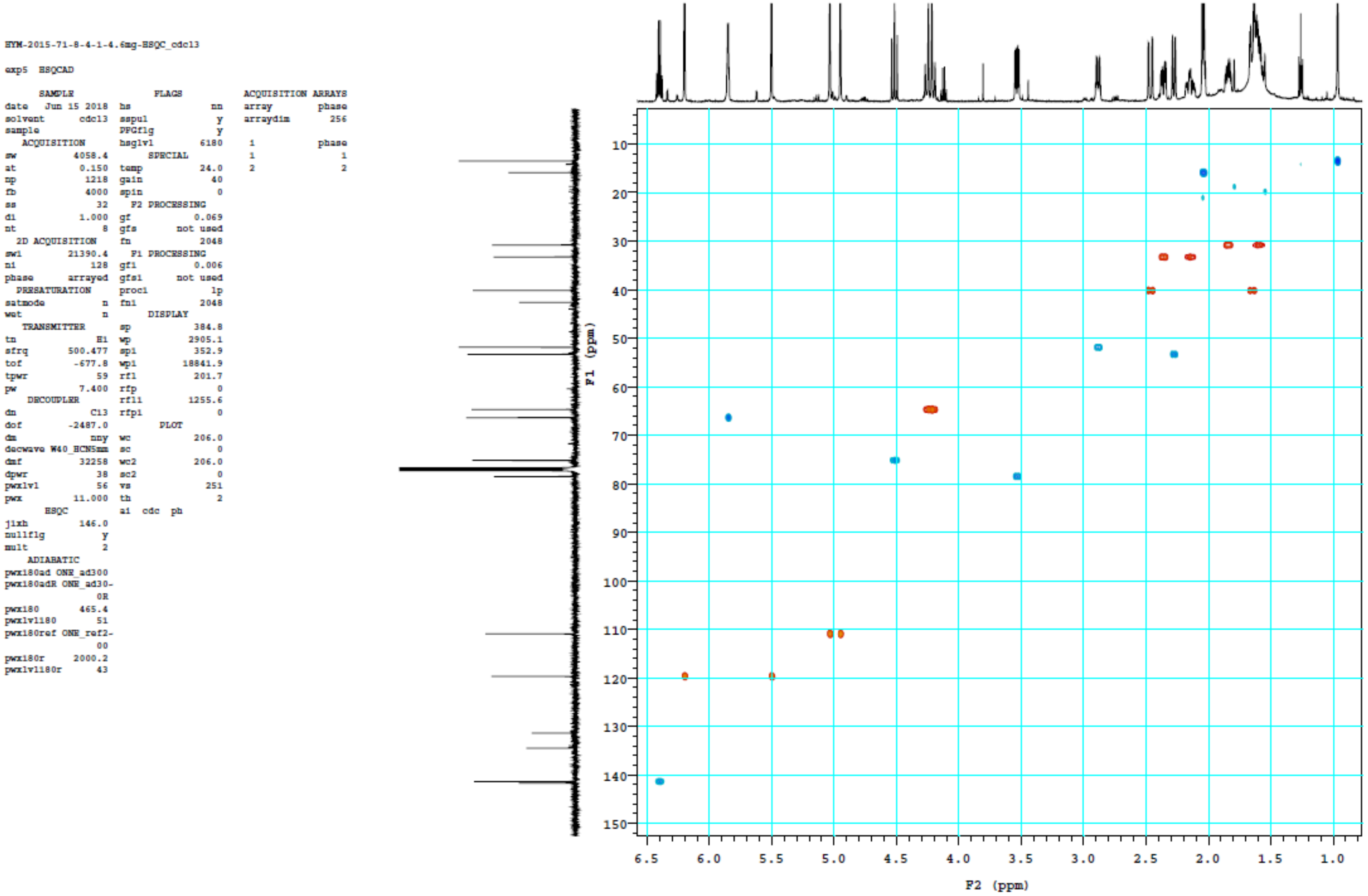

**Figure S47.** HMBC spectrum of **45** (measured in $CDCl_3$, 500 MHz).

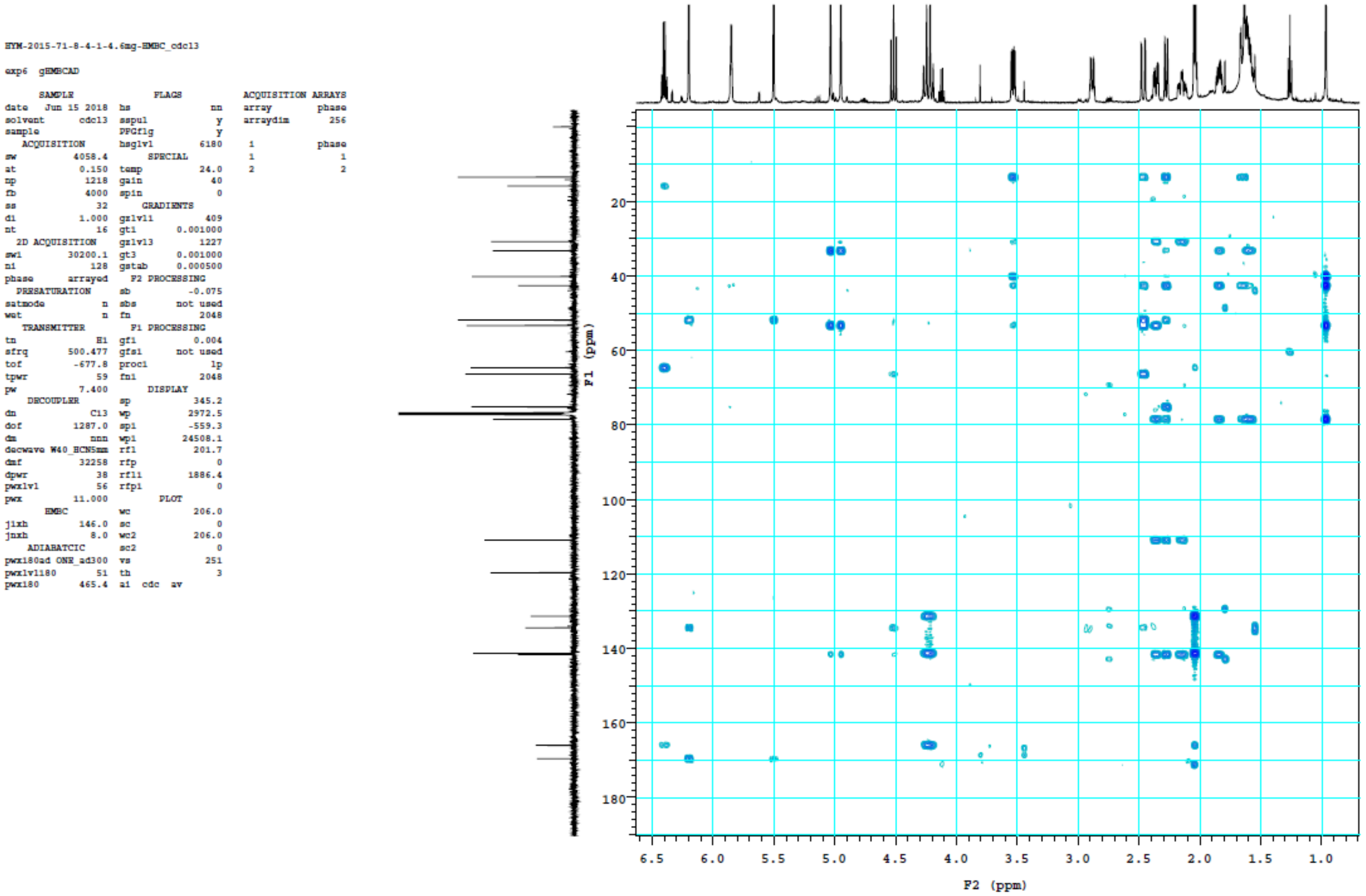

**Figure S48.** NOESY spectrum of **45** (measured in $CDCl_3$, 500 MHz).

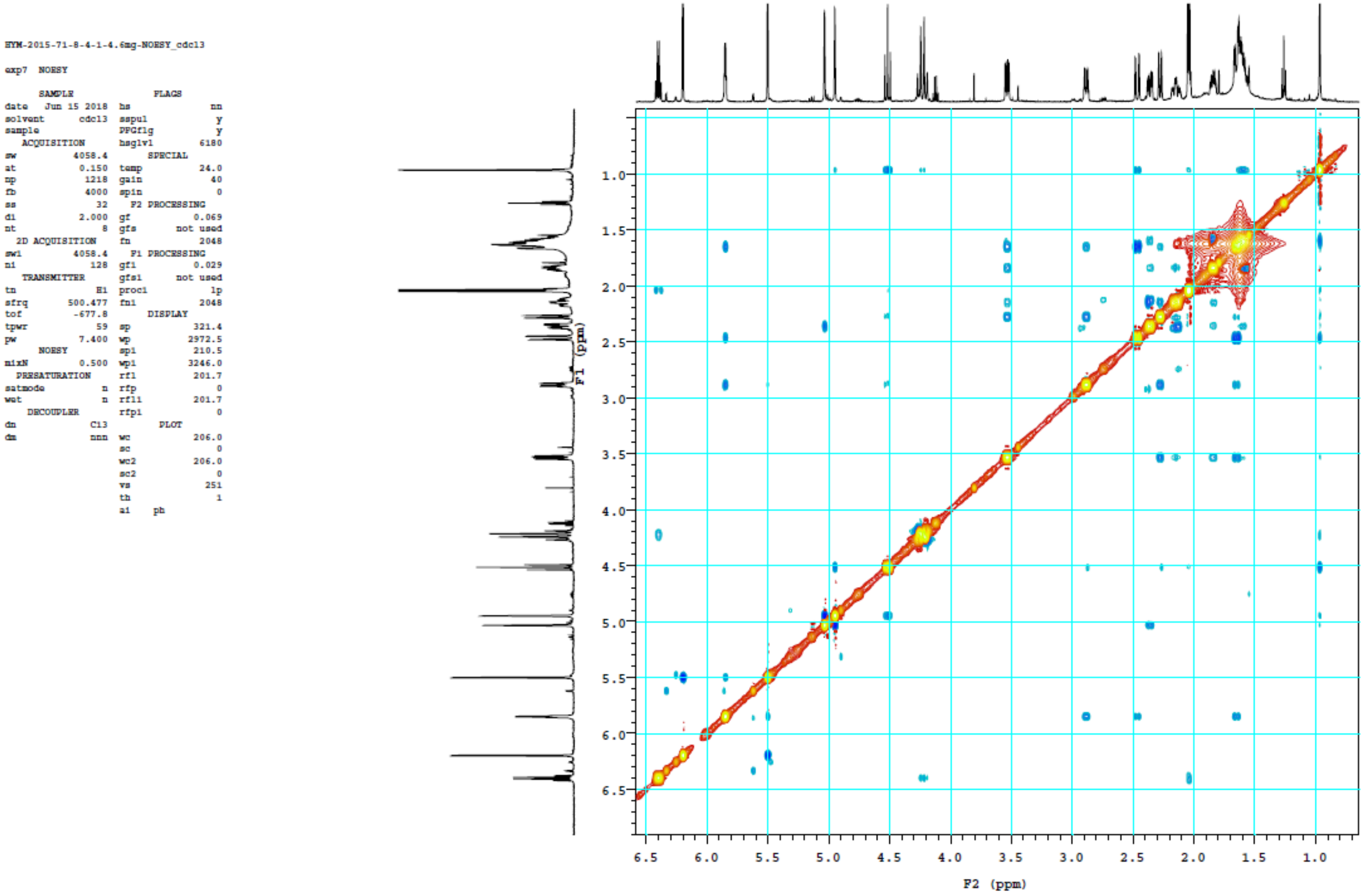

**Figure S49.** $^{1}$H NMR spectrum of **50** (measured in $CDCl_3$, 500 MHz).

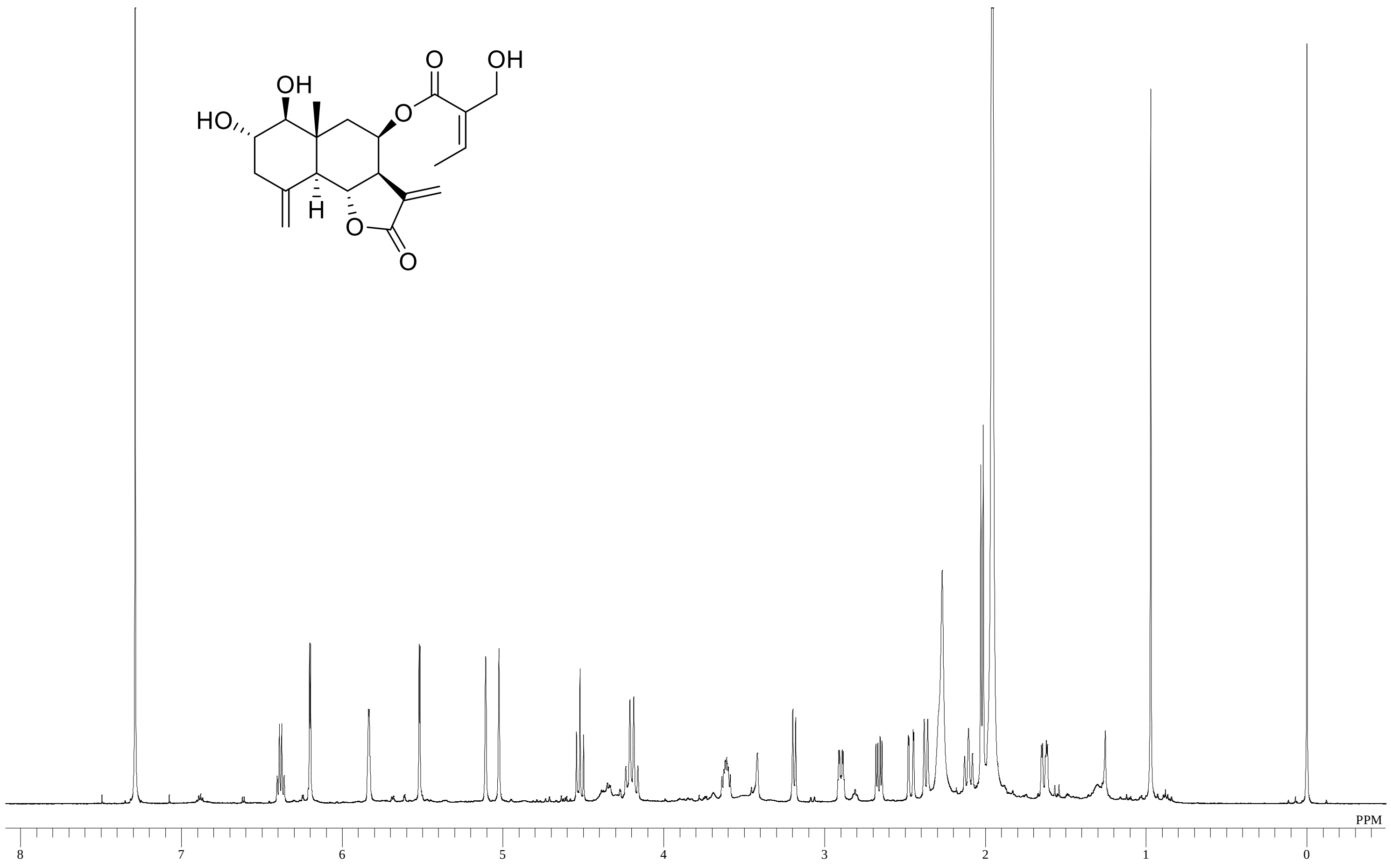

**Figure S50.** $^{13}$C NMR spectrum of **50** (measured in $CDCl_3$, 126 MHz).

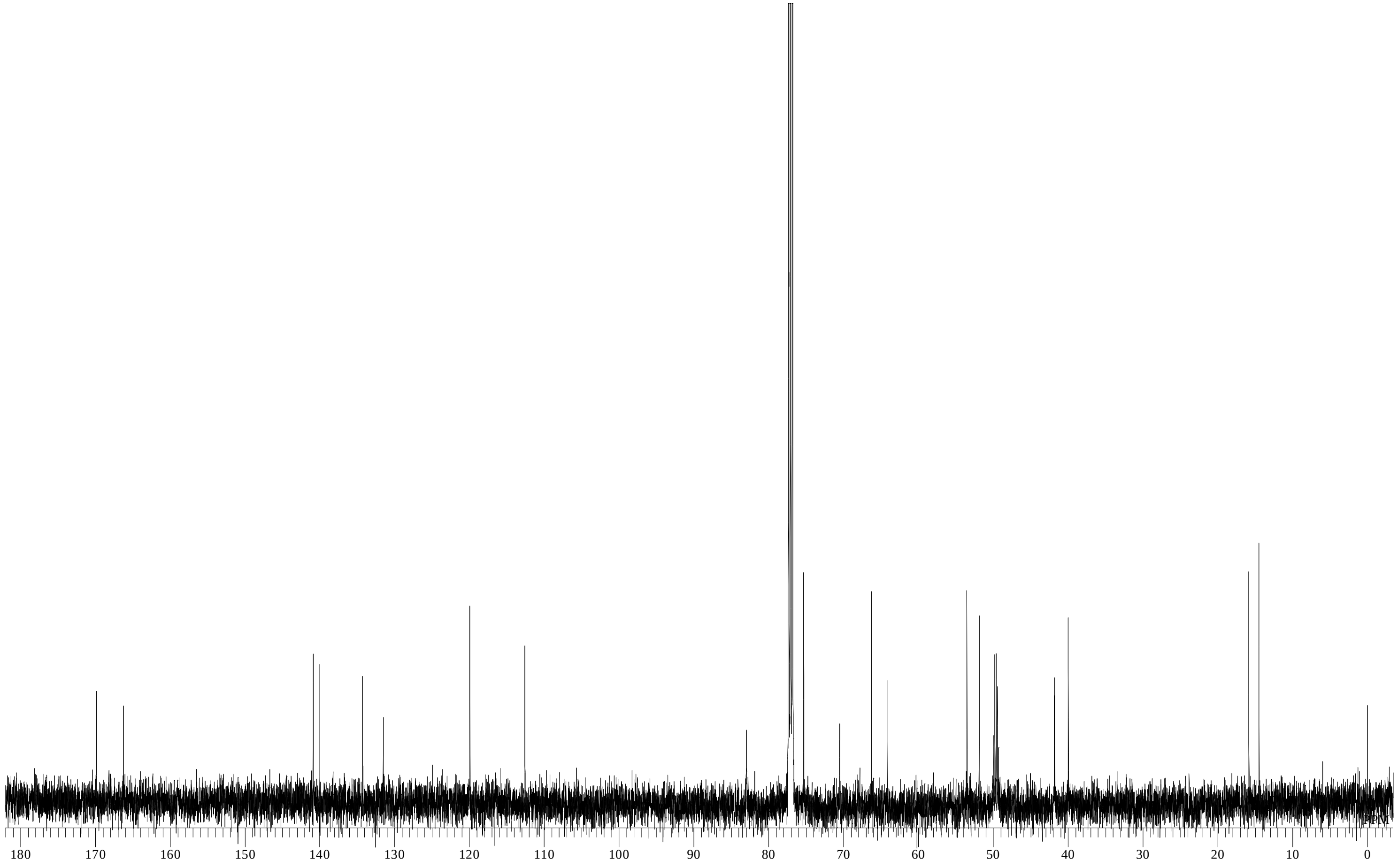

**Figure S51.** $^1H$-$^1H$ COSY spectrum of **50** (measured in $CDCl_3$, 500 MHz).

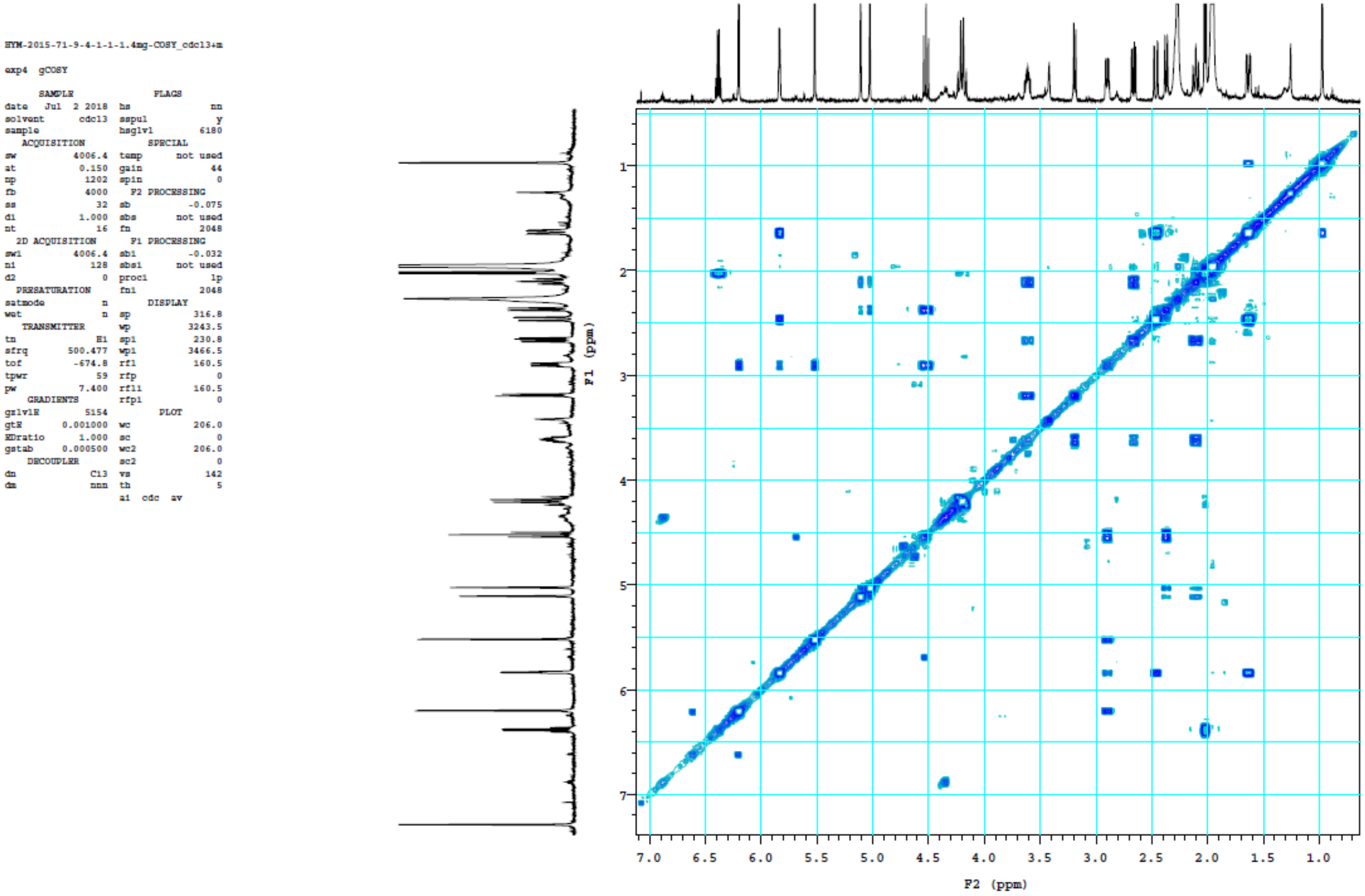

**Figure S52.** HSQC spectrum of **50** (measured in $CDCl_3$, 500 MHz).

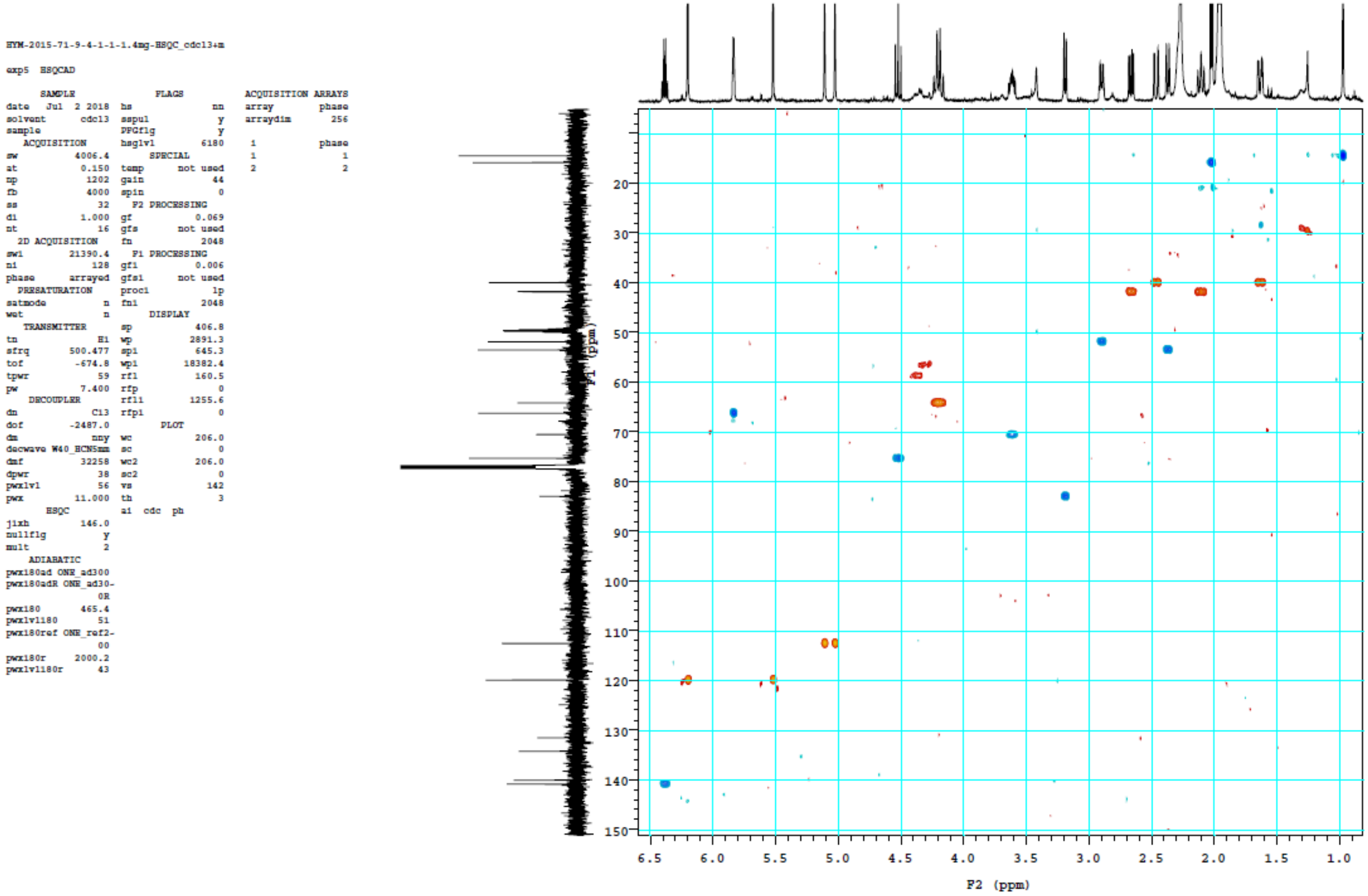

**Figure S53.** HMBC spectrum of **50** (measured in $CDCl_3$, 500 MHz).

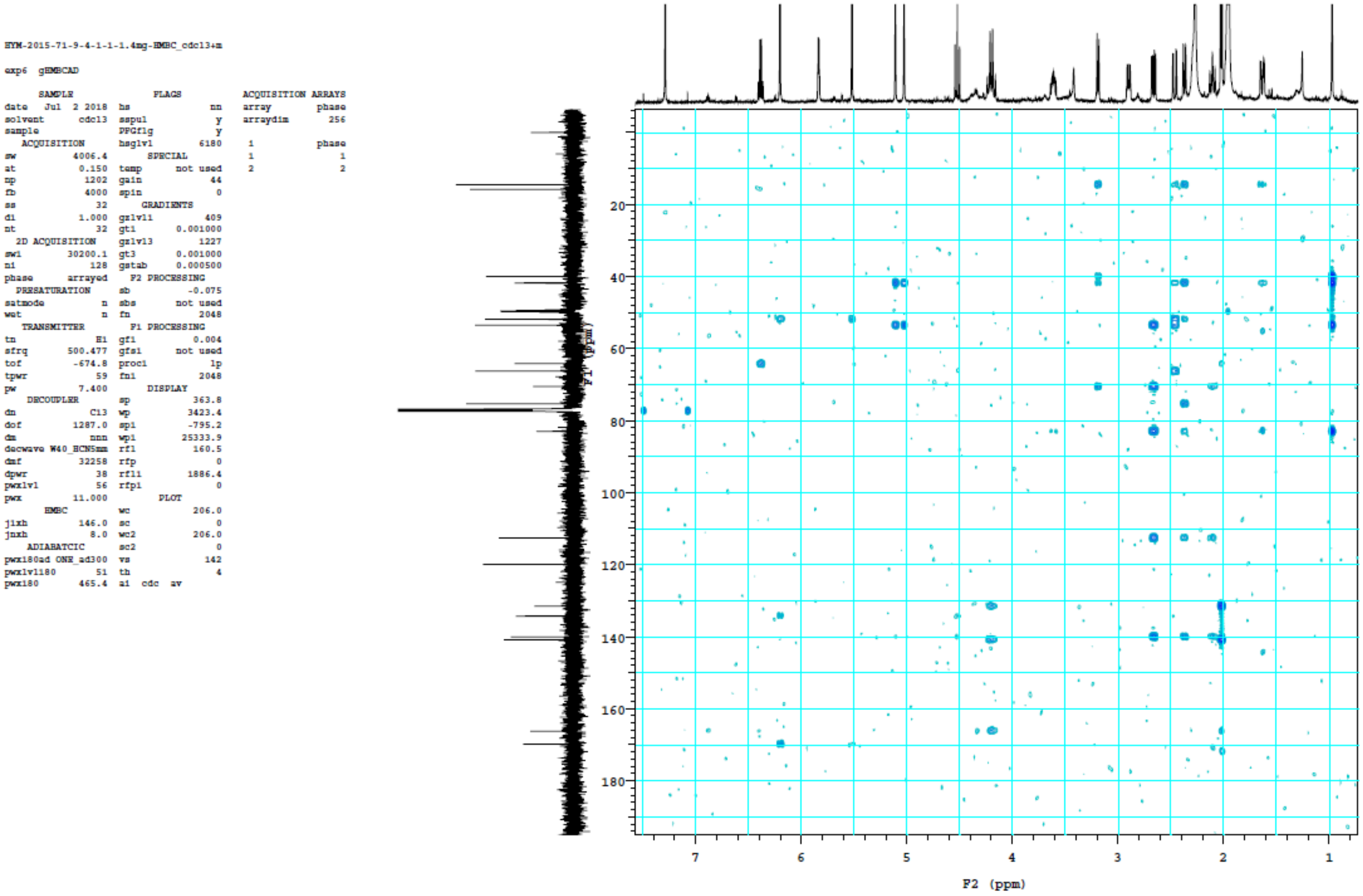

**Figure S54.** NOESY spectrum of **50** (measured in $CDCl_3$, 500 MHz).

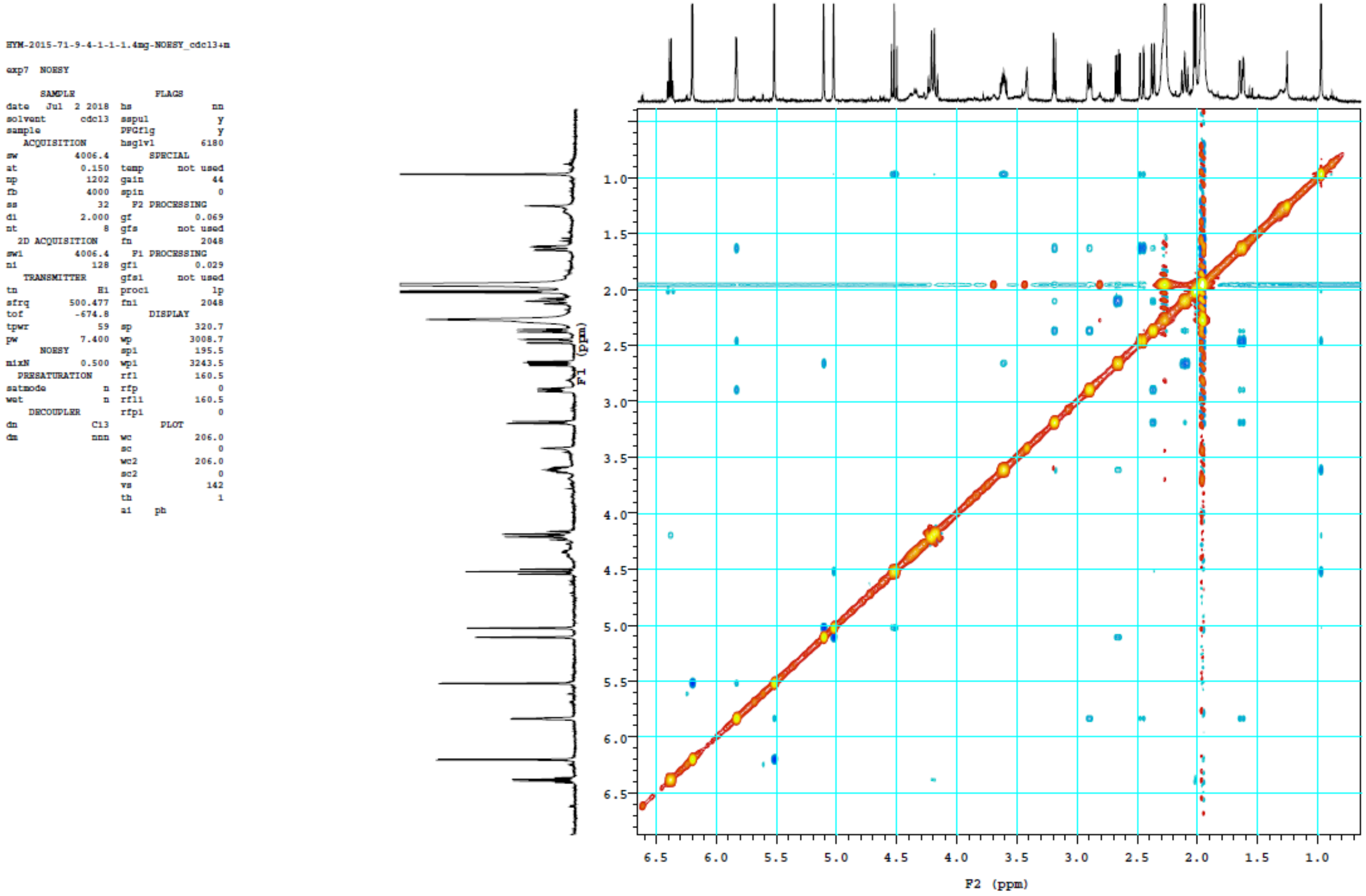

**Figure S55.** $^{1}$H NMR spectrum of **51** (measured in $CDCl_3$, 500 MHz).

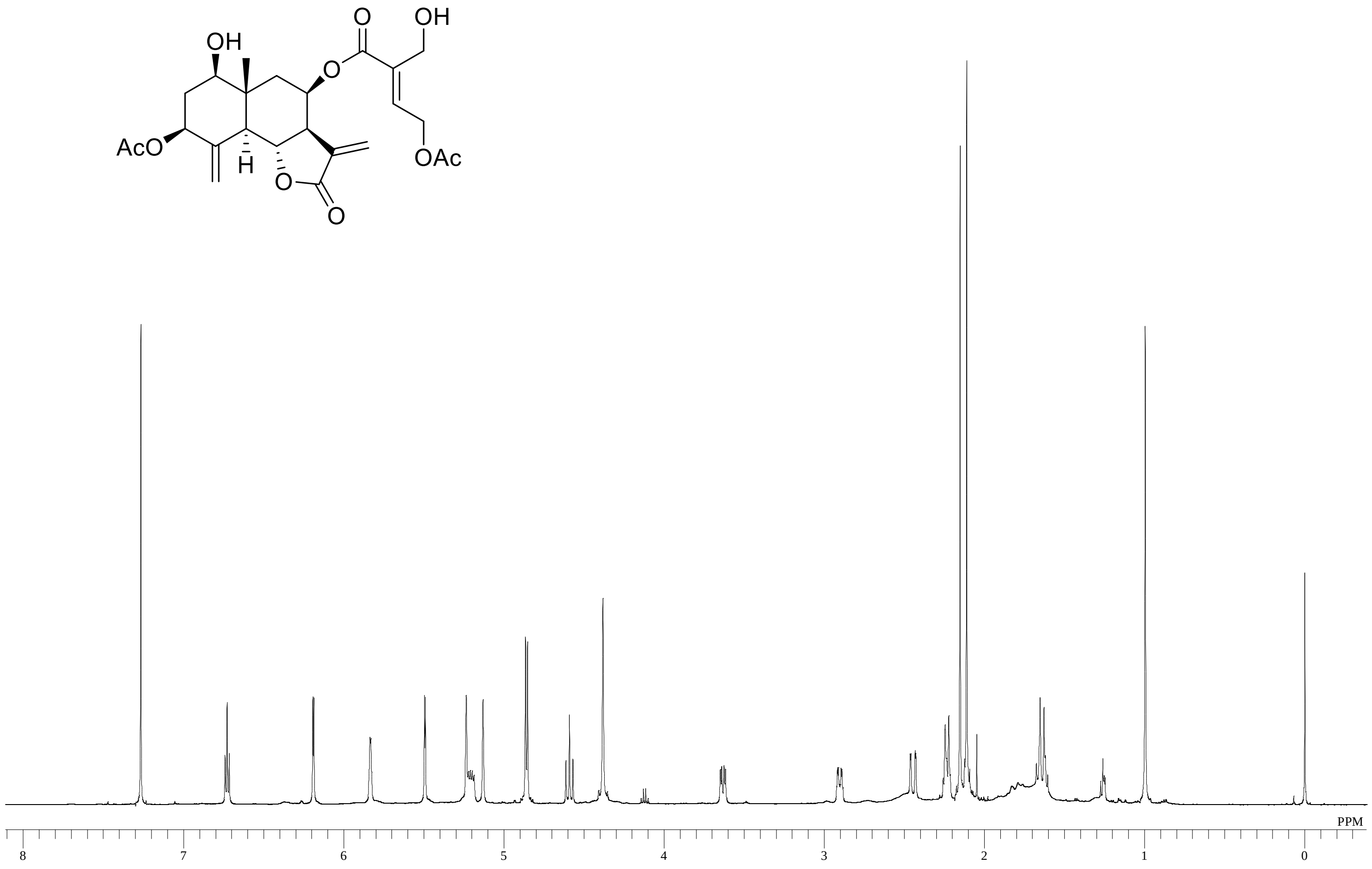

**Figure S56.** $^{13}C$ NMR spectrum of **51** (measured in $CDCl_3$, 126 MHz).

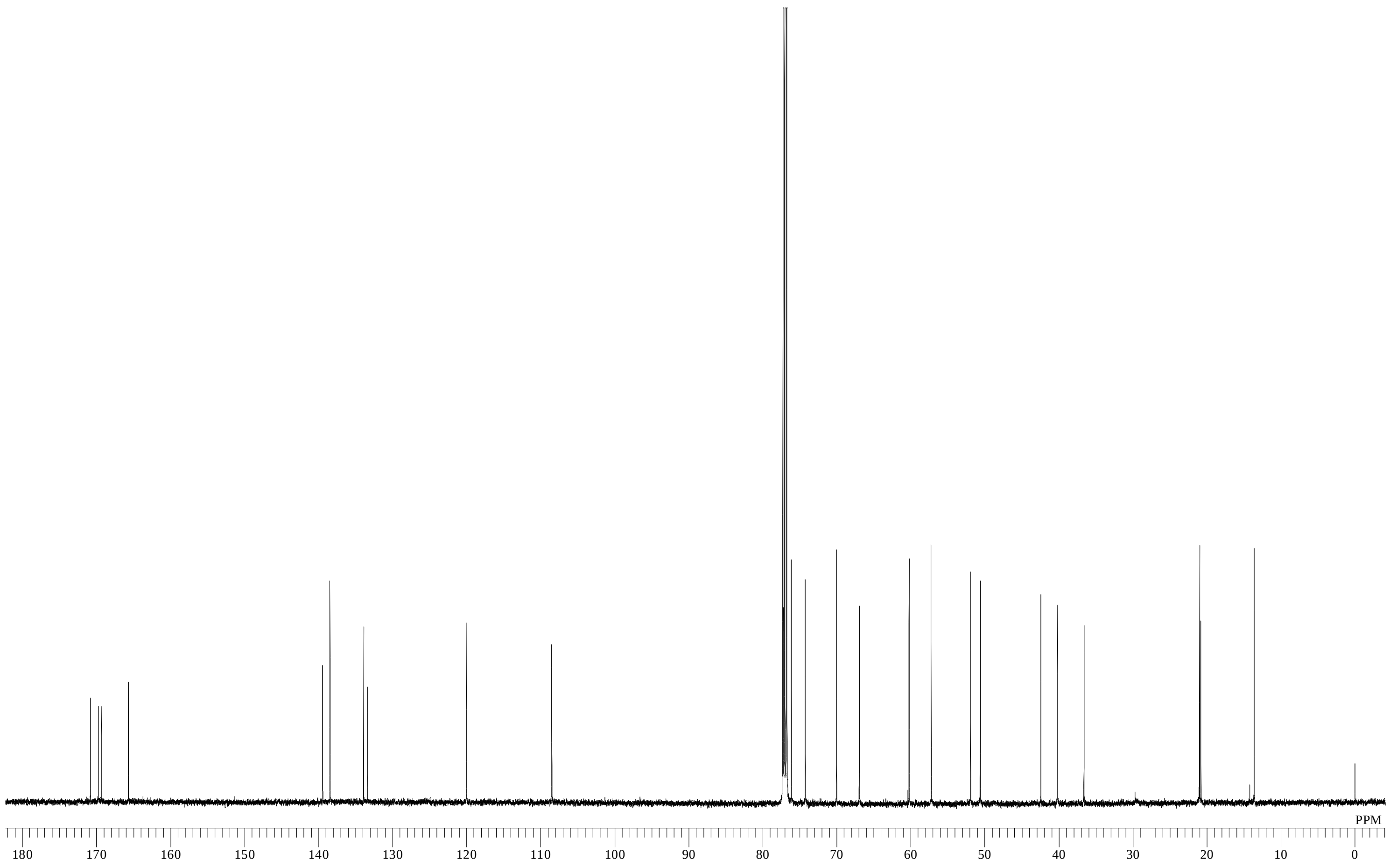

**Figure S57.** $^{1}H$-$^{1}H$ COSY spectrum of **51** (measured in $CDCl_3$, 500 MHz).

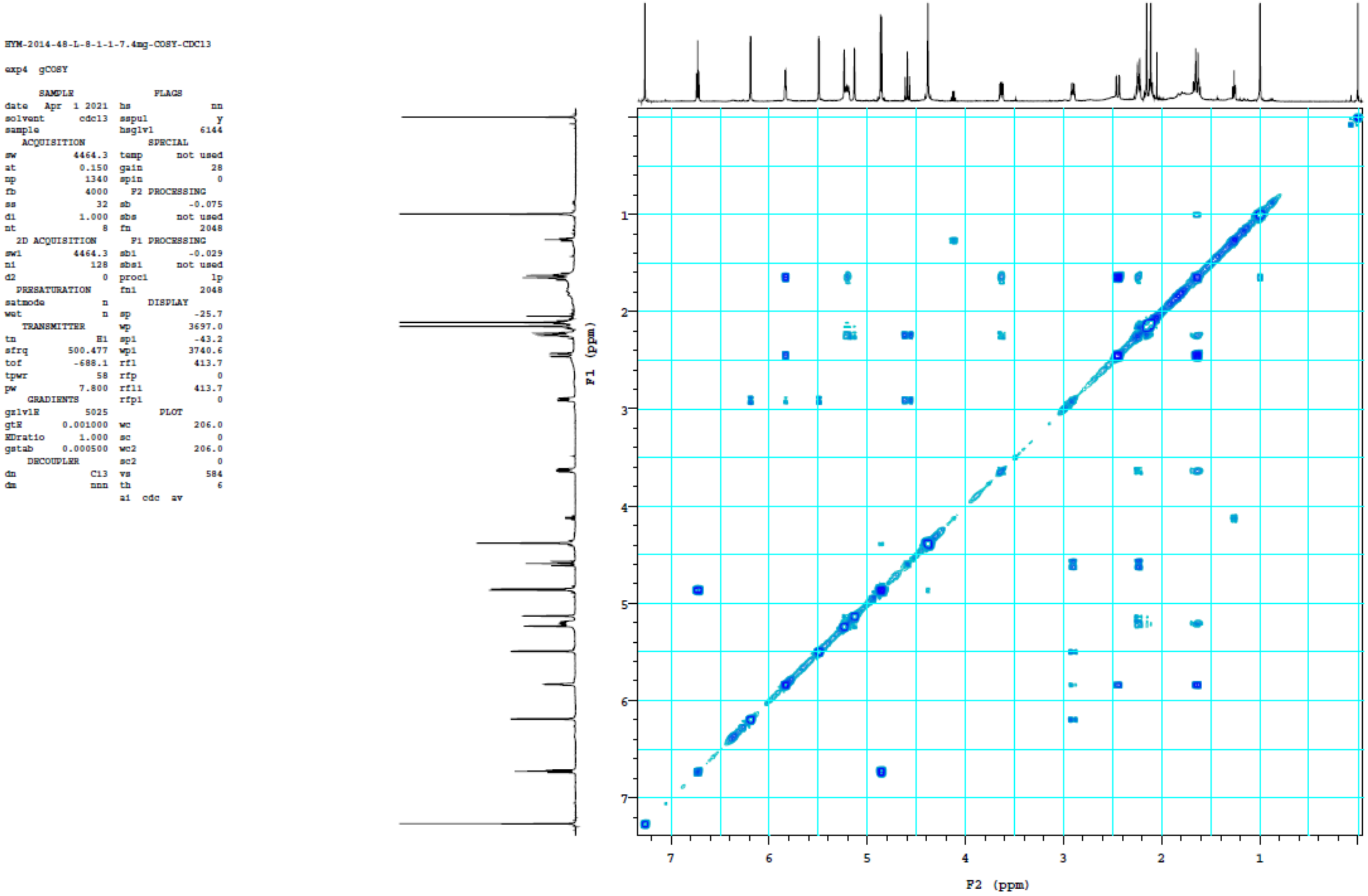

**Figure S58.** HSQC spectrum of **51** (measured in $CDCl_3$, 500 MHz).

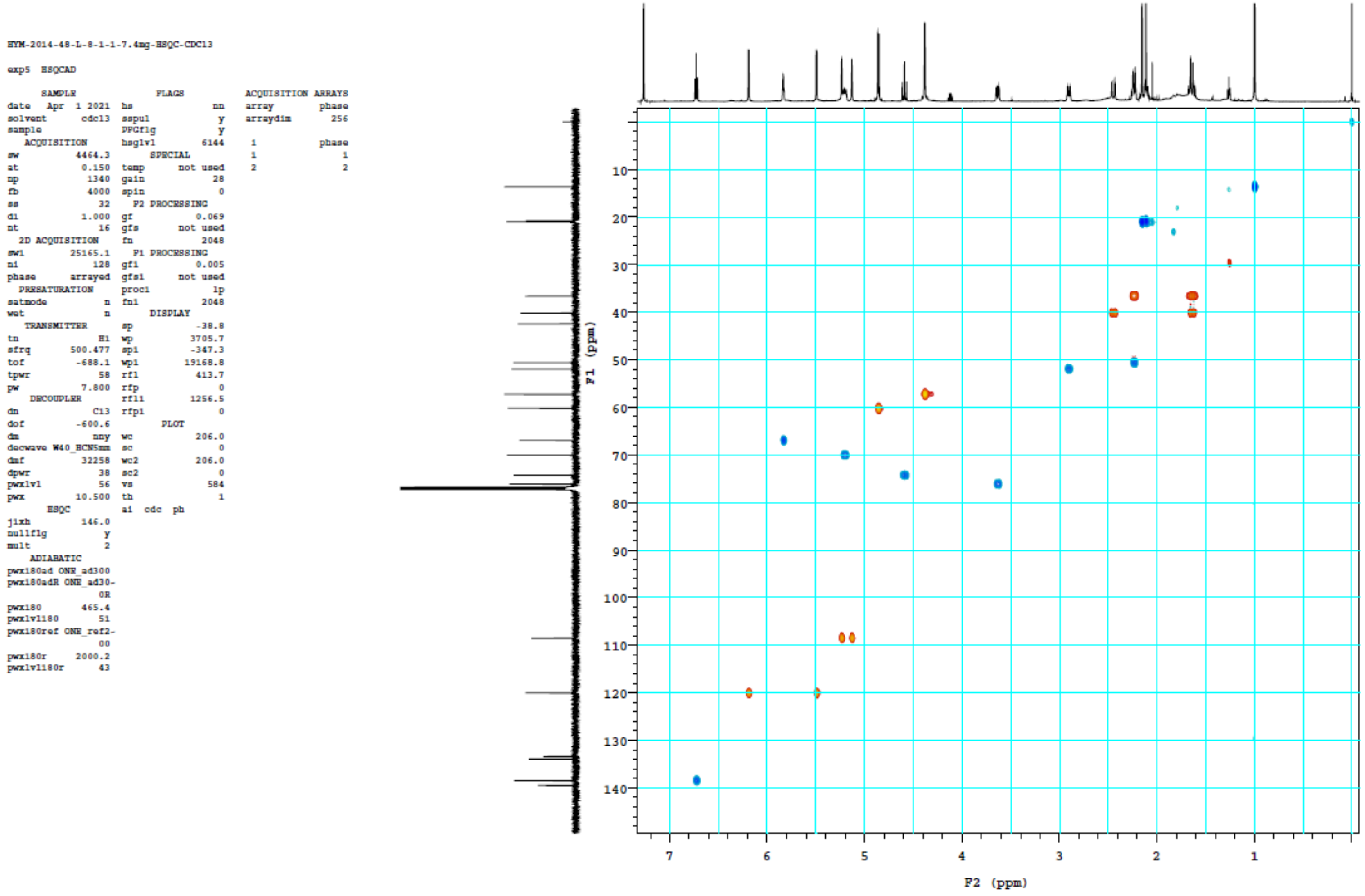

**Figure S59.** HMBC spectrum of **51** (measured in $CDCl_3$, 500 MHz).

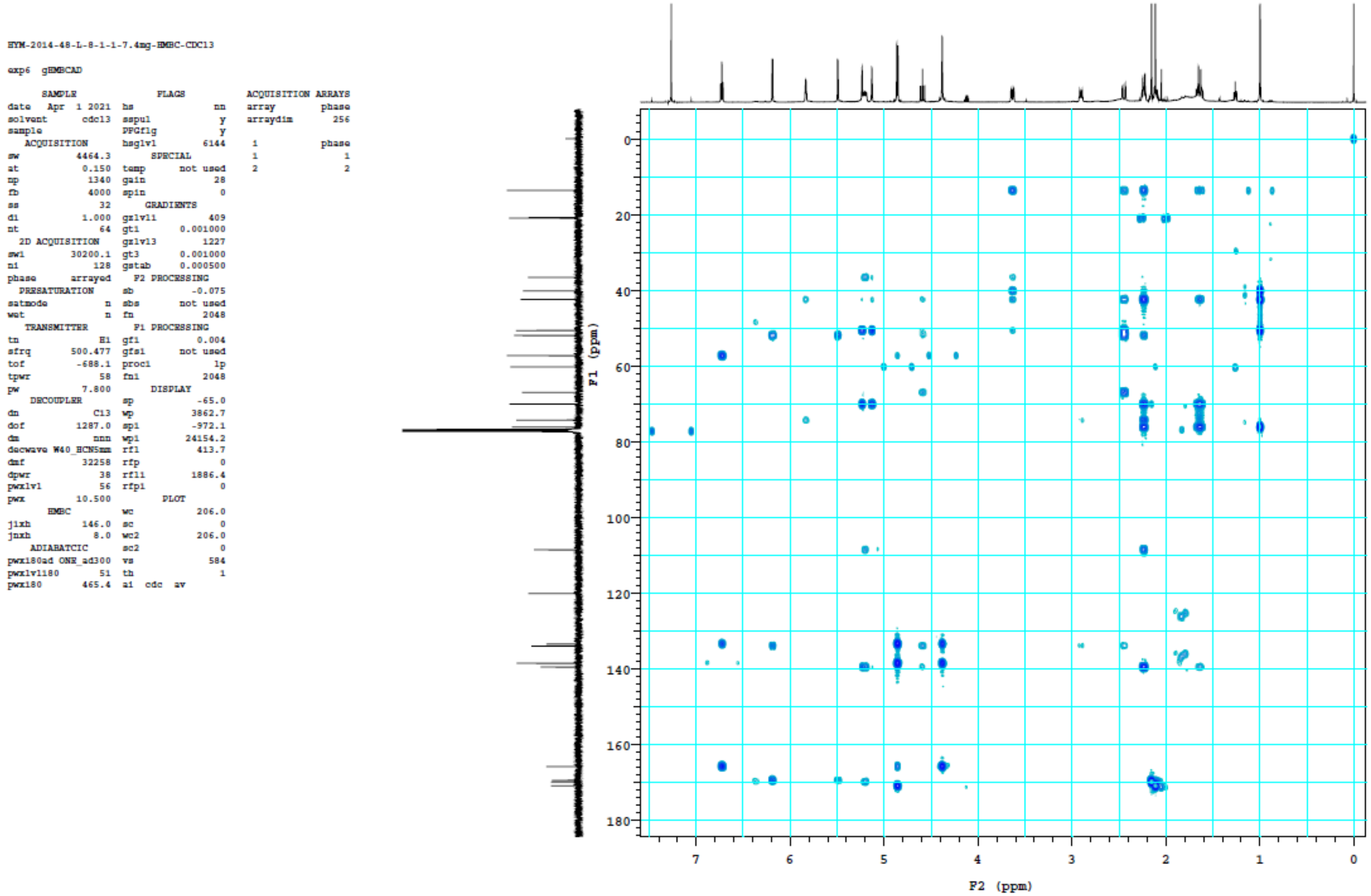

**Figure S60.** NOESY spectrum of **51** (measured in $CDCl_3$, 500 MHz).

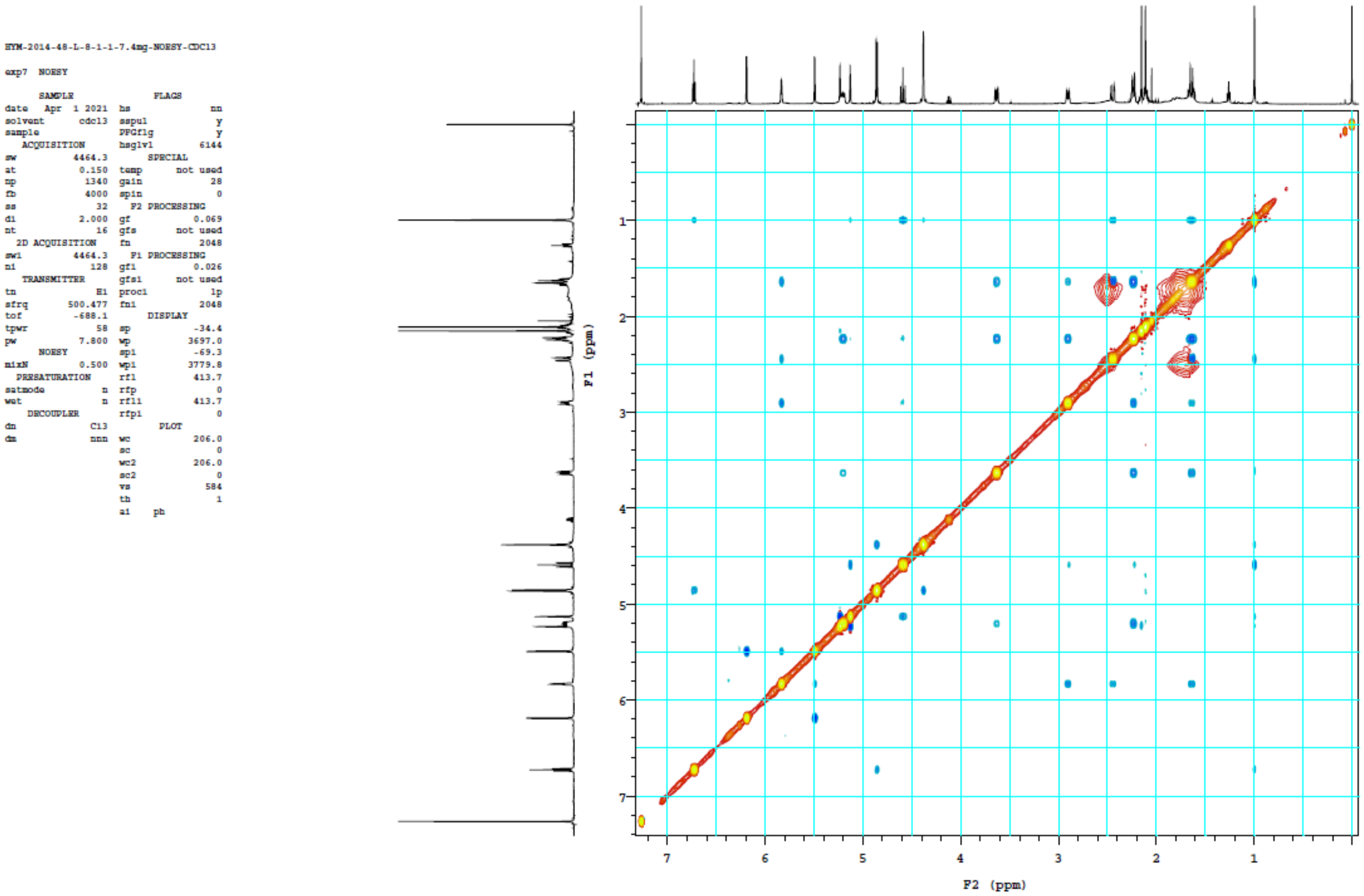

**Figure S61.** $^{1}$H NMR spectrum of **53** (measured in $CDCl_3$, 500 MHz).

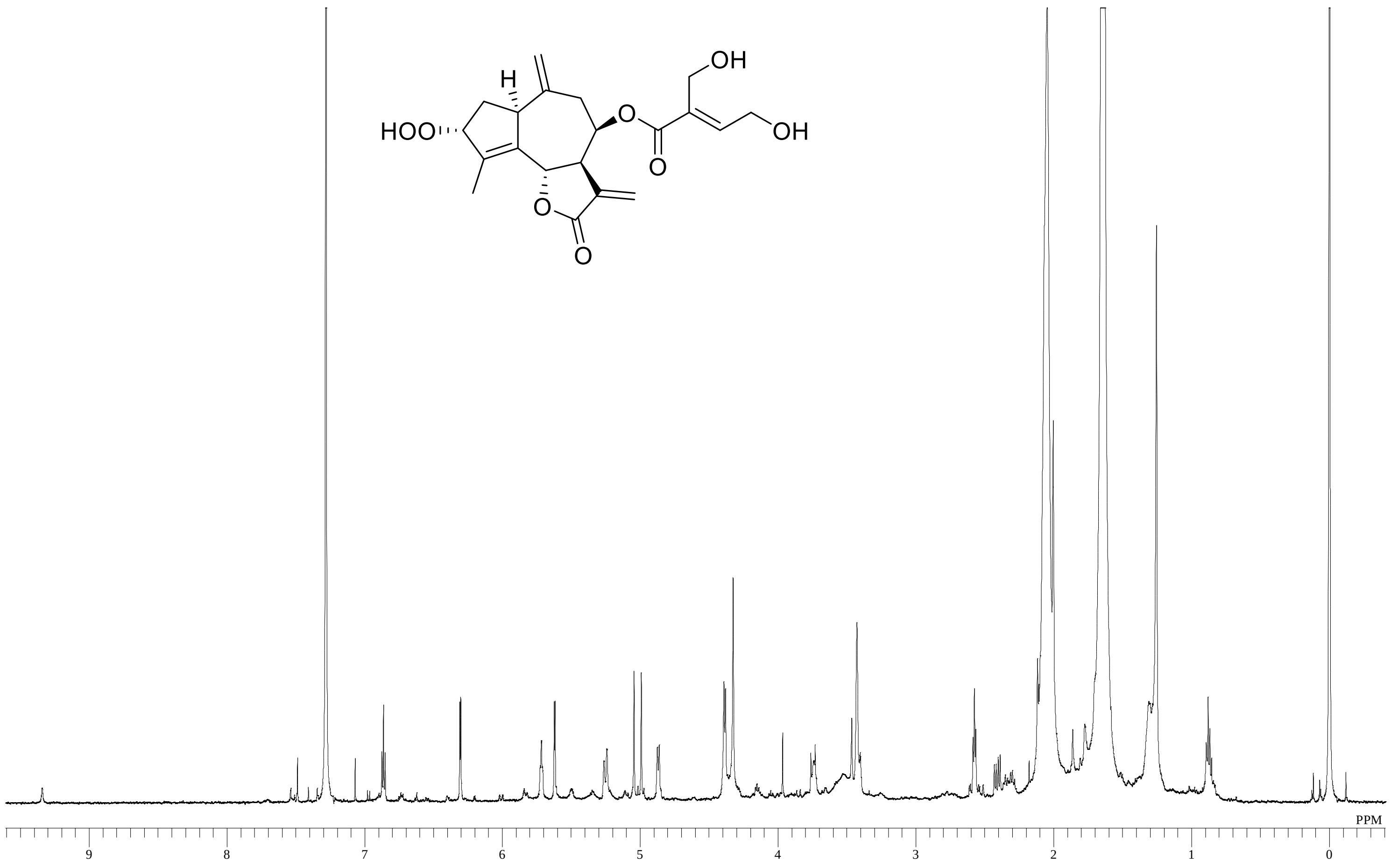

**Figure S62.** $^{13}$C NMR spectrum of **53** (measured in $CDCl_3$, 126 MHz).

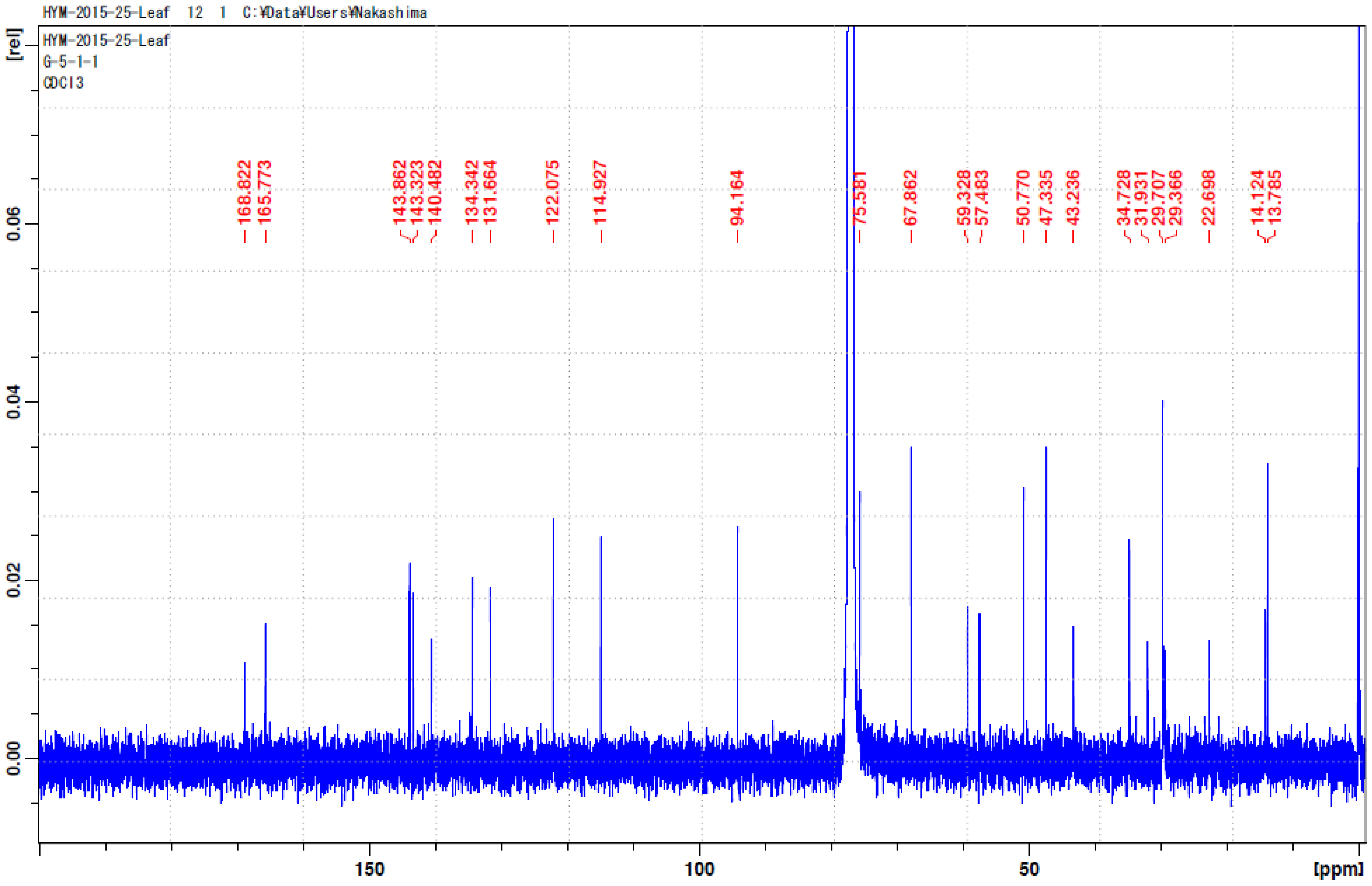

**Figure S63.** $^1H$-$^1H$ COSY spectrum of **53** (measured in $CDCl_3$, 500 MHz).

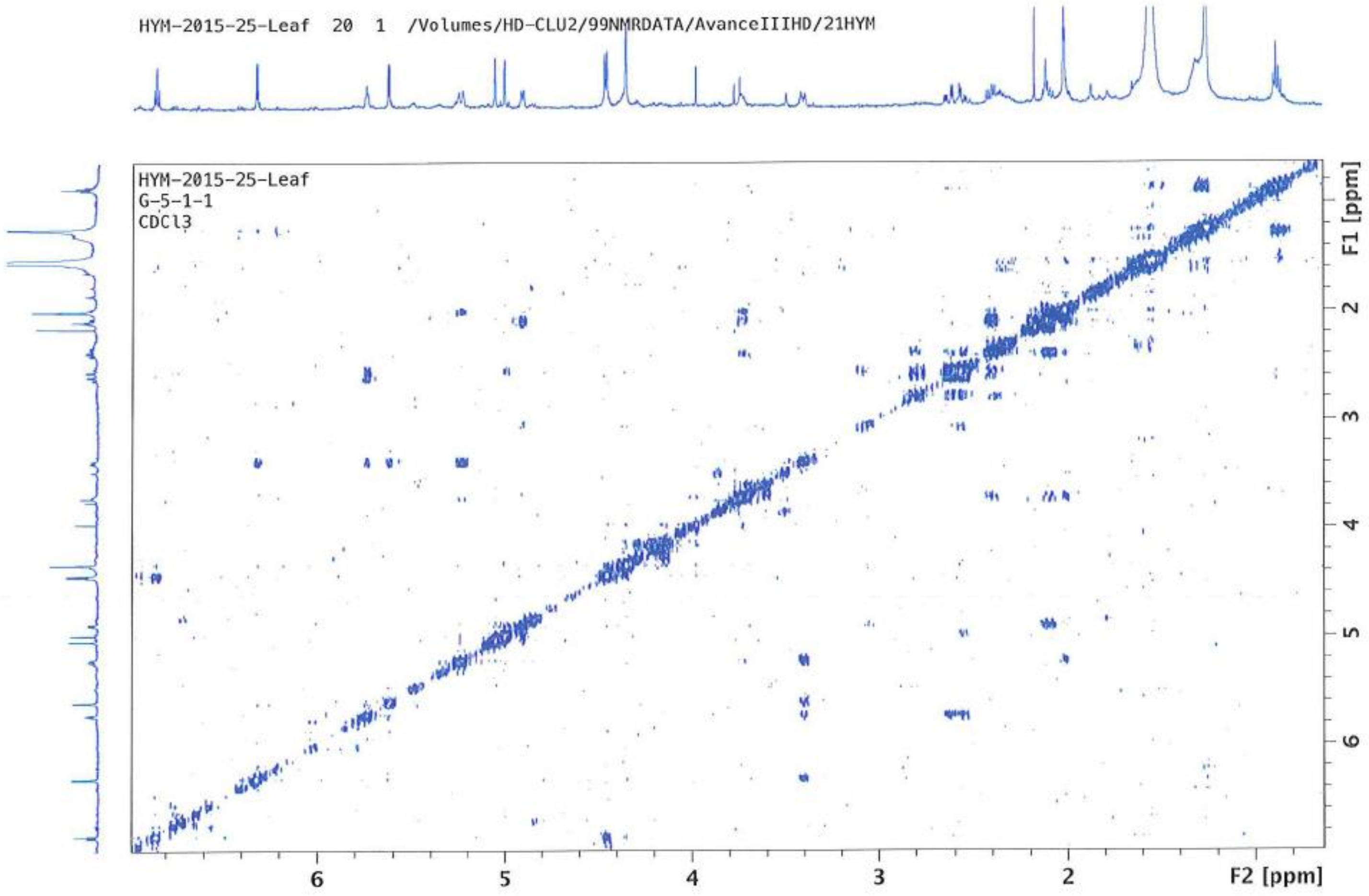

**Figure S64.** HSQC spectrum of **53** (measured in $CDCl_3$, 500 MHz).

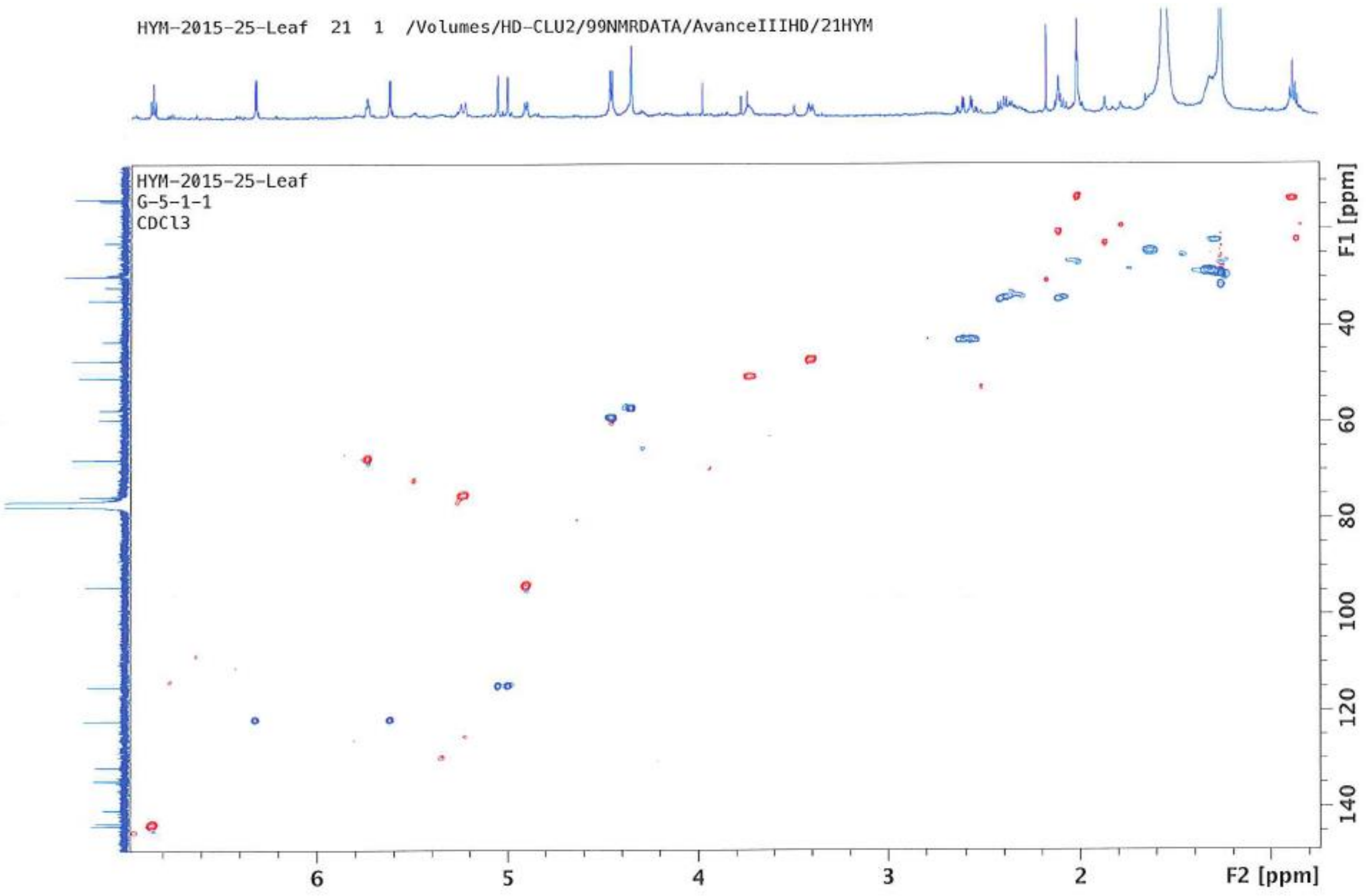

**Figure S65.** HMBC spectrum of **53** (measured in $CDCl_3$, 500 MHz).

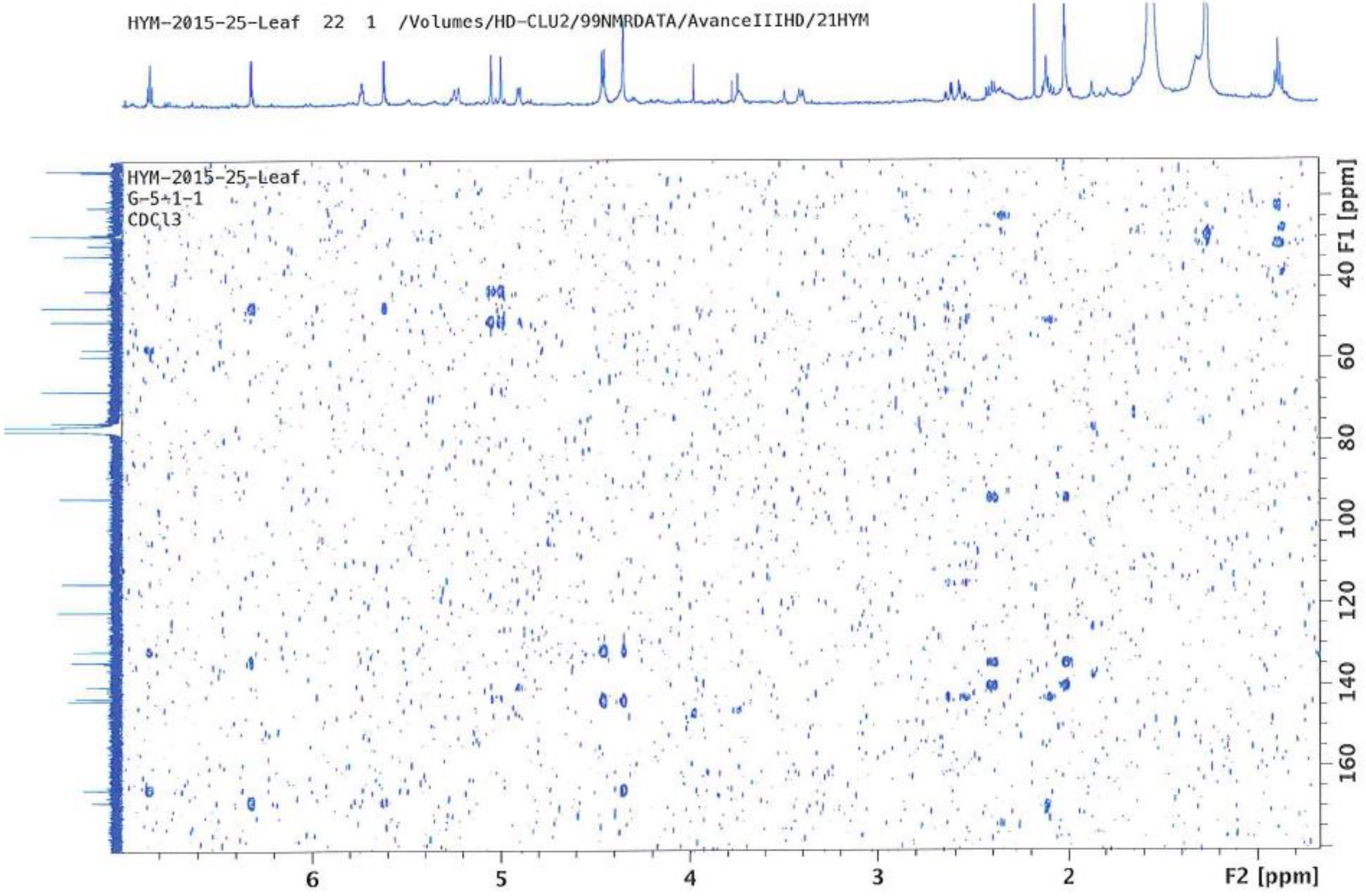

**Figure S66.** NOESY spectrum of **53** (measured in $CDCl_3$, 500 MHz).

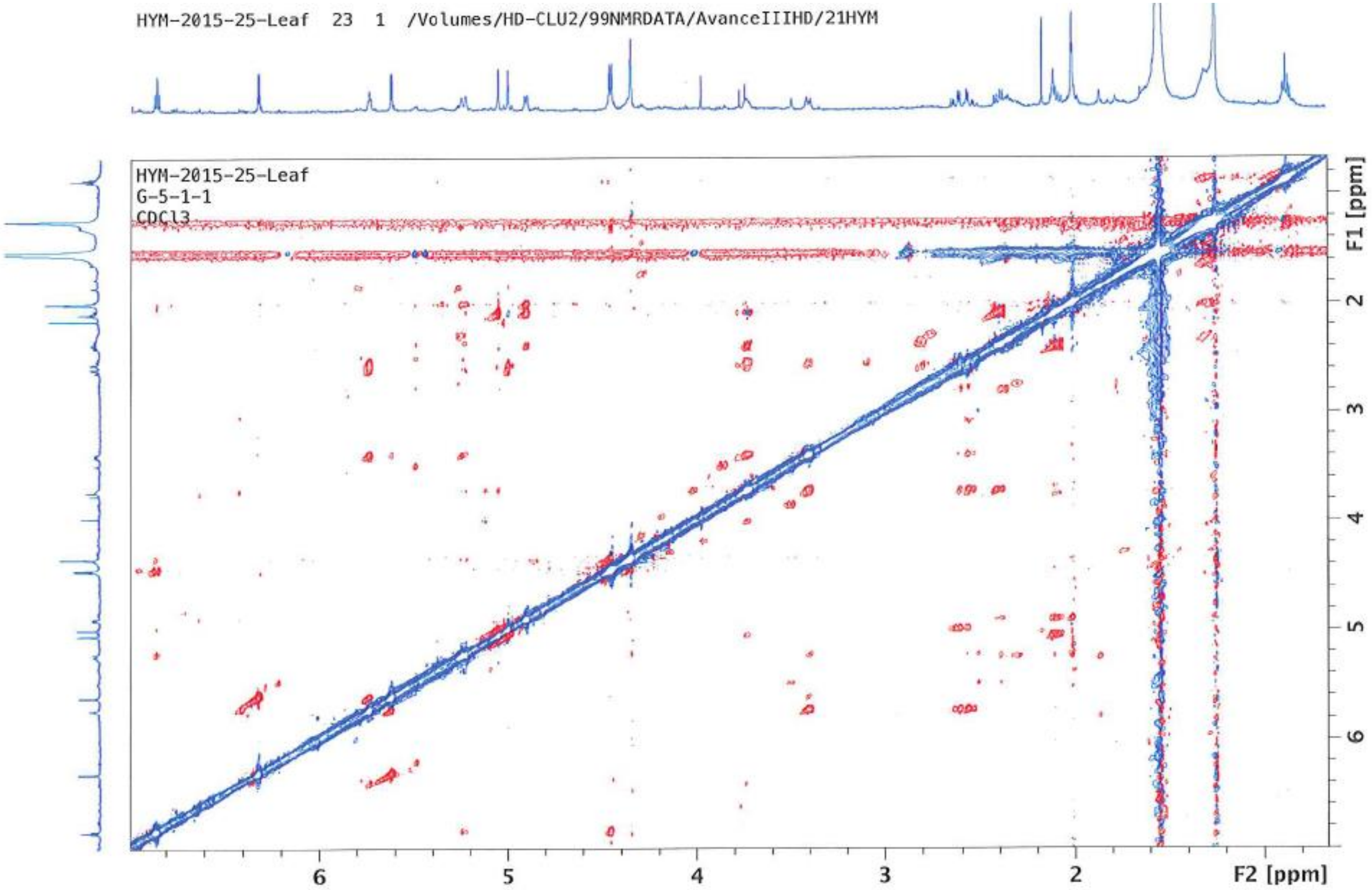

**Figure S67.** $^1$H NMR spectrum of **54** (measured in $CDCl_3$, 500 MHz).

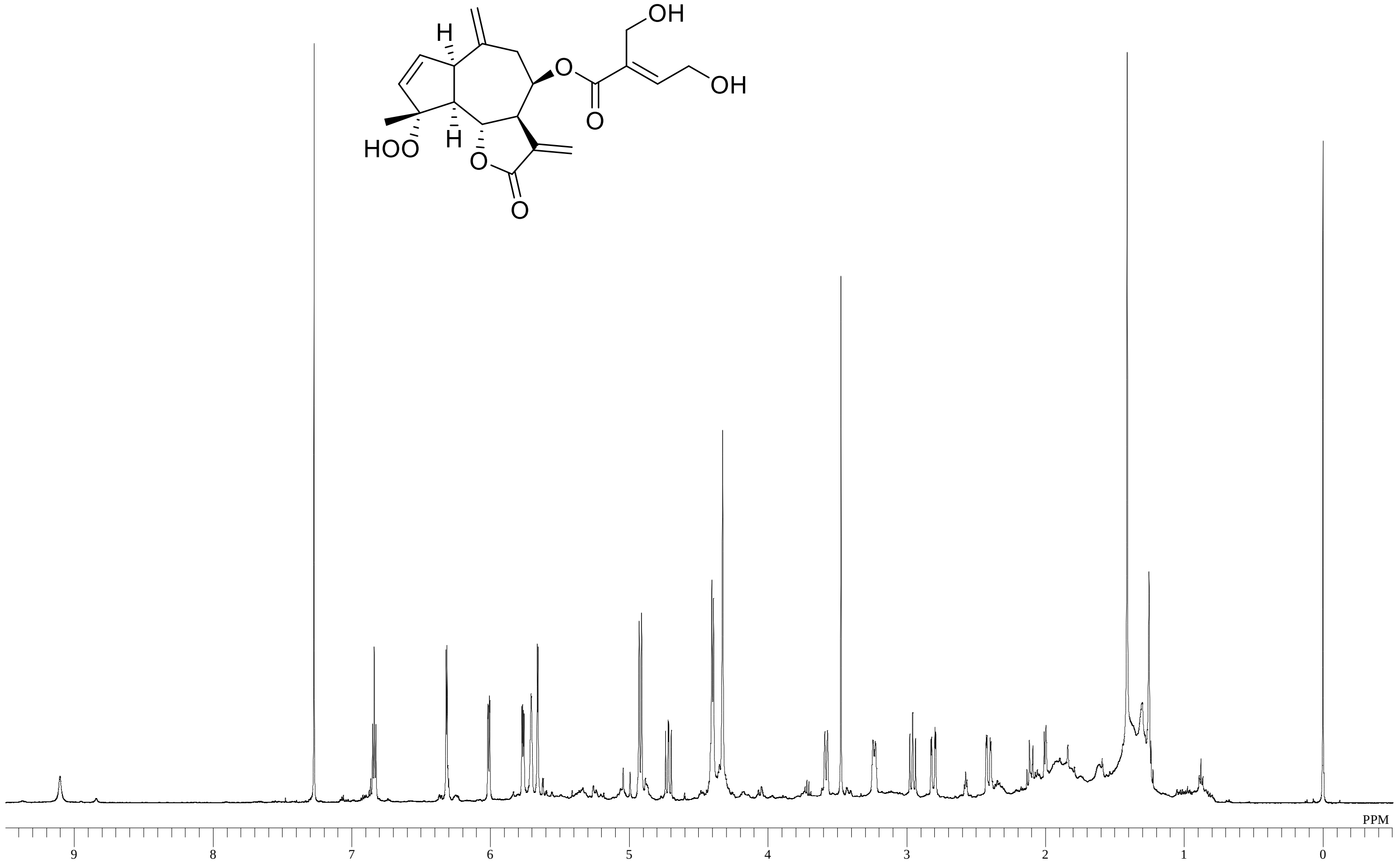

**Figure S68.** $^{13}$C NMR spectrum of **54** (measured in $CDCl_3$, 126 MHz).

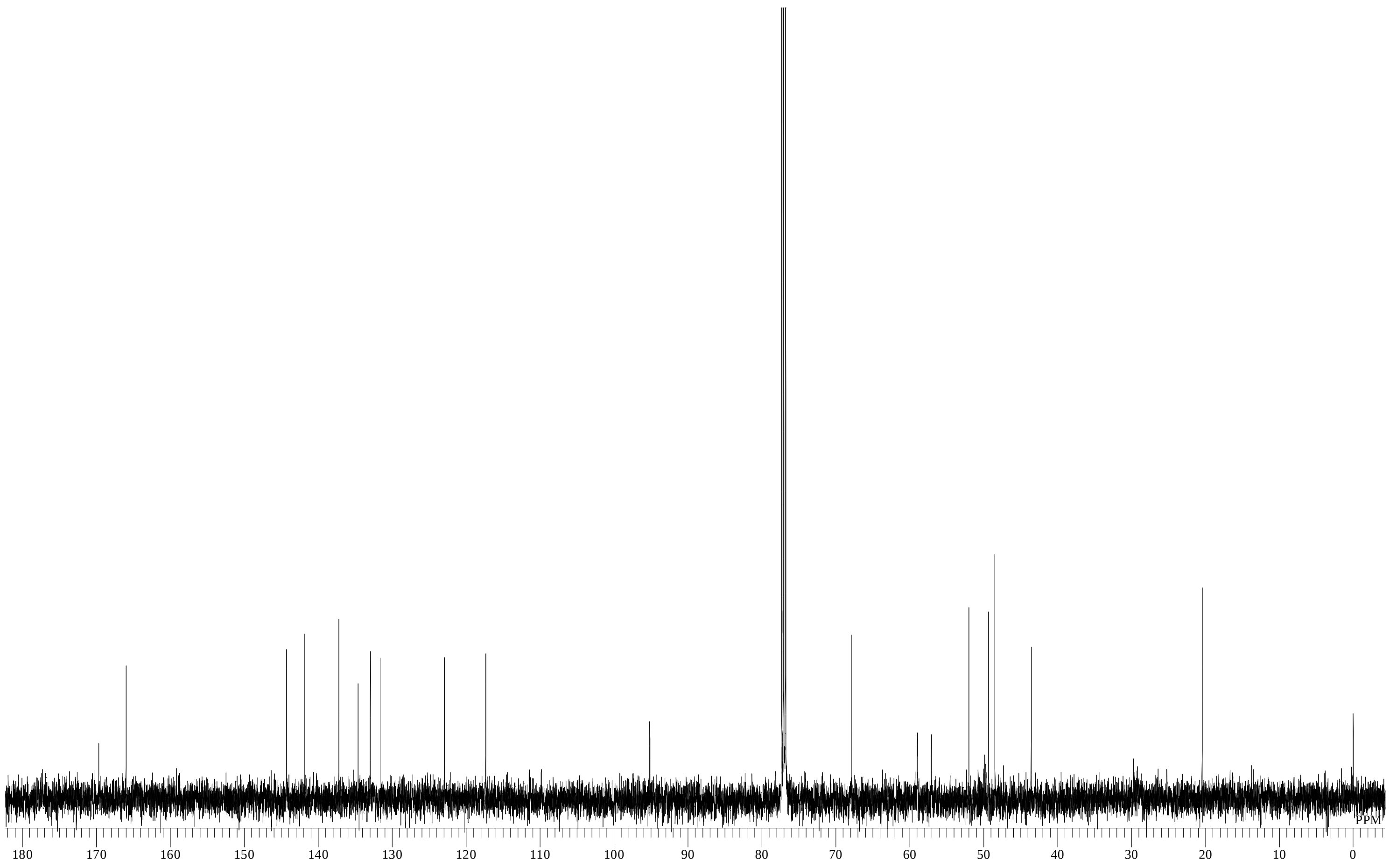

**Figure S69.** $^1$H-$^1$H COSY spectrum of **54** (measured in $CDCl_3$, 500 MHz).

HYM-G-6-2-6.8mg-COSY_cdcl3

exp6 gCOSY

| | | | |
|---|---|---|---|
| SAMPLE | | FLAGS | |
| date | Sep 27 2017 | hs | nn |
| solvent | cdcl3 | sspul | y |
| sample | | hsglvl | 6180 |
| ACQUISITION | | SPECIAL | |
| sw | 5165.3 | temp | 24.0 |
| at | 0.150 | gain | 36 |
| np | 1550 | spin | 0 |
| fb | 4000 | F2 PROCESSING | |
| ss | 32 | sb | -0.075 |
| d1 | 1.000 | sbs | not used |
| nt | 4 | fn | 2048 |
| 2D ACQUISITION | | F1 PROCESSING | |
| sw1 | 5165.3 | sb1 | -0.025 |
| ni | 128 | sbs1 | not used |
| d2 | 0 | proc1 | lp |
| PRESATURATION | | fn1 | 2048 |
| satmode | n | DISPLAY | |
| wet | n | sp | -96.0 |
| TRANSMITTER | | wp | 4252.3 |
| tn | H1 | sp1 | -86.0 |
| sfrq | 500.484 | wp1 | 4302.7 |
| tof | -178.0 | rfl | 257.5 |
| tpwr | 58 | rfp | 0 |
| pw | 7.900 | rfl1 | 257.5 |
| GRADIENTS | | rfp1 | 0 |
| gzlvlE | 5154 | PLOT | |
| gtE | 0.001000 | wc | 206.0 |
| EDratio | 1.000 | sc | 0 |
| gstab | 0.000500 | wc2 | 206.0 |
| DECOUPLER | | sc2 | 0 |
| dn | C13 | vs | 5465 |
| dm | nnn | th | 6 |
| | | ai cdc av | |

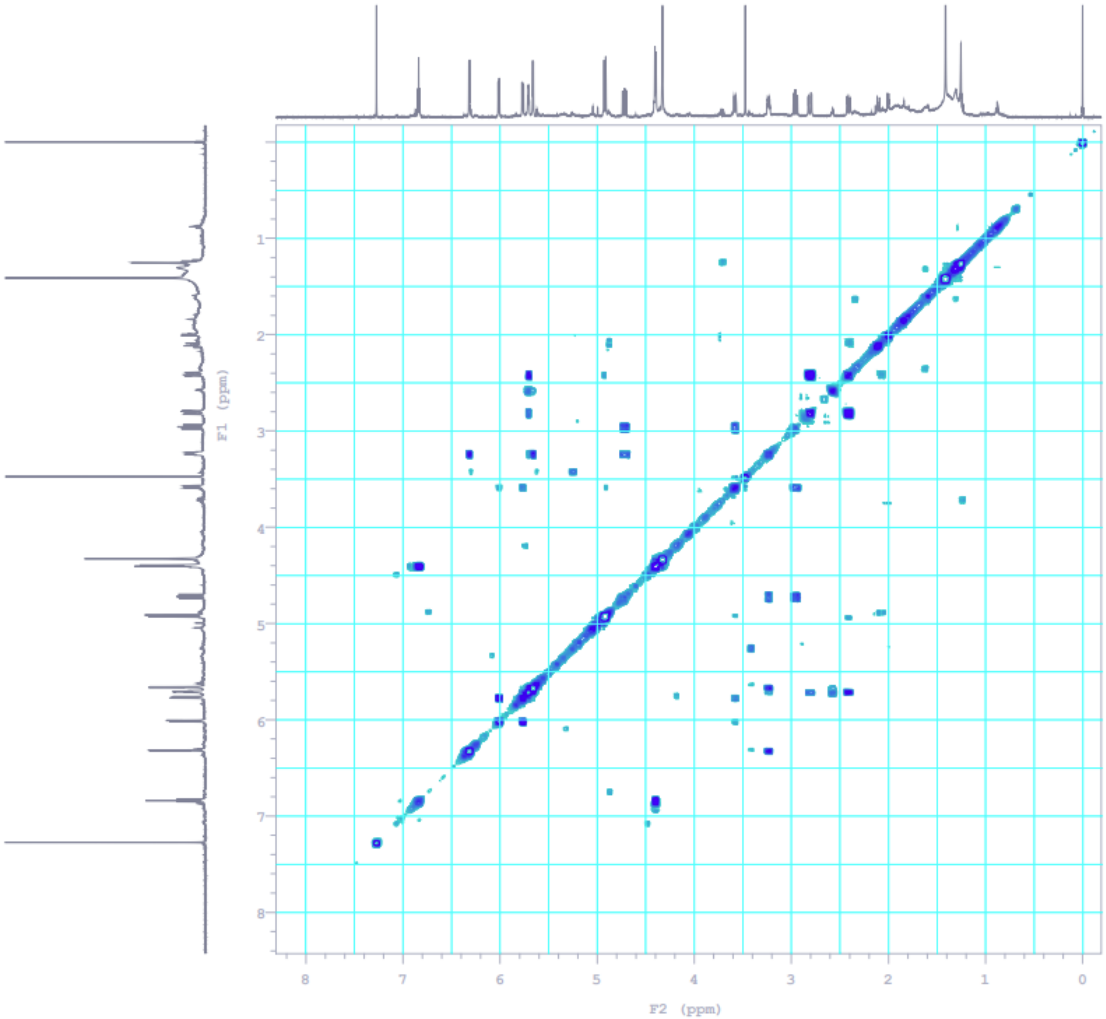

**Figure S70.** HSQC spectrum of **54** (measured in $CDCl_3$, 500 MHz).

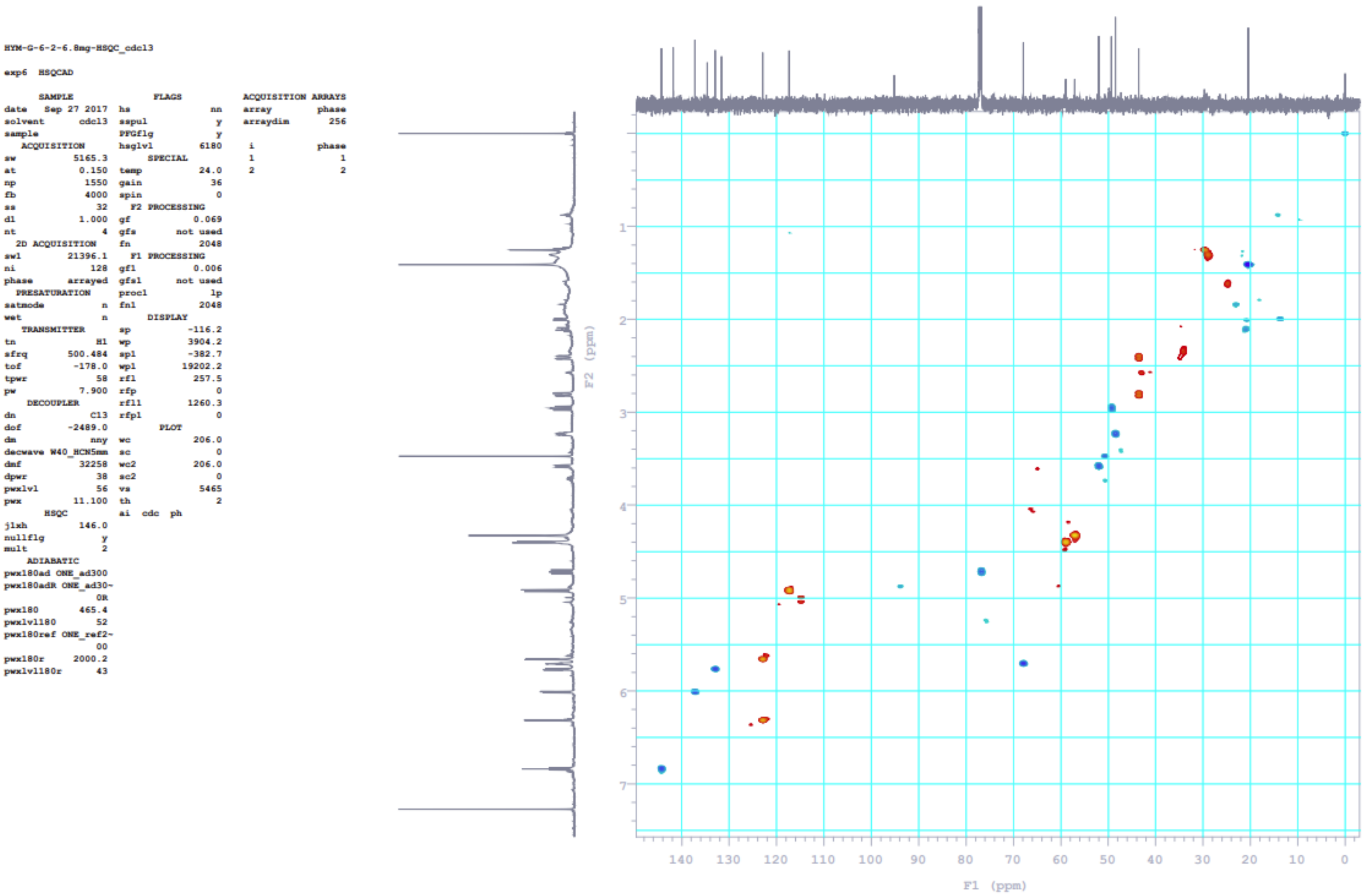

**Figure S71.** HMBC spectrum of **54** (measured in $CDCl_3$, 500 MHz).

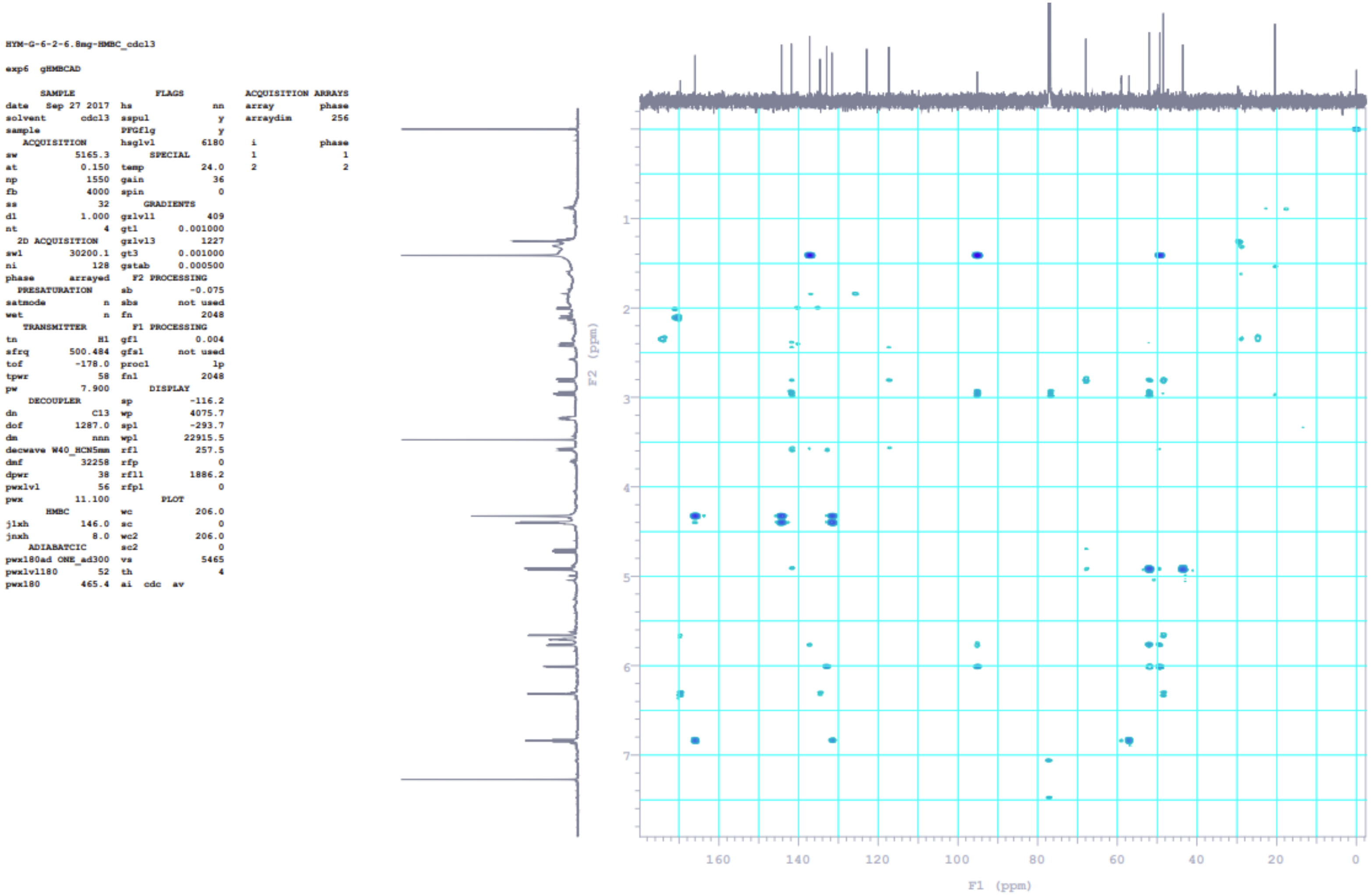

**Figure S72.** NOESY spectrum of **54** (measured in $CDCl_3$, 500 MHz).

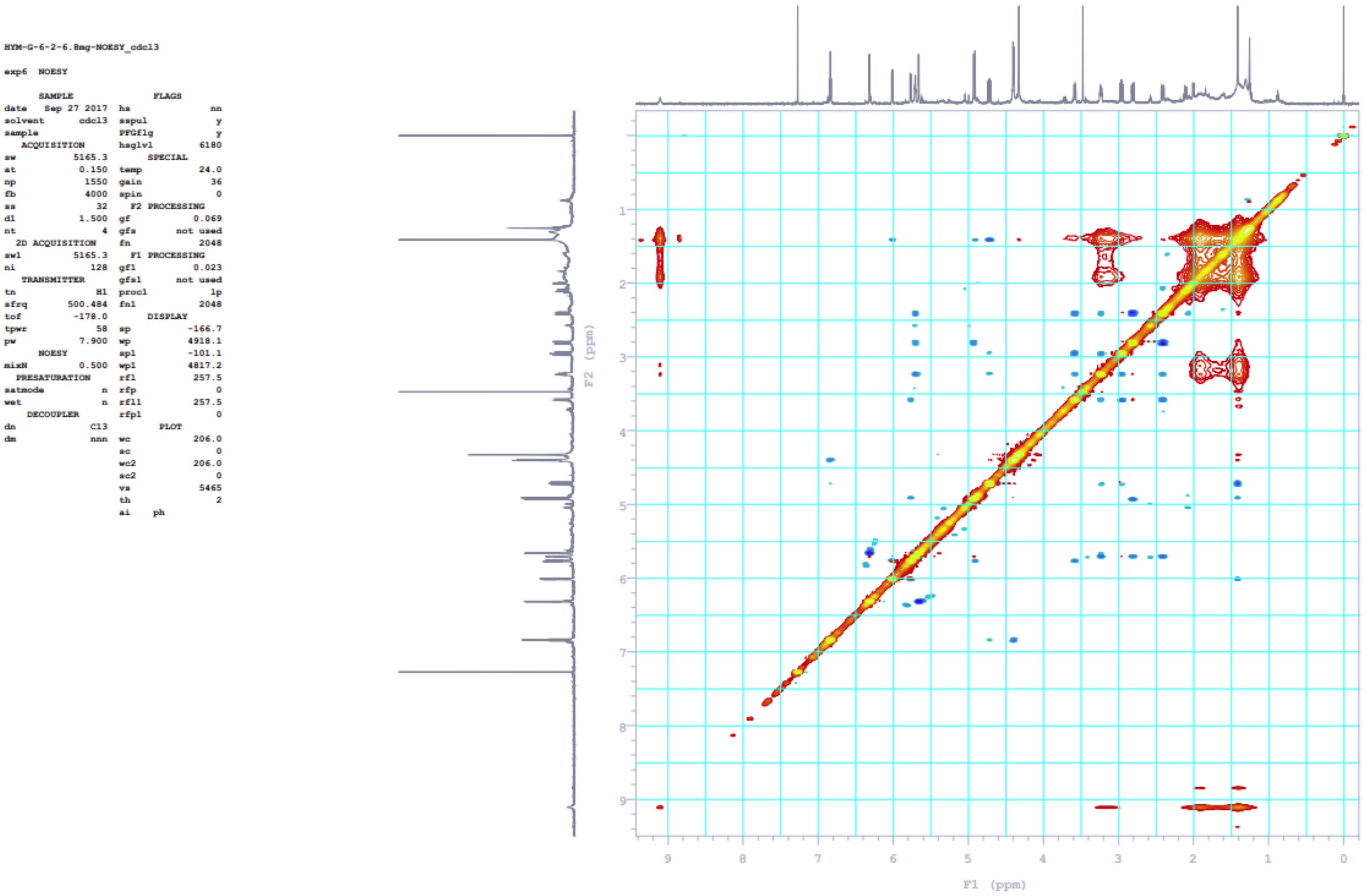

**Figure S73.** $^{1}$H NMR spectrum of **62** (measured in $CDCl_3$, 500 MHz).

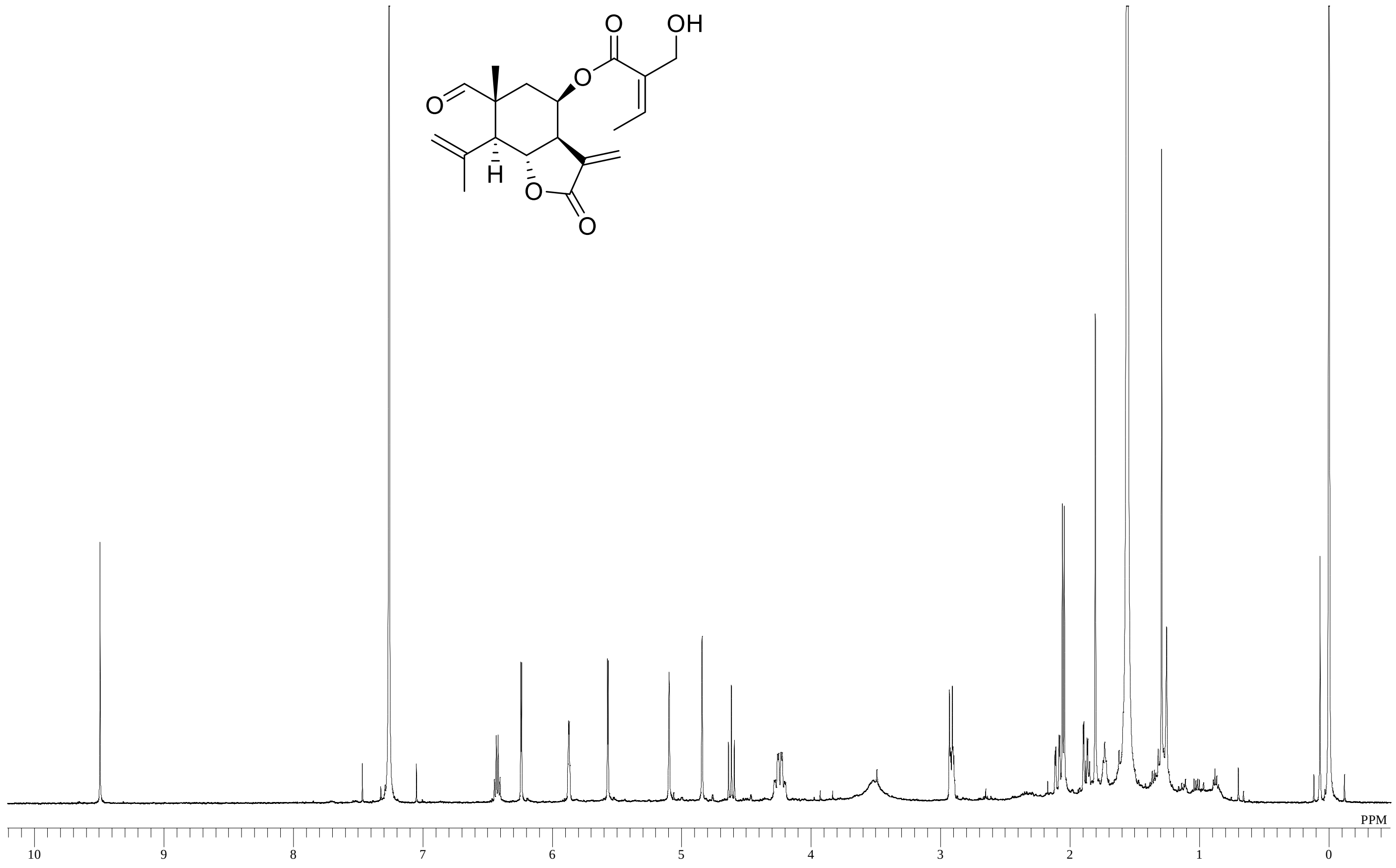

**Figure S74.** $^{13}$C NMR spectrum of **62** (measured in $CDCl_3$, 126 MHz).

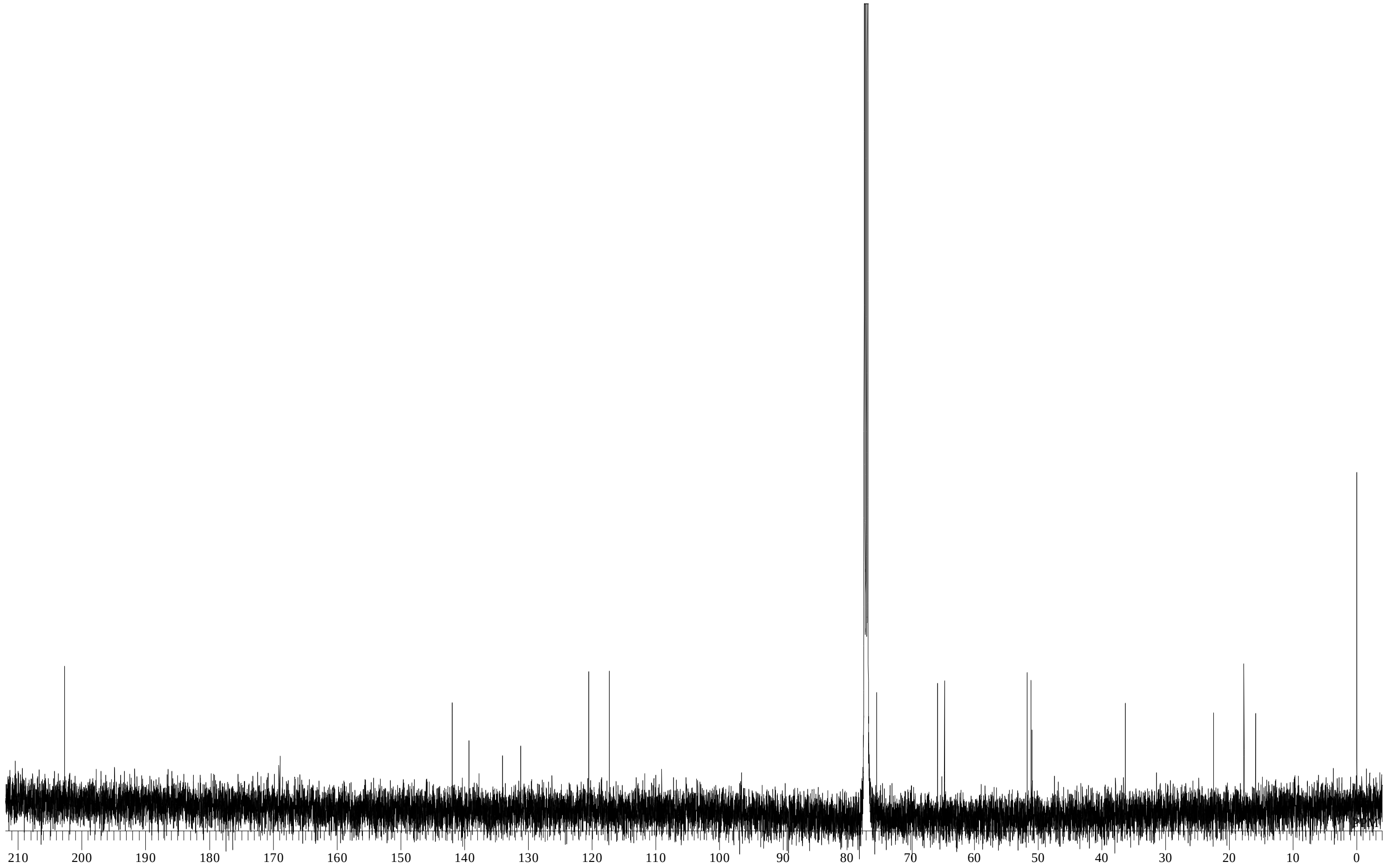

**Figure S75.** $^1$H-$^1$H COSY spectrum of **62** (measured in $CDCl_3$, 500 MHz).

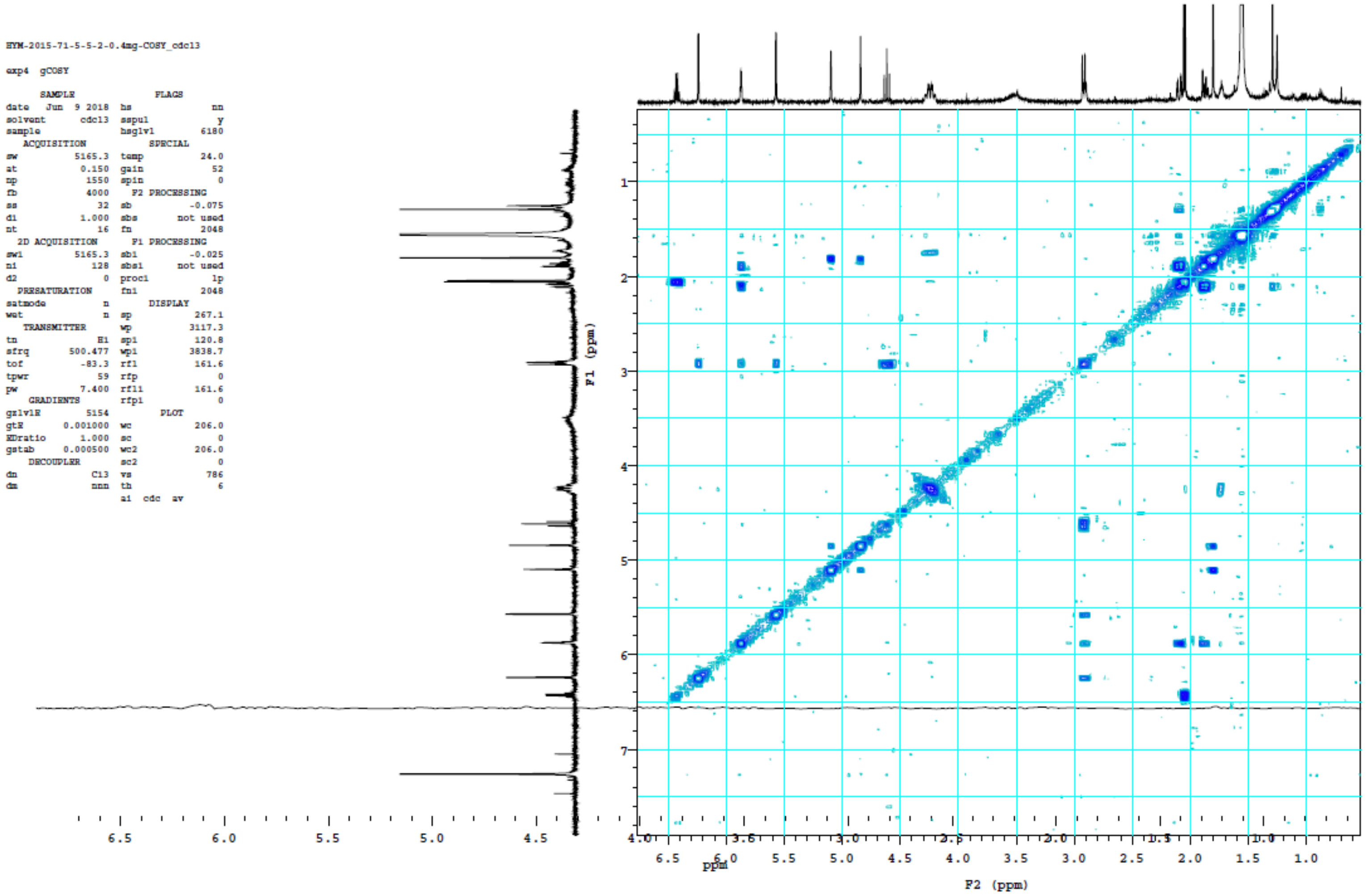

**Figure S76.** HSQC spectrum of **62** (measured in $CDCl_3$, 500 MHz).

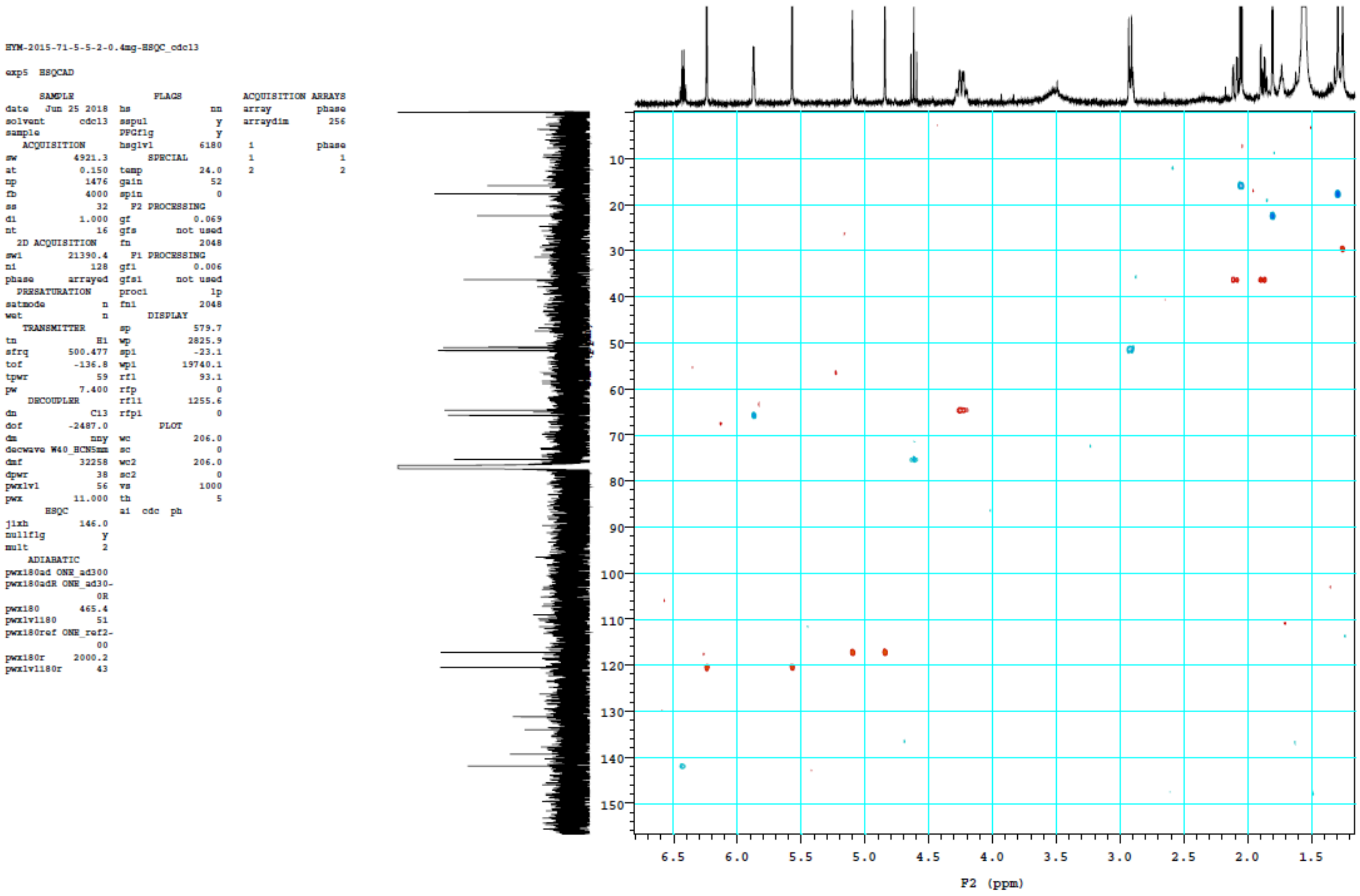

**Figure S77.** HMBC spectrum of **62** (measured in $CDCl_3$, 500 MHz).

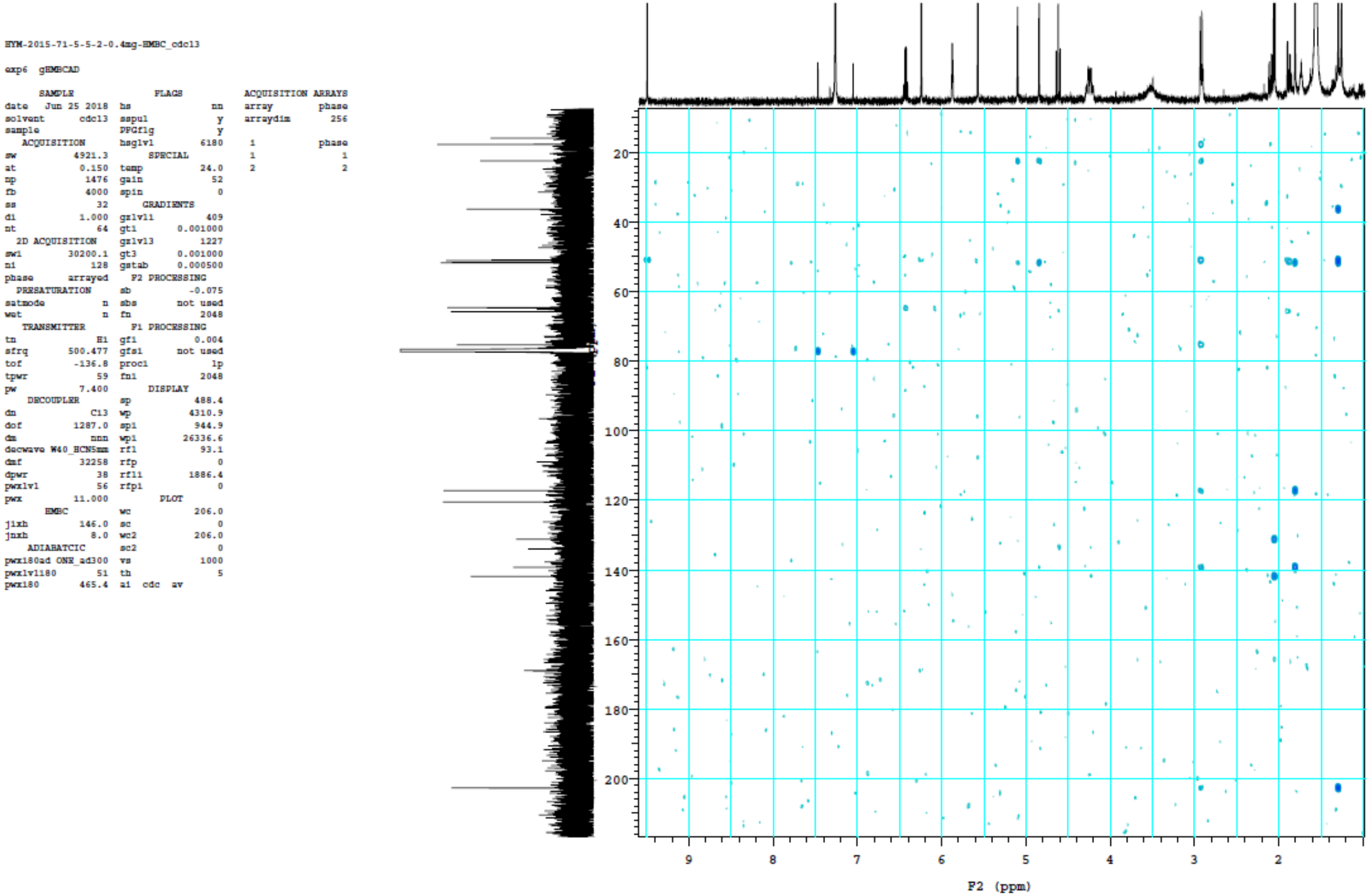

**Figure S78.** NOESY spectrum of **62** (measured in $CDCl_3$, 500 MHz).

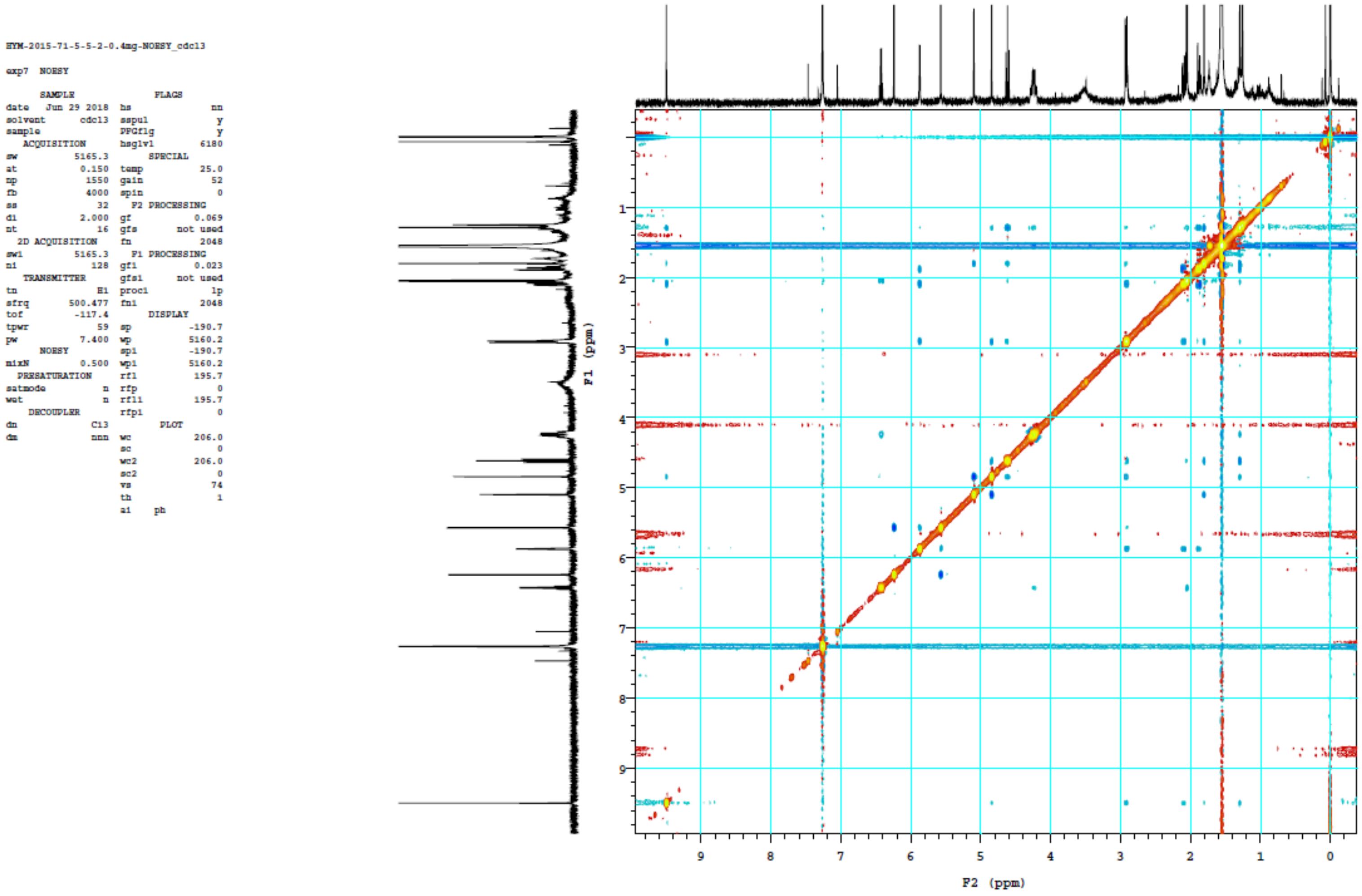

**Figure S79.** Experimental and calculated ECD spectra of **10**.

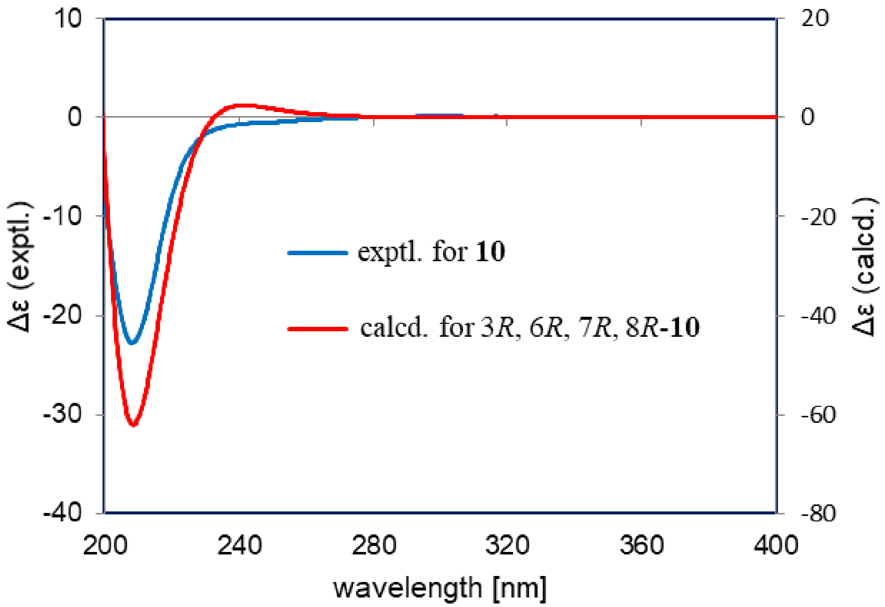

Supplement: Supplementary file 1 [file molecules-28-05107-s001.zip › molecules-2399888-supplementary.pdf]
